# Applications and Techniques for Fast Machine Learning in Science

**DOI:** 10.3389/fdata.2022.787421

**Published:** 2022-04-12

**Authors:** Allison McCarn Deiana, Nhan Tran, Joshua Agar, Michaela Blott, Giuseppe Di Guglielmo, Javier Duarte, Philip Harris, Scott Hauck, Mia Liu, Mark S. Neubauer, Jennifer Ngadiuba, Seda Ogrenci-Memik, Maurizio Pierini, Thea Aarrestad, Steffen Bähr, Jürgen Becker, Anne-Sophie Berthold, Richard J. Bonventre, Tomás E. Müller Bravo, Markus Diefenthaler, Zhen Dong, Nick Fritzsche, Amir Gholami, Ekaterina Govorkova, Dongning Guo, Kyle J. Hazelwood, Christian Herwig, Babar Khan, Sehoon Kim, Thomas Klijnsma, Yaling Liu, Kin Ho Lo, Tri Nguyen, Gianantonio Pezzullo, Seyedramin Rasoulinezhad, Ryan A. Rivera, Kate Scholberg, Justin Selig, Sougata Sen, Dmitri Strukov, William Tang, Savannah Thais, Kai Lukas Unger, Ricardo Vilalta, Belina von Krosigk, Shen Wang, Thomas K. Warburton

**Affiliations:** ^1^Department of Physics, Southern Methodist University, Dallas, TX, United States; ^2^Fermi National Accelerator Laboratory, Batavia, IL, United States; ^3^Department of Electrical and Computer Engineering, Northwestern University, Evanston, IL, United States; ^4^Department of Materials Science and Engineering, Lehigh University, Bethlehem, PA, United States; ^5^Xilinx Research, Dublin, Ireland; ^6^Department of Computer Science, Columbia University, New York, NY, United States; ^7^Department of Physics, University of California, San Diego, San Diego, CA, United States; ^8^Massachusetts Institute of Technology, Cambridge, MA, United States; ^9^Department of Electrical and Computer Engineering, University of Washington, Seattle, WA, United States; ^10^Department of Physics and Astronomy, Purdue University, West Lafayette, IN, United States; ^11^Department of Physics, University of Illinois Urbana-Champaign, Champaign, IL, United States; ^12^European Organization for Nuclear Research (CERN), Meyrin, Switzerland; ^13^Karlsruhe Institute of Technology, Karlsruhe, Germany; ^14^Institute of Nuclear and Particle Physics, Technische Universität Dresden, Dresden, Germany; ^15^Lawrence Berkeley National Laboratory, Berkeley, CA, United States; ^16^Department of Physics and Astronomy, University of Southampton, Southampton, United Kingdom; ^17^Thomas Jefferson National Accelerator Facility, Newport News, VA, United States; ^18^Department of Electrical Engineering and Computer Sciences, University of California, Berkeley, Berkeley, CA, United States; ^19^Department of Computer Science, Technical University Darmstadt, Darmstadt, Germany; ^20^Department of Bioengineering, Lehigh University, Bethlehem, PA, United States; ^21^Department of Physics, University of Florida, Gainesville, FL, United States; ^22^Department of Physics, Yale University, New Haven, CT, United States; ^23^Department of Engineering and IT, University of Sydney, Camperdown, NSW, Australia; ^24^Department of Physics, Duke University, Durham, NC, United States; ^25^Cerebras Systems, Sunnyvale, CA, United States; ^26^Birla Institute of Technology and Science, Pilani, India; ^27^Department of Electrical and Computer Engineering, University of California, Santa Barbara, Santa Barbara, CA, United States; ^28^Department of Physics, Princeton University, Princeton, NJ, United States; ^29^Department of Computer Science, University of Houston, Houston, TX, United States; ^30^Department of Physics, Universität Hamburg, Hamburg, Germany; ^31^Department of Physics and Astronomy, Iowa State University, Ames, IA, United States

**Keywords:** machine learning for science, big data, particle physics, codesign, coprocessors, heterogeneous computing, fast machine learning

## Abstract

In this community review report, we discuss applications and techniques for *fast* machine learning (ML) in science—the concept of integrating powerful ML methods into the real-time experimental data processing loop to accelerate scientific discovery. The material for the report builds on two workshops held by the Fast ML for Science community and covers three main areas: applications for fast ML across a number of scientific domains; techniques for training and implementing performant and resource-efficient ML algorithms; and computing architectures, platforms, and technologies for deploying these algorithms. We also present overlapping challenges across the multiple scientific domains where common solutions can be found. This community report is intended to give plenty of examples and inspiration for scientific discovery through integrated and accelerated ML solutions. This is followed by a high-level overview and organization of technical advances, including an abundance of pointers to source material, which can enable these breakthroughs.

## Overview

Machine learning (ML) is making a huge impact on our society and daily lives through advancements in computer vision, natural language processing, and autonomous vehicles, among others. ML is also powering scientific advances which can lead to future paradigm shifts in a broad range of domains, including particle physics, plasma physics, astronomy, neuroscience, chemistry, material science, and biomedical engineering. Scientific discoveries come from groundbreaking ideas and the capability to validate those ideas by testing nature at new scales-finer and more precise temporal and spatial resolution. This is leading to an explosion of data that must be interpreted, and ML is proving a powerful approach. The more efficiently we can test our hypotheses, the faster we can achieve discovery. To fully unleash the power of ML and accelerate discoveries, it is necessary to embed it into our scientific process, into our instruments and detectors.

It is in this spirit that the Fast Machine Learning for Science community^1^ has been built. Two workshops have also been organized through this growing community and are the source for this report. The community brings together an extremely wide-ranging group of domain experts who would rarely interact as a whole. One of the underlying benefits of ML is the portability and general applicability of the techniques that can enable experts from seemingly unrelated domains to find a common language. Scientists and engineers from particle physicists to networking experts and biomedical engineers are represented and can interact with experts in fundamental ML techniques and compute systems architects.

This report aims to summarize the progress in the community to understand how our scientific challenges overlap and where there are potential commonalities in data representations, ML approaches, and technology, including hardware and software platforms. Therefore, **the content of the report includes the following: descriptions of a number of different scientific domains including existing work and applications for embedded ML; potential overlaps across scientific domains in data representation or system constraints; and an overview of state-of-the-art techniques for efficient machine learning and compute platforms, both cutting-edge and speculative technologies**.

Necessarily, such a broad scope of topics *cannot* be comprehensive. For the scientific domains, we note that the contributions are *examples* of how ML methods are currently being or planned to be deployed. We hope that giving a glimpse into specific applications will inspire readers to find more novel use-cases and potential overlaps. The summaries of state-of-the-art techniques we provide relate to rapidly developing fields and, as such, may become out of date relatively quickly. The goal is to give non-experts an overview and taxonomy of the different techniques and a starting point for further investigation. To be succinct, we rely heavily on providing references to studies and other overviews while describing most modern methods.

We hope the reader finds this report both instructive and motivational. Feedback and input to this report, and to the larger community, are welcome and appreciated.

## 1. Introduction

In pursuit of scientific advancement across many domains, experiments are becoming exceedingly sophisticated in order to probe physical systems at increasingly smaller spatial resolutions and shorter timescales. These order of magnitude advancements have lead to explosions in both data volumes and richness leaving domain scientists to develop novel methods to handle growing data processing needs.

Simultaneously, machine learning (ML), or the use of algorithms that can learn directly from data, is leading to rapid advancements across many scientific domains (Carleo et al., 2019). Recent advancements have demonstrated that deep learning (DL) architectures based on structured deep neural networks are versatile and capable of solving a broad range of complex problems. The proliferation of large datasets like ImageNet (Russakovsky et al., 2015), computing, and DL software has led to the exploration of many different DL approaches each with their own advantages.

In this review paper, we will focus on the fusion of ML and experimental design to solve critical scientific problems by accelerating and improving data processing and real-time decision-making. We will discuss the myriad of scientific problems that require fast ML, and we will outline unifying themes across these domains that can lead to general solutions. Furthermore, we will review the current technology needed to make ML algorithms run fast, and we will present critical technological problems that, if solved, could lead to major scientific advancements. An important requirement for such advancements in science is the need for openness. It is vital for experts from domains that do not often interact to come together to develop transferable solutions and work together to develop open-source solutions.

Much of the advancements within ML over the past few years have originated from the use of heterogeneous computing hardware. In particular, the use of graphics processing units (GPUs) has enabled the development of large DL algorithms (Raina et al., 2009; Cireşan et al., 2010; Krizhevsky et al., 2012). The ability to train large artificial intelligence (AI) algorithms on large datasets has enabled algorithms that are capable of performing sophisticated tasks. In parallel with these developments, new types of DL algorithms have emerged that aim to reduce the number of operations so as to enable fast and efficient AI algorithms (Box 1).

Box 1 **Fast machine learning in science.**

Within this review paper, we refer to the concept of ***Fast Machine Learning in Science*** as the integration of ML into the experimental data processing infrastructure to enable and accelerate scientific discovery. Fusing powerful ML techniques with experimental design decreases the “time to science” and can range from embedding real-time feature extraction to be as close as possible to the sensor all the way to large-scale ML acceleration across distributed grid computing datacenters. The overarching theme is to lower the barrier to advanced ML techniques and implementations to make large strides in experimental capabilities across many seemingly different scientific applications. Efficient solutions require collaboration between domain experts, machine learning researchers, and computer architecture designers.

**Figure 1:**
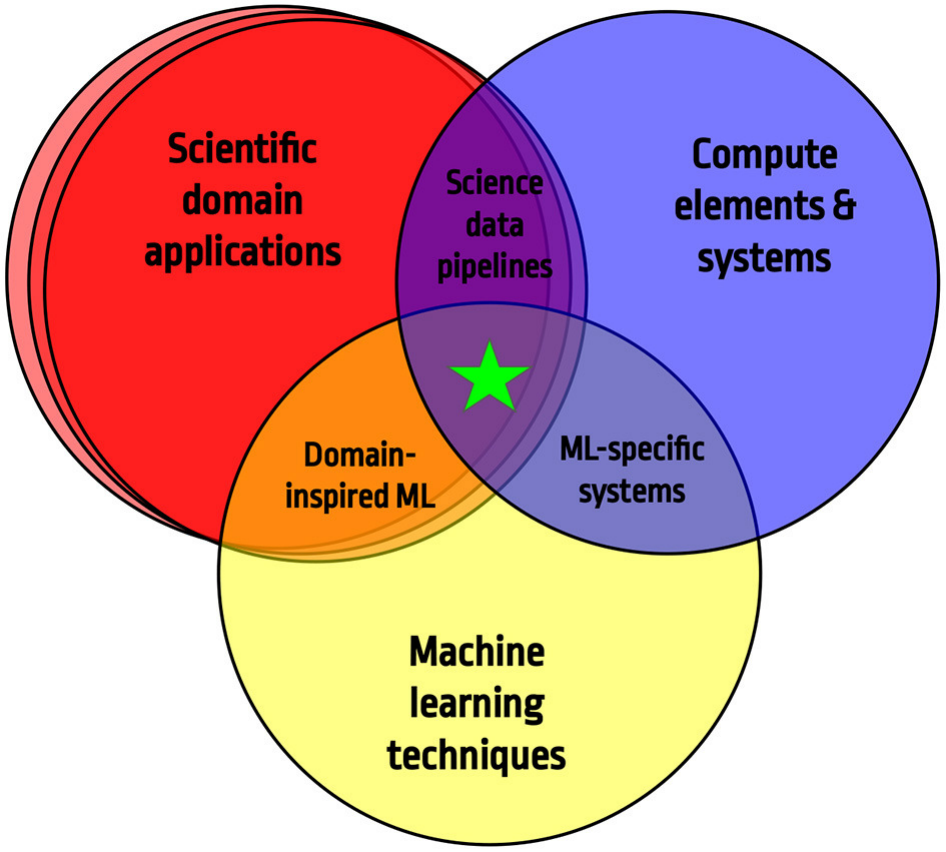
The concept behind this review paper is to find the confluence of domain-specific challenges, machine learning, and experiment and computer system architectures to accelerate science discovery.

This paper is a review of the second annual Fast Machine Learning conference (University, 2020) and will build on the materials presented at this conference. It brings together experts from multiple scientific domains ranging from particle physicists to material scientists to health monitoring researchers with machine learning experts and computer systems architects. Figure 1 illustrates the spirit of the workshop series on which this paper is inspired and the topics covered in subsequent sections.

As ML tools have become more sophisticated, much of the focus has turned to building very large algorithms that solve complicated problems, such as language translation and voice recognition. However, in the wake of these developments, a broad range of scientific applications have emerged that can benefit greatly from the rapid developments underway. Furthermore, these applications have diversified as people have to come to realize how to adapt their scientific approach so as to take advantage of the benefits originating from the AI revolution. This can include the capability of AI to classify events in real time, such as the identification of a collision of particles or a merger of gravitational waves. It can also include systems control, such as the response control from feedback mechanisms in plasmas and particle accelerators. The latency, bandwidth, and throughput restrictions and the reasons for such restrictions differ within each system. However, in all cases, accelerating ML is a driver in the design goal.

The design of low latency algorithms differs from other AI implementations in that we must tailor specific processing hardware to the task at hand to increase the overall algorithm performance. In particular, certain processor cores have been configured for optimized sparse matrix multiplications. Others have been optimized to maximize the total amount of compute. Processor design, and the design of algorithms around processors, often referred to as hardware ML co-design, is the focus of the work in this review. For example, in some cases, ultra-low latency inference times are needed to perform scientific measurements. One must efficiently design the algorithm to optimally utilize the hardware constraints available while preserving the algorithm performance within desired experimental requirements. This is the essence of hardware ML co-design.

The contents of this review are laid out as follows. In the Section 2, we will explore a broad range of scientific problems where Fast ML can act as a disruptive technology to the status quo and lead to a significant change in how we process data. Domain experts from seemingly different domains are examined. In Section 3, we describe data representations and experimental platform choices are common to many types of experiments. We will connect how Fast ML solutions can be generalized to low latency, highly resource-efficient, and domain-specific deep learning inference for many scientific applications. Finally in Section 4, to achieve this requires optimized hardware ML co-design from the algorithm design to the system architecture. We provide an overview of state-of-the-art techniques to train neural networks optimized for both performance and speed, survey various compute architectures to meet the needs of the experimental design and outline software solutions that optimize and enable the hardware deployment.

The goal of this paper is to bring together scientific opportunities, common solutions, and state-of-the-art technology into one single narrative. We hope this can contribute to accelerating the deployment of potentially transformative ML solutions to a broad range of scientific fields going forward.

## 2. Exemplars of Domain Applications

As scientific ecosystems grow rapidly in their speed and scale, new paradigms for data processing and reduction need to be integrated into system-level design. In this section, we explore requirements for accelerated and sophisticated data processing. Implementations of fast machine learning can appear greatly varied across domains and architectures but yet can have similar underlying data representations and needs for integrating machine learning. We enumerate here a broad sampling of scientific domains across seemingly unrelated tasks including their existing techniques and future needs. This will then lead to the next section where we discuss overlaps and common tasks.

We note here that this section has an emphasis on challenges addressed with deep learning techniques being proposed to address increasingly complex datasets in scientific applications, while sometimes referring to other classic ML algorithms. However, in all of these use-cases, there is understably a large history of domain algorithms and other classic, “shallow”, ML algorithms that have been developed. For example, see discussion of classic ML methods in Albertsson et al. (2018) and even the use of Boosted Decision Trees in real-time electronics systems (Gligorov and Williams, 2013). The performance and robustness of deep learning algorithms should be compared and understood with respect to previous methods, and similarly for simpler vs. more complex deep learning algorithms. A full survey of classic ML, deep learning, and domain algorithms for given applications, though, we consider beyond the scope of this paper.

In this section, we first have a detailed description of examples of Fast ML techniques being deployed at experiments for the Large Hadron Collider. Much rapid development has occurred for these experiments recently and gives an exemplar for how broad advancements can be made across various aspects of a specific domain. Then the following subsections will be briefer but lay out key challenges and areas of existing and potential applications of Fast ML across a number of other scientific domains.

### 2.1. Large Hadron Collider

The Large Hadron Collider (LHC) at CERN is the world's largest and highest-energy particle accelerator, where collisions between bunches of protons occur every 25 ns. To study the products of these collisions, several detectors are located along the ring at interaction points. The aim of these detectors is to measure the properties of the Higgs boson (Aad et al., 2012; Chatrchyan et al., 2012) with high precision and to search for new physics phenomena beyond the standard model of particle physics. Due to the extremely high frequency of 40 MHz at which proton bunches collide, the high multiplicity of secondary particles, and the large number of sensors, the detectors have to process and store data at enormous rates. For the two multipurpose experiments, CMS and ATLAS (Aad, 2008), comprised of tens of millions of readout channels, these rates are of the order of 100 Tb/s. Processing and storing this data presents severe challenges that are among the most critical for the execution of the LHC physics program.

The approach implemented by the detectors for data processing consists of an online processing stage, where the event is selected from a buffer and analyzed in real time, and an offline processing stage, in which data have been written to disk and are more thoroughly analyzed with sophisticated algorithms. The online processing system, called the *trigger*, reduces the data rate to a manageable level of 10Gb/s to be recorded for offline processing. The trigger is typically divided into multiple tiers. Due to the limited size of the on-detector buffers, the first tier (Level-1 or L1) utilizes FPGAs and ASICs capable of executing the filtering process with a maximum latency of O(1) μs. At the second stage, the high-level trigger (HLT), data are processed on a CPU-based computing farm located at the experimental site with a latency of up to 100 ms. Finally, the complete offline event processing is performed on a globally distributed CPU-based computing grid.

Maintaining the capabilities of this system will become even more challenging in the near future. In 2027, the LHC will be upgraded to the so-called High-Luminosity LHC (HL-LHC) where each collision will produce 5–7 times more particles, ultimately resulting in a total amount of accumulated data that will be one order of magnitude higher than achieved with the present accelerator. At the same time, the particle detectors will be made larger, more granular, and capable of processing data at ever-increasing rates. Therefore, the physics that can be extracted from the experiments will be limited by the accuracy of algorithms and computational resources.

Machine learning technologies offer promising solutions and enhanced capabilities in both of these areas, thanks to their capacity for extracting the most relevant information from high-dimensional data and to their highly parallelizable implementation on suitable hardware. In addition, there are even some early investigations exploring potential applications of machine learning using quantum computing (Wu et al., 2021). It is expected that a new generation of algorithms, if deployed at all stages of data-processing systems at the LHC experiments, will play a crucial part in maintaining, and hopefully improving, the physics performance. In the following sections, a few examples of the application of machine learning models to physics tasks at the LHC are reviewed, together with novel methods for their efficient deployment in both the real-time and offline data processing stages.

#### 2.1.1. Event Reconstruction

The reconstruction of proton-proton collision events in the LHC detectors involves challenging pattern recognition tasks, given the large number [O(1,000)] of secondary particles produced and the high detector granularity. Specialized detector sub-systems and algorithms are used to reconstruct the different types and properties of particles produced in collisions. For example, the trajectories of charged particles are reconstructed from space point measurements in the inner silicon detectors, and the showers arising from particles traversing the calorimeters are reconstructed from clusters of activated sensors.

Traditional algorithms are highly tuned for physics performance in the current LHC collision environment, but are inherently sequential and scale poorly to the expected HL-LHC conditions. It is thus necessary to revisit existing reconstruction algorithms and ensure that both the physics and computational performance will be sufficient. Deep learning solutions are currently being explored for pattern recognition tasks, as a significant speedup can be achieved when harnessing heterogeneous computing and parallelizable and efficient ML that exploits AI-dedicated hardware. In particular, modern architectures such as graph neural networks (GNNs) are being explored for the reconstruction of particle trajectories, showers in the calorimeter as well as of the final individual particles in the event. Much of the following work has been conducted using the TrackML dataset (Calafiura et al., 2018), which simulates a generalized detector under HL-LHC-like pileup conditions. Quantifying the performance of these GNNs in actual experimental data is an ongoing point of study.

For reconstructing showers in calorimeters, GNNs have been found to predict the properties of the original incident particle with high accuracy starting from individual energy deposits. The work in Gray et al. (2020) proposes a graph formulation of pooling to dynamically learn the most important relationships between data via an intermediate clustering, and therefore removing the need for a predetermined graph structure. When applied to the CMS electromagnetic calorimeter, with single detector hits as inputs to predict the energy of the original incident particle, a 10% improvement is found over the traditional boosted decision tree (BDT) based approach.

GNNs have been explored for a similar calorimeter reconstruction task for the high-granularity calorimeters that will replace the current design for HL-LHC. The task will become even more challenging as such detectors will feature irregular sensor structure and shape (e.g., hexagonal sensor cells for CMS CMS Collaboration, 2017), high occupancy, and an unprecedented number of sensors. For this application, architectures such as EdgeConv (Wang et al., 2018b) and GravNet/GarNet (Qasim et al., 2019) have shown promising performance in the determination of the properties of single showers, yielding excellent energy resolution and high noise rejection (Ju et al., 2020). While these preliminary studies were focused on scenarios with low particle multiplicities, the scalability of the clustering performance to more realistic collision scenarios is still a subject of active development.

**Figure 2:**
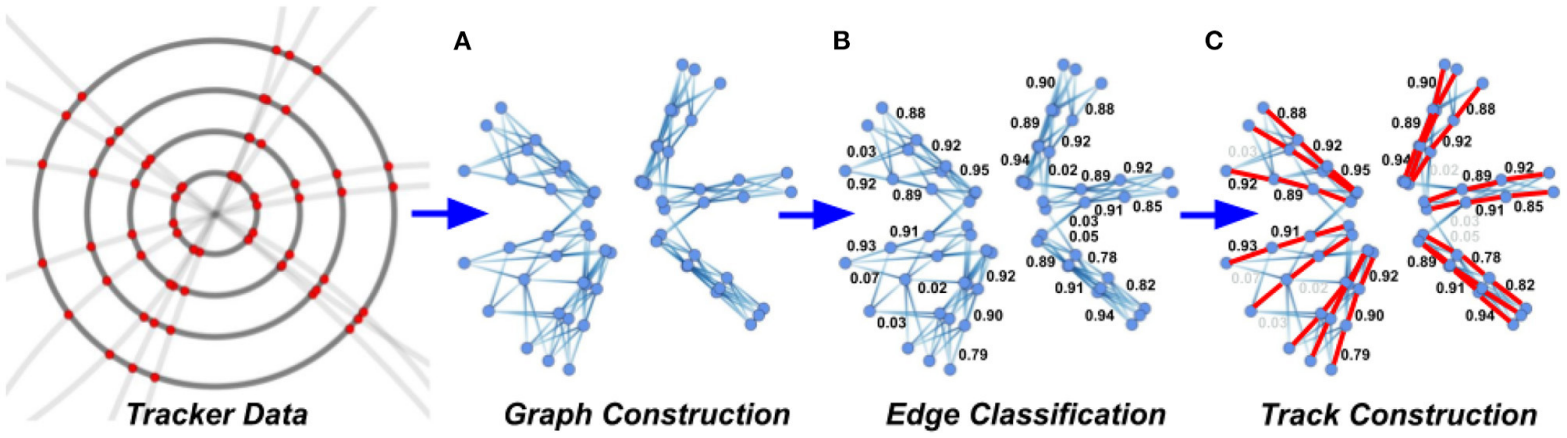
High-level overview of the stages in a GNN-based tracking pipeline. Only a subset of the typical edge weights are shown for illustration purposes. **(A)** Graph construction, **(B)** edge classification, and **(C)** track construction.

GNNs have also been extensively studied for charged particle tracking (the task of identifying and reconstructing the trajectories of individual particles in the detector) (Farrell et al., 2018; Tsaris et al., 2018; Duarte and Vlimant, 2020; Ju et al., 2020). The first approaches to this problem typically utilized edge-classification GNNs in a three-step process: graphs are constructed by algorithmically constructing edges between tracker hits in a point cloud, the graphs are processed through a GNN to predict edge weights (true edges that are part of true particle trajectories should be highly weighted and false edges should be lowly rated), and finally, the selected edges are grouped together to generate high-weight sub-graphs which form full track candidates, as shown in Figure 2.

There have been several studies building upon and optimizing this initial framework. The ExaTrkX collaboration has demonstrated performance improvements by incorporating a recurrent GNN structure (Ju et al., 2020) and re-embedding graphs prior to training the GNNs (Choma et al., 2020). Other work has shown that using an Interaction Network architecture (Battaglia et al., 2016) can substantially reduce the number of learnable parameters in the GNN (DeZoort et al., 2021); the authors also provide comprehensive comparisons between different graph construction and track building algorithms. Recent work has also explored alternate approaches that combine graph building, GNN inference, and track construction into a single algorithm that is trainable end-to-end; in particular, instance segmentation architectures have generated promising results (Thais and DeZoort, 2021).

Finally, a novel approach based on GNNs (Pata et al., 2021) has been proposed as an alternative solution to the so-called particle-flow algorithm that is used by LHC experiments to optimally reconstruct each individual particle produced in a collision by combining information from the calorimeters and the tracking detectors (Sirunyan et al., 2017). The new GNN algorithm is found to offer comparable performance for charged and neutral hadrons to the existing reconstruction algorithm. At the same time, the inference time is found to scale approximately linearly with the particle multiplicity, which is promising for its ability to maintain computing costs within budget for the HL-LHC. Further improvements to this original approach are currently under study, including an event-based loss, such as the object condensation approach. Second, a complete assessment of the physics performance remains to be evaluated, including reconstruction of rare particles and other corners of the phase space. Finally, it remains to be understood how to optimize and coherently interface this with the ML-based approach proposed for tasks downstream and upstream in the particle-level reconstruction.

#### 2.1.2. Event Simulation

The extraction of results from LHC data relies on a detailed and precise simulation of the physics of proton-proton collisions and of the response of the detector. In fact, the collected data are typically compared to a reference model, representing the current knowledge, in order to either confirm or disprove it. Numerical models, based on Monte Carlo (MC) methods, are used to simulate the interaction between elementary particles and matter, while the Geant4 toolkit is employed to simulate the detectors. These simulations are generally very CPU intensive and require roughly half of the experiment's computing resources, with this fraction expected to increase significantly for the HL-LHC.

Novel computational methods based on ML are being explored so as to perform precise modeling from particle interactions to detector readouts and response while maintaining feasible computing budgets for HL-LHC. In particular, numerous works have focused on the usage of generative adversarial networks or other state-of-the-art generative models to replace computationally intensive fragments of MC simulation, such as modeling of electromagnetic showers (de Oliveira et al., 2017; Paganini et al., 2018a,b), reconstruction of jet images (Musella and Pandolfi, 2018) or matrix element calculations (Bendavid, 2017). In addition, the usage of ML generative models on end-to-end analysis-specific fast simulations have also been investigated in the context of Drell-Yan (Hashemi et al., 2019), dijet (Di Sipio et al., 2019), and W+jets (Chen et al., 2020) production. These case-by-case proposals serve as proof-of-principle examples for complementary data augmentation strategy for LHC experiments.

#### 2.1.3. Heterogeneous Computing

State-of-the-art deep learning models are being explored for the compute-intensive reconstruction of each collision event at the LHC. However, their efficient deployment within the experiments' computing paradigms is still a challenge, despite the potential speed-up when the inference is executed on suitable AI-dedicated hardware. In order to gain from a parallelizable ML-based translation of traditional and mostly sequential algorithms, a heterogeneous computing architecture needs to be implemented in the experiment infrastructure. For this reason, comprehensive exploration of the use of CPU+GPU (Krupa et al., 2020) and CPU+FPGA (Duarte et al., 2019; Rankin et al., 2020) heterogeneous architectures was made to achieve the desired acceleration of deep learning inference within the data processing workflow of LHC experiments. These works demonstrated that the acceleration of machine learning inference “as a service” represents a heterogeneous computing solution for LHC experiments that potentially requires minimal modification to the current computing model.

In this approach, the ML algorithms are transferred to a co-processor on an independent (local or remote) server by reconfiguring the CPU node to communicate with it through asynchronous and non-blocking inference requests. With the inference task offloaded on demand to the server, the CPU can be dedicated to performing other necessary tasks within the event. As one server can serve many CPUs, this approach has the advantage of increasing the hardware cost-effectiveness to achieve the same throughput when comparing it to a direct-connection paradigm. It also facilitates the integration and scalability of different types of co-processor devices, where the best one is chosen for each task.

Finally, existing open-source frameworks that have been optimized for fast DL on several different types of hardware can be exploited for a quick adaptation to LHC computing. In particular, one could use the Nvidia Triton Inference Server within a custom framework, so-called Services for Optimized Network Inference on Co-processors (SONIC), to enable remote gRPC calls to either GPUs or FPGAs within the experimental software, which then only has to handle the input and output conversion between event data format and inference server format. The integration of this approach within the CMS reconstruction software has been shown to lead to a significant overall reduction in the computing demands both at the HLT and offline.

#### 2.1.4. Real-Time Analysis at 40 MHz

Bringing deep learning algorithms to the Level-1 hardware trigger is an extremely challenging task due to the strict latency requirement and the resource constraints imposed by the system. Depending on which part of the system an algorithm is designed to run on, a latency down to O(10) ns might be required. With O(100)  processors running large-capacity FPGAs, processing thousands of algorithms in parallel, dedicated FPGA-implementations are needed to make ML algorithms as resource-efficient and fast as possible. To facilitate the design process and subsequent deployment of highly parallel, highly compressed ML algorithms on FPGAs, dedicated open-source libraries have been developed: hls4ml and Conifer. The former, hls4ml, provides conversion tools for deep neural networks, while Conifer aids the deployment of Boosted Decision Trees (BDTs) on FPGAs. Both libraries, as well as example LHC applications, will be described in the following.

The hls4ml library (Duarte et al., 2018; Coelho et al., 2020; Loncar et al., 2020; Aarrestad et al., 2021) converts pre-trained ML models into ultra low-latency FPGA or ASIC firmware with little overhead required. Integration with the Google QKeras library (Coelho, 2019) allows users to design aggressively quantized deep neural networks and train them quantization-aware (Coelho et al., 2020) down to 1 or 2 bits for weights and activations (Loncar et al., 2020). This step results in highly resource-efficient equivalents of the original model, sacrificing little to no accuracy in the process. The goal of this joint package is to provide a simple two-step approach going from a pre-trained floating point model to FPGA firmware. The hls4ml library currently provides support for several commonly used neural network layers like fully connected, convolutional, batch normalization, pooling, as well as several activation functions. These implementations are already sufficient to provide support for the most common architectures envisioned for deployment at L1.

Some first examples of machine learning models designed for the L1 trigger are based on fully connected layers, and they are proposed for tasks such as the reconstruction and calibration of final objects or lower-level inputs like trajectories, vertices, and calorimeter clusters (CERN, 2020). One example of a convolutional NN (CNN) architecture targeting the L1 trigger is a dedicated algorithm for the identification of long-lived particles (Alimena et al., 2020). Here, an attempt is made to efficiently identify showers from displaced particles in a high-granularity forward calorimeter. The algorithm is demonstrated to be highly efficient down to low energies while operating at a low trigger rate. Traditionally, cut-based selection algorithms have been used for these purposes, in order to meet the limited latency- and resource budget. However, with the advent of tools like hls4ml and QKeras, ML alternatives are being explored to improve the sensitivity to such physics processes while maintaining latency and resources in the available budget.

More recently, (variational) auto-encoders (VAEs or AEs) are being considered for the detection of “anomalous” collision events, i.e., events that are not produced by standard physics processes but that could be due instead to unexpected processes not yet explored at colliders. Such algorithms have been proposed for both the incoming LHC run starting in 2022 as well as for the future high-luminosity runs where more granular information will be available. The common approach uses global information about the event, including a subset of individual produced particles or final objects such as jets as well as energy sums. The algorithm trained on these inputs is then used to classify the event as anomalous if surpassing a threshold on the degree of anomaly (typically the loss function), ultimately decided upon the available bandwidth. Deploying a typical variational autoencoder is impossible in the L1-trigger since the bottleneck layer involves Gaussian random sampling. The explored solution is therefore to only deploy the encoder part of the network and do inference directly from the latent dimension. Another possibility is to deploy a simple auto-encoder with the same architecture and do inference computing the difference between output and input. However, this would require buffering a copy of the input for the duration it takes the auto-encoder to process the input. For this reason, the two methods are being considered and compared in terms of accuracy over a range of new physics processes, as well as latency and resources. Finally, another interesting aspect of the hls4ml tool is the capability for users to easily add custom layers that might serve a specific task not captured by the most common layers supported in the library. One example of this is compressed distance-weighted graph networks (Iiyama et al., 2021), where a graph network block called a *GarNet layer* takes as input a set of V vertices, each of which has *F*_*in*_ features, and returns the same set of vertices with *F*_*out*_ features. To keep the dimensionality of the problem at a manageable level, the input features of each vertex are encoded and aggregated at *S* aggregators. Message-passing is only performed between vertices and a limited set of aggregators, and not between all vertices, significantly reducing the network size. In Iiyama et al. (2021), an example task of pion and electron identification and energy regression in a 3D calorimeter is studied. A total inference latency of O(100) ns is reported, satisfying the L1 requirement of O(1) μs latency. The critical resource is digital signal processing (DSP) units, where 29% of the DSPs are in use by the algorithm. This can be further reduced by taking advantage of quantization-aware training with QKeras. Another example of a GNN architecture implemented on FPGA hardware using hls4ml is presented in Heintz et al. (2020). This work shows that a compressed GNN can be deployed on FPGA hardware within the latency and resources required by L1 trigger system for the challenging task of reconstructing the trajectory of charged particles.

In many cases, the task to be performed is simple enough that a boosted decision tree (BDT) architecture suffices to solve the problem. As of today, BDTs are still the most commonly used ML algorithm for LHC experiments. To simplify the deployment of these, the library Conifer (Summers et al., 2020) has been developed. In Conifer, the BDT implementation targets extreme low latency inference by executing all trees, and all decisions within each tree, in parallel. BDTs and random forests can be converted from scikit-learn (Pedregosa et al., 2011), XGBoost (Chen and Guestrin, 2016), and TMVA (Therhaag and Team, 2012), with support for more BDT training libraries planned. For a large part of the field, though, the frameworks that are currently supported are the most widely used.

There are several ongoing projects at LHC which plan to deploy BDTs in the Level-1 trigger using Conifer. One example is a BDT designed to provide an estimate of the *track quality*, by learning to identify tracks that are reconstructed in error, and do not originate from a real particle (Savard, 2020).

While the accuracy and resource usage are similar between a BDT and a DNN, the latency is significantly reduced for a BDT architecture. The algorithm is planned to be implemented in the CMS Experiment for the data-taking period beginning in 2022.

Rather than relying on open source libraries such as hls4ml or Conifer, which are based on high-level synthesis tools from FPGA vendors, other approaches are being considered based directly on hardware description languages, such as VHDL (Nottbeck et al., 2019; Fritzsche, 2020). One example is the application of ML for the real-time signal processing of the ATLAS Liquid Argon calorimeter (ATL, 1996). It has been shown that with upgraded capabilities for the HL-LHC collision environment the conventional signal processing, which applies an optimal filtering algorithm (Cleland and Stern, 1994), will lose its performance due to the increase of overlapping signals. More sophisticated DL methods have been found to be more suitable to cope with these challenges being able to maintain high signal detection efficiency and energy reconstruction. More specifically, studies based on simulation (Madysa, 2019) of dilated convolutional neural networks showed promising results. An implementation of this architecture for FPGA is designed using VHDL (Fritzsche, 2020) to meet the strict requirements on latency and resources required by the L1 trigger system. The firmware runs with a multiple of the bunch crossing frequency to reuse hardware resources by implementing time-division multiplexing while using pipeline stages, the maximum frequency can be increased. Furthermore, DSPs are chained up to perform the MAC operation in between two layers efficiently. In this way, a core frequency of more than 480 MHz could be reached, corresponding to 12 times the bunch crossing frequency.

#### 2.1.5. Bringing ML to Detector Front-End

While LHC detectors grow in complexity to meet the challenging conditions of higher-luminosity environments, growing data rates prohibit transmission of full event images off-detector for analysis by conventional FPGA-based trigger systems. As a consequence, event data must be compressed on-detector in low-power, radiation-hard ASICs while sacrificing minimal physics information.

Traditionally this has been accomplished by simple algorithms, such as grouping nearby sensors together so that only these summed “super-cells” are transmitted, sacrificing the fine segmentation of the detector. Recently, an autoencoder-based approach has been proposed, relying instead on a set of machine-learned radiation patterns to more efficiently encode the complete calorimeter image via a CNN. Targeting the CMS high-granularity endcap calorimeter (HGCal) (CMS Collaboration, 2017) at the HL-LHC, the algorithm aims to achieve higher-fidelity electromagnetic and hadronic showers, critical for accurate particle identification.

The on-detector environment (the ECON-T concentrator ASIC; CMS Collaboration, 2017) demands a highly-efficient CNN implementation; a compact design should be thoroughly optimized for limited-precision calculations via quantization-aware training tools (Coelho et al., 2021). Further, to automate the design, optimization, and validation of the complex NN circuit, HLS-based tool flows (Duarte et al., 2018) may be adapted to target the ASIC form factor. Finally, as the front-end ASIC cannot be completely reprogrammed in the manner of an FPGA, a mature NN design is required from the time of initial fabrication. However, adaptability to changing run conditions and experimental priorities over the lifetime of the experiment motivate the implementation of all NN weights as configurable registers accessible via the chip's slow-control interface.

### 2.2. High Intensity Accelerator Experiments

#### 2.2.1. ML-Based Trigger System at the Belle II Experiment

*Context:* The Belle II experiment in Japan (Abe et al., 2010; Altmannshofer et al., 2019) is engaged in the search for physics phenomena that cannot be explained by the Standard Model. Electrons and positrons are accelerated at the SuperKEKB particle accelerator to collide at the interaction point located inside of the Belle II detector. The resulting decay products are continually measured by the detector's heterogeneous sensor composition. The resulting data is then stored offline for detailed analysis.

*Challenges:* Due to the increasing luminosity (target luminosity is 8 × 10^35^cm^−2^s^−1^) most of the recorded data is from unwanted but unavoidable background reactions, rather than electron-positron annihilation at the interaction point. Not only is storing all the data inefficient due to the high background rates, but it is also not feasible to build an infrastructure that stores all the generated data. A multilevel trigger system is used as a solution to decide online which recorded events are to be stored.

*Existing and Planned Work:* The Neural Network z-Vertex Trigger (NNT) described used at Belle II is a deadtime-free level 1 (L1) trigger that identifies particles by estimating their origin along the beampipe. For the whole L1 trigger process, from data readout to the decision, a real-time 5μs time budget is given to avoid dead-time (Lai et al., 2020b). Due to the time cost of data pre-processing and transmission, the NNT needs to provide a decision within 300 ns processing time.

The task of the NNT is to estimate the origin of a particle track so that it can be decided whether it originates from the interaction point or not. For this purpose, a multilayer perceptron (MLP) implemented on a Xilinx Virtex 6 XC6VHX380T FPGA is used. The MLP consists of three layers with 27 input neurons, 81 hidden layer neurons and two output neurons. Data from the Belle II's central drift chamber (CDC) is used for this task, since it is dedicated to the detection of particle tracks. Before being processed by the network, the raw detector data is first combined into a 2D track based on so-called track segments, which are groupings of adjacent active sense wires. The output of the NNT delivers the origin of the track in *z*, along the beampipe, as well as the polar angle θ. With the help of the z-vertex, the downstream global decision logic (GDL) can decide whether a track is from the interaction point or not. In addition, the particle momentum can be detected using the polar angle θ (Baehr et al., 2019).

The networks used in the NNT are trained offline. The first networks were trained with plain simulated data because no experimental data were available. For more recent networks, reconstructed tracks from the experimental data are used. For the training the iRPROP algorithm is used which is an extension of the RPROP backpropagation algorithm. Current results show a good correlation between the NNT tracks and reconstructed tracks. Since the event rate and the background noise are currently still tolerable, the z-cut, i.e., the allowed estimated origin of a track origin in order to be kept, is chosen at ±40 cm. With increasing luminosity and the associated increasing background, this z-cut can be tightened. Since the new Virtex Ultrascale based universal trigger board (UT4) is available for the NNT this year, an extension of the data preprocessing is planned. This will be done by a 3D Hough transformation for further efficiency increases. It has already been shown in simulation that a more accurate resolution and larger solid angle coverage can be achieved (Skambraks et al., 2020).

#### 2.2.2. Mu2e

*Context:* The Mu2e experiment at Fermilab (Bartoszek et al., 2014) will search for the charged lepton flavor violating process of neutrino-less μ → *e* coherent conversion in the field of an aluminum nucleus. About 7·10^17^ muons, provided by a dedicated muon beamline in construction at Fermilab, will be stopped in 3 years in the aluminum target. The corresponding single event sensitivity will be 2.5·10^−17^. To detect the signal *e*^−^ (*p* = 105 MeV), Mu2e uses a detector system made of a straw-tube tracker and a crystal electromagnetic calorimeter (Pezzullo, 2017).

*Challenges:* The trigger system is based on detector Read Out Controllers (ROCs) which stream out continuously the data, zero-suppressed, to the Data Transfer Controller units (DTCs). The proton pulses are delivered at a rate of about 600 kHz and a duty cycle of about 30% (0.4 s out of 1.4 s of the booster-ring delivery period). Each proton pulse is considered a single event, with the data from each event then grouped at a single server using a 10 Gbps Ethernet switch. Then, the online reconstruction of the events starts and makes a trigger decision. The trigger system needs to satisfy the following requirements: (1) provide efficiency better than 90% for the signals; (2) keep the trigger rate below a few kHz – equivalent to 7 Pb/year; (3) achieve a processing time < 5 ms/event. Our main physics triggers use the information of the reconstructed tracks to make the final decision.

*Existing and Planned Work:* The current strategy is to perform the helix pattern recognition and the track reconstruction with the CPUs of the DAQ servers, but so far this design showed limitations in matching the required timing performance (Pezzullo, 2020). Another idea that the collaboration started exploring is to perform the early stage of the track reconstruction on the ROC and DTC FPGA using the High Level Synthesis tool (HLS) and the hls4ml (Pierini et al., 2020) package. The Mu2e helix pattern-recognition algorithms (Pezzullo, 2020) are a natural fit for these tools for several reasons: they use neural-networks to clean up the recorded straw-hits from hits by low-momentum electrons (*p* < 10 MeV) and they perform large combinatorics calculations when reconstructing the helicoidal electron trajectory. This R&D is particularly important for the design of the trigger system of the planned upgrade of Mu2e (Abusalma et al., 2018), where we expect to: (i) increase the beam intensity by at least a factor of 10, (ii) increase the duty cycle to at least 90%, and (iii) increase the number of detector's channels to cope with the increased occupancy.

### 2.3. Materials Discovery

#### 2.3.1. Materials Synthesis

*Context:* Advances in electronics, transportation, healthcare, and buildings require the synthesis of materials with controlled synthesis-structure-property relationships. To achieve application-specific performance metrics, it is common to design and engineer materials with highly ordered structures. This directive has led to a boom in non-equilibrium materials synthesis techniques. Most exciting are additive synthesis and manufacturing techniques, for example, 3d-printing (Visser et al., 2015; Parekh et al., 2016; Zarek et al., 2016; Ligon et al., 2017; Wang et al., 2020c) and thin film deposition (Richter, 1990; Chrisey and Hubler, 1994; Kelly and Arnell, 2000; Yoshino et al., 2000; Park and Sudarshan, 2001; George, 2010; Marvel et al., 2013), where complex nanoscale architectures of materials can be fabricated. To glean insight into synthesis dynamics, there has been a trend to include *in situ* diagnostics to observe synthesis dynamics (Egelhoff and Jacob, 1989; Thomas, 1999; Langereis et al., 2007; Ojeda-G-P et al., 2017). There is less emphasis on automating the downstream analysis to turn data into actionable information that can detect anomalies in synthesis, guide experimentation, or enable closed-loop control. Part of the challenge with automating analysis pipelines for *in situ* diagnostics is the highly variable nature and multimodality of the measurements and the sensors. A system might measure many time-resolved state variables (time-series) at various locations (e.g., temperature, pressure, energy, flow rate, etc.) (Hansen et al., 1999). Additionally, it is common to measure time-resolved spectroscopic signals (spectrograms) that provide, for instance, information about the dynamics of the chemistry and energetic distributions of the materials being synthesized (Dauchot et al., 1995; Aubriet et al., 2002; Cooks and Yan, 2018; Termopoli et al., 2019). Furthermore, there are a growing number of techniques that leverage high-speed temporally-resolved imaging to observe synthesis dynamics (Trigub et al., 2017; Ojeda-G-P et al., 2018).

*Challenges:* Experimental synthesis tools and *in situ* diagnostic instrumentation are generally semi-custom instruments provided by commercial vendors. Many of these vendors rely on proprietary software to differentiate their products from their competition. In turn, the closed-nature of these tools and even data schemas makes it hard to utilize these tools fully. The varied nature and suppliers for sensors compounds this challenge. Integration and synchronization of multiple sensing modalities require a custom software solution. However, there is a catch-22 because the software does not yet exist. Researchers cannot be ensured that the development of analysis pipelines will contribute to their ultimate goal to discover new materials or synthesize materials with increased fecundity. Furthermore, there are significant workforce challenges as most curriculums emphasize Edisonian rather than computational methods in the design of synthesis. There is an urgent need for multilingual trainees fluent in typically disparate fields.

*Existing and Planned Work:* Recently, the materials science community has started to embrace machine learning to accelerate scientific discovery (Ramprasad et al., 2017; Butler et al., 2018; Schmidt et al., 2019). However, there have been growing pains. The ability to create highly overparameterized models to solve problems with limited data provides a false sense of efficacy without the generalization required for science. Machine learning model architectures designed for natural time-series and images are ill-posed for physical processes governed by equations. In this regard, there is a growing body of work to embed physics in machine learning models, which serve as the ultimate regularizers. For instance, rotational (Kalinin et al., 2020; Oxley et al., 2020) and Euclidean equivariance (Smidt, 2020; Smidt et al., 2021) has been built into the model architectures, and methods to learn sparse representations of underlying governing equations have been developed (Champion et al., 2019; de Silva et al., 2020; Kaheman et al., 2020).

Another challenge is that real systems have system-specific discrepancies that need to be compensated (Kaheman et al., 2019). For example, a precursor from a different batch might have a slightly different viscosity that needs to be considered. There is an urgent need to develop these foundational methods for materials synthesis. Complementing these foundational studies, there has been a growing body of literature emphasizing post-mortem machine-learning-based analysis of *in situ* spectroscopies (Trejo et al., 2019; Provence et al., 2020). As these concepts become more mature, there will be an increasing emphasis on codesign of synthesis systems, machine learning methods, and hardware for on-the-fly analysis and control. This effort toward self-driving laboratories is already underway in wet-chemical synthesis where there are minimal dynamics, and thus, latencies are not a factor (Langner et al., 2020; MacLeod et al., 2020). Future efforts will undoubtedly focus on controlling dynamic synthesis processes where millisecond-to-nanosecond latencies are required.

#### 2.3.2. Scanning Probe Microscopy

*Context:* Touch is the first sense humans develop. Since the atomic force microscope's (AFM) invention in 1985 (Binnig et al., 1986), humans have been able to “feel”İ surfaces with atomic level resolution with pN sensitivity. AFMs rely on bringing an atomically sharp tip mounted on a cantilever into contact with a surface. By scanning this tip nanometer-to-atomically resolved images can be constructed by measuring the angular deflection of a laser bounced off the cantilever. This detection mechanism provides high-precision sub-angstrom measures of displacement.

By adding functionality to the probe (e.g., electrical conductivity Benstetter et al., 2009, resistive heaters King, 2005, single-molecule probes Oberhauser et al., 2002, and N-V centers Ariyaratne et al., 2018), scanning probe microscopy (SPM) can measure nanoscale functional properties, including electrical conductivity (Gómez-Navarro et al., 2005; Seidel et al., 2010), piezoresponse (Jesse and Kalinin, 2011), electrochemical response (Jesse et al., 2012), magnetic force (Kazakova et al., 2019), magnetometry (Casola et al., 2018), and much more. These techniques have been expanded to include dynamics measurements during a tip-induced perturbation that drives a structural transformation. These methods have led to a boom in new AFM techniques, including fast-force microscopy (Benaglia et al., 2018), current-voltage spectroscopies (Holstad et al., 2020), band-excitation-based spectroscopies (Jesse et al., 2018), and full-acquisition mode spectroscopies (Somnath et al., 2015). What has emerged is a data deluge where these techniques are either underutilized or under-analyzed.

*Challenges:* The key practical challenge is that it takes on days-to-weeks to analyze data from a single measurement properly. As a result, experimentalists have little information on how to design their experiments. There is even minimal feedback on whether the experiments have artifacts (e.g., tip damage) that would render the results unusable. The number of costly failed experiments is a strong deterrent to conducting advanced scanning probe spectroscopies and developing even more sophisticated imaging techniques. There is a significant challenge in both the acceleration and automation of analysis pipelines.

*Existing and Planned Work:* In materials science, scanning probe microscopy has quickly adopted machine learning. Techniques for linear and nonlinear spectral unmixing provide rapid visualization and extraction of information from these datasets to discover and unravel physical mechanisms (Collins et al., 2020a,b; Ziatdinov et al., 2020; Kalinin et al., 2021). The ease of applying these techniques has led to justified concerns about the overinterpretation of results and overextension of linear models (Griffin et al., 2020) to highly nonlinear systems. More recently, long-short term memory autoencoders were controlled to have non-negative and sparse latent spaces for spectral unmixing. By traversing the learned latent space, it has been possible to draw complex structure-property relationships (Agar et al., 2019; Holstad et al., 2020). There are significant opportunities to accelerate the computational pipeline such that information can be extracted on practically relevant time scales by the experimentalist on the microscope.

Due to the high velocity of data, up to GB/s, with sample rates of 100,000 spectra, extracting even cursory information will require the confluence of data-driven models, physics-informed machine learning, and AI hardware. As a tangible example, in band-excitation piezoresponse force microscopy, the frequency-dependent cantilever response is measured at rates up to 2,000 spectra-per-second. Extracting the parameters from these measurements requires fitting the response to an empirical model. Using least-squares fitting throughput is limited to ~50-fits/core-minute, but neural networks provide an opportunity to accelerate analysis and better handle noisy data (Borodinov et al., 2019). There is an opportunity to deploy neural networks on GPU or FPGA hardware accelerators to approximate and accelerate this pipeline by orders of magnitude.

### 2.4. Fermilab Accelerator Controls

*Context:* The Fermi National Accelerator Laboratory (Fermilab) is dedicated to investigating matter, energy, space, and time (Fermilab, 2021). For over 50 years, Fermilab's primary tool for probing the most elementary nature of matter has been its vast accelerator complex. Spanning a number of miles of tunnels, the accelerator complex is actually multiple accelerators and beam transport lines each representing different accelerator techniques and eras of accelerator technologies. In its long history, Fermilab's accelerator complex has had to adapt to the mission, asking more of the accelerators than they were designed for and often for purposes they were never intended. This often resulted in layering new controls on top of existing antiquated hardware. Until recently, accelerator controls focused mainly on providing tools and data to the machine operators and experts for tuning and optimization. Having recognized the future inadequacies of the current control system and the promise of new technologies such as ML, the Fermilab accelerator control system will be largely overhauled in the coming years as part of the Accelerator Controls Operations Research Network (ACORN) project (Fermilab, 2021).

*Challenges:* The accelerator complex brings unique challenges for machine learning. Particle accelerators are immensely complicated machines, each consisting of many thousands of variable components and even larger data sources. Their large size and differing types, resolution, and frequency of data mean collecting and synchronizing data is difficult. Also, as one might imagine, control and regulation of beams that travel at near light speeds is always a challenge. Maintaining and upgrading the accelerator complex controls is costly. For this reason, much of the accelerator complex is a mixture of obsolete, new and cutting edge hardware.

*Existing and Planned Work:* Traditional accelerator controls have focused on grouping like elements so that particular aspects of the beam can be tuned independently. However, many elements are not always completely separable. Magnets, for example, often have higher-order fields that affect the beam in different ways than is the primary intent. Machine learning has made it finally possible to combine previously believed to be unrelated readings and beam control elements into new novel control and regulation schemes.

One such novel regulation project is underway for the Booster Gradient Magnet Power Supply (GMPS). GMPS controls the primary trajectory of the beam in the Booster (OPE, 2021). The project hopes to increase the regulation precision of GMPS ten-fold. When complete, GMPS would be the first FPGA online ML-model-based regulation system in the Fermilab accelerator complex (John et al., 2021). The promise of ML for accelerator controls is so apparent to the Department of Energy that a call for accelerator controls using ML was made to the national labs (DOE, 2020). Of the two proposals submitted by Fermilab and approved by the DOE is the Real-time Edge AI for Distributed Systems (READS) project. READS is actually two projects. The first READS project will create a complimentary ML regulation system for slow extraction from the Delivery Ring to the future Mu2e experiment (Bartoszek et al., 2015). The second READS project will tackle a long-standing problem with de-blending beam losses in the Main Injector (MI) enclosure. The MI enclosure houses two accelerators, the MI and the Recycler. During normal operation, high intensity beams exist in both machines. One to use ML to help regulate slow spill in the Delivery ring to Mu2e, and another to develop a real-time online model to de-blend losses coming from the Recycler and Main Injector accelerators which share an enclosure. Both READS projects will make use of FPGA online ML models for inference and will collect data at low latencies from distributed systems around the accelerator complex (Seiya et al., 2021).

### 2.5. Neutrino and Direct Dark Matter Experiments

#### 2.5.1. Accelerator Neutrino Experiments

*Context:* Accelerator neutrino experiments detect neutrinos with energies ranging from a few tens of MeV up to about 20 GeV. The detectors can be anywhere from tens of meters away from the neutrino production source, to as far as away as 1,500 km. For experiments with longer baselines it is common for experiments to consist of both a near (~1 km baseline) and a more distant far detector (100'skm baseline). Accelerator neutrino experiments focused on long-baseline oscillations use highly pure muon neutrino beams, produced by pion decays in flight. By using a system of magnetic horns it is possible to produce either a neutrino, or antineutrino beam. This ability is particularly useful for CP-violation measurements. Other experiments use pions decaying at rest, which produce both muon and electron flavors.

The primary research goal of many accelerator neutrino experiments is to perform neutrino oscillation measurements; the process by which neutrinos created in one flavor state are observed interacting as different flavor states after traveling a given distance. Often this takes the form of measuring electron neutrino appearance and muon neutrino disappearance. The rate of oscillation is energy-dependent, and so highly accurate energy estimation is essential. Another key research goal for accelerator neutrinos is to measure neutrino cross-sections, which in addition to accurate energy estimation requires the identification of the particles produced by the neutrino interaction.

*Challenges:* Accelerator neutrino experiments employ a variety of detector technologies. These range from scintillator detectors such as NOvA (Ayres et al., 2007) (liquid), MINOS (Ambats et al., 1998) (solid), and MINERvA (MIN, 2006) (solid), to water Cherenkov detectors such as T2K (Abe et al., 2011), and finally liquid argon time projection chambers such as MicroBooNE (Fleming, 2012), ICARUS (Amerio et al., 2004), and DUNE (Abi et al., 2020a). Pion decay-at-rest experiments (COHERENT Akimov et al., 2015, JSNS^2^ Ajimura et al., 2017) use yet different technologies (liquid and solid scintillators, as well as solid-state detectors). The individual challenges and solutions are unique to each experiment, though common themes do emerge.

Neutrino interactions are fairly uncommon due to their low cross-section. Some experiments can see as few as one neutrino interaction per day. This, combined with many detectors being close to the surface, means that analyses have to be highly efficient whilst achieving excellent background rejection. This is true both in online data taking and offline data analysis.

As experiments typically have very good temporal and/or spatial resolution it is often fairly trivial to isolate entire neutrino interactions. This means that it is then possible to use image recognition tools such as CNNs to perform classification tasks. As a result, many experiments initially utilized variants of GoogLeNet, though many are now transitioning to use GNNs and networks better able to identify sparse images.

*Existing and Planned Work:* As discussed in Section 2.5.2, DUNE will use machine learning in its triggering framework to handle its immense data rates and to identify candidate interactions, for both traditional neutrino oscillation measurements and for candidate solar and supernova events. Accelerator neutrino experiments have successfully implemented machine learning techniques for a number of years, the first such example being in 2017 (Adamson et al., 2017), where the network increased the effective exposure of the analysis by 30%. Networks aimed at performing event classification are common across many experiments, with DUNE having recently published a network capable of exceeding its design sensitivity on simulated data and which includes outputs that count the numbers of final state particles from the interaction (Abi et al., 2020a).

Experiments are becoming increasingly cognizant of the dangers of networks learning features of the training data beyond what is intended. For this reason, it is essential to carefully construct training datasets such that this risk is reduced. However, it is not possible to correct or quantify bias which is not yet known; therefore the MINERvA experiment has explored the use of a domain adversarial neural network (Perdue et al., 2018) to reduce unknown biases from differences in simulated and real data. The network features a gradient reversal layer in the domain network (trained on data), thus discouraging the classification network (trained on simulation) to learn from any features that behave differently between the two domains. A more robust exploration of the machine learning applied to accelerator neutrino experiments can be found here in Psihas et al. (2020).

#### 2.5.2. Neutrino Astrophysics

*Context:* Neutrino astrophysics spans a wide range of energies, with neutrinos emitted from both steady-state and transient sources with energies from less than MeV to EeV scale. Observations of astrophysical neutrinos are valuable both for the understanding of neutrino sources and for probing fundamental physics. Neutrino detectors designed for observing these tend to be huge scale (kilotons to megatons). Existing detectors involve a diverse range of materials and technologies for particle detection; they include Cherenkov radiation detectors in water and ice, liquid scintillator detectors and, liquid argon time projection chambers.

Astrophysical neutrinos are one kind of messenger contributing to the thriving field of *multimessenger astronomy*, in which signals from neutrinos, charged particles, gravitational waves, and photons spanning the electromagnetic spectrum are observed in coincidence. This field has had some recent spectacular successes (Abbott et al., 2017a; Aartsen et al., 2018; Graham et al., 2020). For multimessenger transient astronomy, time is of the essence for sharing data and locating sources. *Directional information* from the neutrinos is critically valuable, to allow prompt location of the source by other messengers.

Potential interesting transient astrophysical sources include sources of ultra-high energy neutrinos, as well as nearby stellar core collapses. Neutrinos in the multi-GeV and higher range are emitted from distant cosmic sources, including kilonovae and blazars, and cubic-km-scale water-based Cherenkov detectors such as IceCube at the South Pole can produce fast alerts from single neutrino observations.

Core-collapse supernovae are another promising use case for fast machine learning. These are copious sources of few tens of MeV-scale neutrinos, which are emitted in a burst lasting a few tens of seconds (Scholberg, 2012; Mirizzi et al., 2016). The neutrinos are prompt after core collapse (as will be gravitational waves) but observable electromagnetic radiation will not emerge for anywhere from tens to 10^6^s, depending on the nature of the progenitor and its envelope (Kistler et al., 2013). Low-latency information is therefore immensely valuable. Core-collapse supernovae are rare events within the distance range observable by current and near-future neutrino detectors. They occur only every several decades, which makes prompt and robust detection especially important. The SuperNova Early Warning System (Antonioli et al., 2004; Al Kharusi et al., 2020) aims to provide a prompt alert from a coincidence of burst detections. However, pointing information from neutrinos is relatively difficult to extract promptly. Detectors with the capability for prompt pointing thanks to the anisotropy of neutrino interactions (i.e., the interaction products that remember where the neutrino came from) offer the best prospects, but these need to be able to select neutrino events from background and reconstruct their directions with very low latency.

Presupernova neutrinos are another interesting possibility. In the final stages of stellar burning, one expects a characteristic uptick in neutrino luminosity and average energy, producing observable events in detectors for nearby progenitors. This could give a warning of hours or perhaps days before core collapse for the nearest progenitors. For this case, fast selection of neutrino-like events and reconstruction of their directional information for background reduction is needed.

*Challenges:* The challenges, in general, are fast selection and reconstruction of neutrino event (interaction) information. The specifics of the problem depend on the particular detector technology, but in general, the charged particle products of a neutrino interaction will have a distinctive topology or other signature and must be selected from a background of cosmic rays, radiologicals, or detector noise. Taking as an example a liquid argon time projection chamber like the Deep Underground Neutrino Experiment (DUNE), neutrino-induced charged particles produce charge and light signals in liquid argon. Supernova neutrino interactions appear as small (tens of cm spatial scale) stubs and blips (Abi, 2020; Abi et al., 2020b). The recorded neutrino event information from the burst can be used to reconstruct the supernova direction to ~5–10° for core collapse at 10kpc distance (Abi, 2020; Roeth, A. J., 2020). The neutrino events need to be selected from a background of radioactivity and cosmogenics, as well as detector noise, requiring background reduction of many orders of magnitude. Total data rate amounts to ~40Tb/s. The detector must take data for a decade or more at this rate, with near-continuous uptime.

For steady-state signals such as solar neutrinos, triggering on individual events in the presence of large backgrounds is a challenge that can be addressed with machine learning. For burst signals, the triggering is a different problem: the general strategy is to read out all information on every channel within a tens-of-seconds time window, for the case of a triggered burst. This leads to the subsequent problem of sifting the signal events and reconstructing sufficient information on a very short timescale to point back to the supernova. The required timescale is minutes, or preferably seconds. Both the event-by-event triggering and fast directional reconstruction can be addressed with fast machine learning.

*Existing and Planned Work:* There are a number of existing efforts toward the use of machine learning for particle reconstruction in neutrino detectors including water Cherenkov, scintillator, and liquid argon detectors. These overlap to some extent with the efforts described in Section 2.5.1. Efforts directed specifically toward real-time event selection and reconstruction are ramping up. Some examples of ongoing efforts can be found in Abi et al. (2020a), Acciarri et al. (2020), Psihas et al. (2020), Abratenko et al. (2020), Wang et al. (2020a), Drielsma et al. (2021), and Qian et al. (2021).

#### 2.5.3. Direct Detection Dark Matter Experiments

*Context:* Direct dark matter (DM) search experiments take advantage of the vastly abundant DM in the universe and are searching for direct interactions of DM particles with the detector target material. The various target materials can be separated into two main categories, crystals and liquid noble gases, though other material types are subject to ongoing detector R&D efforts (Alexander et al., 2016; Schumann, 2019).

One of the most prominent particle DM candidates is the WIMP (weakly interacting massive particle), a thermal, cold DM candidate with an expected mass and coupling to Standard Model particles at the weak scale (Jungman et al., 1996). However, decades of intensive searches both at direct DM and at collider experiments have not yet been able to discover^2^ the vanilla WIMP while excluding most of the parameter space of the simplest WIMP hypothesis (Schumann, 2019). This instance has lead to a shift in paradigm for thermal DM toward increasingly lower masses well below 1GeV (and thus the weak scale) (Boehm and Fayet, 2004) and as low as a few keV, i.e., the warm DM limit (Weinberg et al., 2015). Thermal sub-GeV DM is also referred to as light dark matter (LDM). Other DM candidates that are being considered include non-thermal, bosonic candidates like dark photons, axions and axion-light particles (ALPs) (Holdom, 1986; Svrcek and Witten, 2006; Peccei, 2008).

The most common interactions direct DM experiments are trying to observe are thermal DM scattering off either a nucleus or an electron and the absorption of dark bosons under the emission of an electron. The corresponding signatures are either nuclear recoil or electron recoil signatures.

*Challenges:* In all mentioned interactions, and independent of the target material, a lower DM mass means a smaller energy deposition in the detector and thus a signal amplitude closer to the baseline noise. Typically, the baseline noise has non-Gaussian contributions that can fire a simple amplitude-over-threshold trigger even if the duration of the amplitude above threshold is taken into account. The closer the trigger threshold is to the baseline, the higher the rate of these spurious events. In experiments which cannot read out raw data continuously and which have constraints on the data throughput, the hardware-level trigger threshold has thus to be high enough to significantly suppress accidental noise triggers.

In the hunt for increasingly lower DM masses, however, an as-low-as-possible trigger threshold is highly desirable, calling for a more sophisticated and extremely efficient event classification at the hardware trigger level. Particle-induced events have a known, and generally constant, pulse-shape while non-physical noise “events" (e.g., induced by the electronics) generally have a varying pulse-shape which is not necessarily predictable. A promising approach in such a scenario is the use of machine learning techniques for most efficient noise event rejection in real-time allowing to lower the hardware-level trigger threshold, and thus the low mass reach in most to all direct DM searches, while remaining within the raw data read-out limitations imposed by the experimental set-up.

*Existing and Planned Work:* Machine learning is already applied by various direct DM search experiments (Simola et al., 2019; Khosa et al., 2020; Szydagis et al., 2021), especially in the context of offline data analyses. However, it is not yet used to its full potential within the direct DM search community. Activities in this regard are still ramping up but with increasing interest, efforts, and commitment. Typical offline applications to date are the reconstruction of the energy or position of an event and the classification of events (e.g., signal against noise or single-scattering against multiple-scattering). In parallel R&D has started on real-time event classification within the FPGA-level trigger architecture of the SuperCDMS experiment (Agnese et al., 2017) with the long-term goal of lowering the trigger threshold notably closer to the baseline noise without triggering on spurious events. While these efforts are being conducted within the context of SuperCDMS the goal is a modular trigger solution for easier adaption to other experiments.

### 2.6. Electron-Ion Collider

*Context:* The Electron-Ion Collider (EIC) will support the exploration of nuclear physics over a wide range of center-of-mass energies and ion species, using highly-polarized electrons to probe highly-polarized light ions and unpolarized heavy ions. The frontier accelerator facility will be designed and constructed in the U.S. over the next 10 years. The requirements of the EIC are detailed in a white paper (Accardi et al., 2016), the 2015 Nuclear Physics Long Range Plan (Aprahamian et al., 2015), and an assessment of the science by the National Academies of Science (National Academies of Sciences Engineering and Medicine, 2018). The EIC's high luminosity and highly polarized beams will push the frontiers of particle accelerator science and technology and will enable us to embark on a precision study of the nucleon and the nucleus at the scale of sea quarks and gluons, over all of the kinematic range that is relevant as described in the EIC Yellow Report (Abdul Khalek et al., 2021).

*Challenges:* While the event reconstruction at the EIC is likely easier than the same task at present LHC or RHIC hadron machines, and much easier than for the High-Luminosity LHC, which will start operating 2 years earlier than the EIC, possible contributions from machine backgrounds form a challenge. The expected gain in CPU performance in the next 10 years as well as the possible improvement in the reconstruction software from the use of AI and ML techniques give a considerable margin to cope with higher event complexity that may come by higher background rates. Software design and development will constitute an important ingredient for the future success of the experimental program at the EIC. Moreover, the cost of the IT related components, from software development to storage systems and to distributed complex e-Infrastructures can be raised considerably if a proper understanding and planning is not taken into account from the beginning in the design of the EIC. The planning must include AI and ML techniques, in particular for the compute-detector integration at the EIC, and training in these techniques.

*Existing and Planned Work:* Accessing the EIC physics of interest requires an unprecedented integration of the interaction region (IR) and detector designs. The triggerless DAQ scheme that is foreseen for the EIC will extend the highly integrated IR-detector designs to analysis. A seamless data processing from DAQ to analysis at the EIC would allow to streamline workflows, e.g., in a combined software effort for the DAQ, online, and offline analysis, as well as to utilize emerging software technologies, in particular fast ML algorithms, at all levels of data processing. This will provide an opportunity to further optimize the physics reach of the EIC. The status and prospects for “AI for Nuclear Physics” have been discussed in a workshop in 2020 (Bedaque et al., 2021). Topics related to fast ML are intelligent decisions about data storage and (near) real-time analysis. Intelligent decisions about data storage are required to ensure the relevant physics is captured. Fast ML algorithms can improve the data taken through data compactification, sophisticated triggers, and fast online analysis. At the EIC, this could include automated alignment and calibration of the detectors as well as automated data-quality monitoring. A (near) real-time analysis and feedback enables quick diagnostics and optimization of experimental setups as well as significantly faster access to physics results.

### 2.7. Gravitational Waves

*Context:* As predicted by Einstein in 1916, gravitational waves are fluctuations in the gravitational field which within the theory of general relativity manifest as a change in the spacetime metric. These ripples in the fabric of spacetime travel at the speed of light and are generated by changes in the mass quadruple moment, as, for example, in the case of two merging black holes (Abbott et al., 2016b). To detect gravitational waves, the LIGO/Virgo/KAGRA collaborations employ a network of kilometer-scale laser interferometers (Harry and LIGO Scientific Collaboration, 2010; Aso et al., 2013; Acernese et al., 2014; Affeldt et al., 2014). An interferometer consists of two perpendicular arms; as the gravitational wave passes through the instrument, it stretches one arm while compressing the other in an alternating pattern dictated by the gravitational wave itself. Such length difference is then measured from the laser interference pattern.

Gravitational waves are providing a unique way to study fundamental physics, including testing the theory of general relativity at the strong field regime, the speed of propagation and polarization of gravitational waves, the state of matter at nuclear densities, formation of black holes, effects of quantum gravity and more. They have also opened up a completely new window for observing the Universe and in a complementary way to one enabled by electromagnetic and neutrino astronomy. This includes the study of populations, including their formation and evolution, of compact objects such as binary black holes and neutron stars, establish the origin of gamma-ray bursts (GRBs), measure the expansion of the Universe independently of electromagnetic observations, and more (Abbott et al., 2017b).

*Challenges:* In the next observing run in 2022, LIGO, Virgo, and KAGRA will detect an increasing number of gravitational-wave candidates. This poses a computational challenge to the current detection framework, which relies on matched-filtering techniques that match parameterized waveforms (templates) from simulations into the gravitational-wave time series data (Sathyaprakash and Dhurandhar, 1991; Vaseghi, 2001; Abbott et al., 2016b). Matched filtering scales poorly as the low-frequency sensitivity of the instrument improves and the search parameter space of the gravitational wave expands to cover spin effects and low mass compact objects. To estimate the physical properties of the gravitational wave, stochastic Bayesian posterior samplers, such as Markov-chain Monte Carlo and Nested Sampling, have been used until now. Such analysis approaches can take up hours to days to complete (Abbott et al., 2016a). The latency introduced by the current search and parameter estimation pipeline is non-negligible and can hinder electromagnetic follow-ups of time-sensitive sources like binary neutron stars, supernovae, and other, yet unknown, systems.

Observations of gravitational-wave transients are also susceptible to environmental and instrumental noise. Transient noise artifacts can be misidentified as a potential source, especially when the gravitational-wave transients have an unknown morphology (e.g., supernovae, neutron star glitches). Line noise in the noise spectrum of the instruments can affect the search for continuous gravitational waves (e.g., spinning neutron stars) and stochastic gravitational waves (e.g., astrophysical background of gravitational waves from unresolved compact binary systems). These noise sources are difficult to simulate, and current noise subtraction techniques are insufficient to remove the more complex noise sources, such as non-linear and non-stationary ones.

*Existing and Planned Work:* In recent years, machine learning algorithms have been explored in different areas of gravitational-wave physics (Cuoco et al., 2020). CNNs have been applied to detect and categorize compact binary coalescence gravitational waves (Kim et al., 2015, 2020; Gabbard et al., 2018; George and Huerta, 2018; Gebhard et al., 2019), burst gravitational waves from core-collapse supernovae (Astone et al., 2018; Chan et al., 2020; Iess et al., 2020), and continuous gravitational waves (Dreissigacker et al., 2019; Beheshtipour and Papa, 2020). Besides, recurrent neural networks (RNNs) based autoencoders have been explored to detect gravitational wave using an unsupervised strategy (Moreno et al., 2021). FPGA-based RNNs are also explored to show the potential in low-latency detection of gravitational wave (Que et al., 2021). Applications of ML in searches of other types of gravitational waves, such as generic burst and stochastic background, are currently being explored. Moreover, probabilistic and generative ML models can be used for posterior sampling in gravitational-wave parameter estimation and achieve comparable performance to Bayesian sampler on mock data while taking significantly less time to complete (Shen et al., 2019; Chua and Vallisneri, 2020; Gabbard et al., 2020). ML algorithms are also being used to improve the gravitational-wave data quality and subtract noise. Transient noise artifacts can be identified and categorized from their time-frequency transforms and constant-Q transforms (Zevin et al., 2017; Razzano and Cuoco, 2018) or through examining hundreds of thousands of LIGO's auxiliary channels (Biswas et al., 2013). These auxiliary channels can also be used to subtract quasi-periodic noise sources (e.g., spectral lines) (Ormiston et al., 2020; Vajente et al., 2020). Although ML algorithms have shown a lot of promise in gravitational-wave data analysis, many of these algorithms are still at the proof-of-concept stage and have not yet been successfully applied in real-time analysis. Current efforts seek to create a computational infrastructure for low-latency analysis, improve the quality of the training data (e.g., expanding the parameter space, using a more realistic noise model), and better quantify the performance of these algorithms on longer stretches of data.

### 2.8. Biomedical Engineering

*Context:* We have seen an explosion of biomedical data, such as biomedical images, genomic sequences, and protein structures, due to the advances in high-resolution and high-throughput biomedical devices. AI-augmented reality-based microscopy (Chen et al., 2019) enables automatic analysis of cellular images and real-time characterization of cells. Machine learning is used *in-silico* prediction of fluorescent labels, label-free rare cell classification, morphology characterization, and RNA sequencing (Christiansen et al., 2018; Tang et al., 2018; Li et al., 2020a; Siu et al., 2020; Wang et al., 2020b). For *in-situ* cell sorting, real-time therapy response prediction, and augmented reality microscope-assisted diagnosis (Nitta et al., 2018; Chen et al., 2019; Sakellaropoulos et al., 2019), it is important to standardize and optimize data structure in deep learning models to increase speed and efficiency. Various machine-learning-based algorithms for detecting hemorrhage and lesions, accelerating diagnosis, and enhancing medical video and image quality have also been proposed in biopsy analysis and surgery assistance.

*Challenges:* A major challenge for clinical application of ML is inadequate training and testing data. The medical data annotation process is both time-consuming and expensive for large image and video datasets which require expert knowledge. The latency of trained models' inference also introduces computational difficulties in performing real-time diagnosis and surgical operation. The quality of services for time-critical healthcare requires less than 300 ms as real-time video communication (Shukla et al., 2019). For reaching 60 frames per second (FPS) high-quality medical video, the efficiency and performance of a deep learning model become crucial.

*Existing and Planned Work:* Many changes in ML algorithms have involved improvements to performance both in accuracy and inference speed. Some state-of-art machine learning models can reach a high speed for inference. For example, *YOLOv3-tiny* (Adarsh et al., 2020), an object detection model commonly used for medical imaging, can process images at over 200 FPS on a standard dataset with producing reasonable accuracy. Currently both GPU- and FPGA-based (Chang and Sheu, 2020; Satpathy et al., 2020; Zhang et al., 2020a), distributed networks of wireless sensors connected to cloud ML (edge computing), and 5G-high-speed-WiFi-based ML models are deployed in medical AI applications (Chen et al., 2018; Morocho-Cayamcela et al., 2019; Zhang et al., 2020c). ML models for fast diagnosis of stroke, thrombosis, colon polyps, cancer, and epilepsy have significantly reduced the time in lesion detection and clinical decision (Bagheri et al., 2019; Horie et al., 2019; Lee et al., 2020; Nafee et al., 2020; Nogueira-Rodríguez et al., 2020). Real-time AI-assisted surgery can improve perioperative workflow, perform video segmentation (Volkov et al., 2017), detection of surgical instruments (Choi et al., 2017a), and visualization of tissue deformation (Tonutti et al., 2017). High-speed ML is playing a critical role in digital health, i.e.,, remote diagnosis, surgery, and monitoring (Zhang et al., 2020c).

### 2.9. Health Monitoring

*Context:* Our habits and behaviors affect our health and wellness. Unhealthy behaviors such as smoking, consuming excessive alcohol, or medication non-adherence often has an adverse effect on our health (Klesges et al., 1989; Baker et al., 2000; Sokol et al., 2005; White and Hingson, 2013). Traditional behavior monitoring approaches relied on self-reports, which were often biased and required intense manual labor (Althubaiti, 2016). With the advent of mobile and wearable devices, it is gradually becoming possible to monitor various human behaviors automatically and unobtrusively. Over the years, researchers have either developed custom wearable hardware or have used off-the-shelf commercial devices for mobile and wearable health (mHealth) monitoring (Ali et al., 2012; Dong et al., 2012; Parate et al., 2014; Bi et al., 2018; Mishra et al., 2020; Sen et al., 2020; Zhang et al., 2020b). The automatic and unobtrusive monitoring capability of these devices makes it possible to detect, identify and monitor behaviors, including unhealthy behaviors in a free-living setting.

*Challenges:* There are various challenges associated with monitoring habits and behaviors using wearable devices. Firstly, these devices should be capable of monitoring unhealthy behaviors accurately, and in real-time. The occurrence of these unhealthy behaviors in a free-living setting is often sparse as compared to other behaviors and thus it is important to spot them accurately, whenever they occur. Most existing systems take an offline ML approach of detecting these unhealthy behaviors, where the ML algorithm identifies these behaviors well after they have occurred. An offline approach prevents providing interventions that can minimize unhealthy behaviors. Thus, it is necessary to develop ML approaches that can detect these behaviors online, and in real-time, so that interventions such as just-in-time adaptive interventions (JITAIs) can be delivered. Secondly, since these devices capture sensitive information, it is necessary to ensure that an individual's privacy is preserved. Privacy-preserving approaches such as locally processing the data on-device can be taken so that critical information does not leave the device. Other approaches, such as collaborative learning, aim to increase speed while preserving data privacy (Idé et al., 2019). Finally, these behaviors can occur in various heterogeneous environments and thus the health monitoring system should be agnostic to where the behavior occurs. Such monitoring requires developing multiple machine learning models for diverse environments.

*Existing and Planned Work:* While existing work has ventured in various directions, there is a growing need for sensing health biomarkers correctly and developing ML approaches that are fast and can accurately identify these biomarkers. Researchers have focused on developing novel sensing systems that can sense various health behaviors and biomarkers (Holz and Wang, 2017; Bui et al., 2019; Bedri et al., 2020; Chun et al., 2020; Echterhoff and Wang, 2020; Li et al., 2020b; Pham et al., 2020). Historically, most of these novel sensing techniques were tested in controlled settings, but more recently researchers are ensuring that these systems can work seamlessly in free-living settings as well. This often requires developing multiple ML models, each catering to a specific context and environment. A new trend in this field has started relying on implementing models that can be implemented on-device and are both quick and accurate in detecting these behaviors. In addition to providing real-time interventions (Thomas and Bond, 2015; Nahum-Shani et al., 2018), on-device monitoring of these behaviors can reduce privacy concerns (Sadek et al., 2019). However, since wearable devices themselves might not be capable of processing the data, federated machine learning approaches are also being explored recently by several researchers (Rieke et al., 2020).

### 2.10. Cosmology

*Context:* Cosmology is the study of the Universe's origin (big bang), evolution, and future (ultimate fate). The large-scale dynamics of the universe are governed by gravity, where dark matter plays an important role, and the accelerating expansion rate of the universe itself, caused by the so-called dark energy. A non-exhaustive list of cosmological probes includes type Ia supernovae (Riess et al., 1998; Perlmutter et al., 1999; Betoule et al., 2014; Scolnic et al., 2018; Abbott et al., 2019b), cosmic microwave background (Fixsen et al., 1996; Spergel et al., 2003; Komatsu et al., 2011; Planck Collaboration et al., 2016, 2020), large-scale structures (including baryon acoustic oscillation) (Eisenstein et al., 2005; Percival et al., 2010; Delubac et al., 2015; Abbott et al., 2019a), gravitational lensing (Bacon et al., 2000, 2003; Collett and Auger, 2014; Suyu et al., 2017; Heymans et al., 2020) and 21 cm cosmology (McQuinn et al., 2007; Pritchard and Loeb, 2012; Maartens et al., 2015; Beardsley et al., 2016).

*Challenges:* As astronomy is approaching the big data era with next-generation facilities, such as the Nancy Grace Roman Space telescope (Sanderson et al., 2019), Vera C. Rubin Observatory (Ivezić et al., 2019), and Euclid telescope (Amiaux et al., 2012), the uncertainty budget in the estimation of cosmological parameters is no longer expected to be dominated by statistical uncertainties, but rather by systematic ones; understanding such uncertainties can lead to attaining sub-percent precision. On the other hand, the immense stream of astronomical images will be impossible to analyze in a standard fashion (by human interaction); new automated methods are needed to extract valuable pieces of cosmological data.

*Existing and Future Work:* Current efforts are focused on applying ML techniques to study the influence of systematic biases on available analysis methods (e.g., for purposes of fitting or modeling) or on developing new methods to overcome present limitations; for example CNNs can be adapted to spherical surfaces to generate more accurate models when producing weak lensing maps (Perraudin et al., 2019), or to remove noise from cosmic microwave background maps (Petroff et al., 2020). In addition, discovery and classification engines are being developed to extract useful cosmological data from next-generation facilities (Narayan et al., 2018; Mahabal et al., 2019; Förster et al., 2020; Möller et al., 2020). Furthermore, ML is also being used in cosmological simulations to test new analyses and methods and to set the foundations for the first operation of such new facilities (Kamdar et al., 2016; Rodríguez et al., 2018; Villaescusa-Navarro et al., 2020). An extensive list of published ML applications in cosmology can be found in Stein (2020).

### 2.11. Plasma Physics

*Context:* The focus of this description is on the Plasma Physics/Fusion Energy Science domain with regard to the major system constraints encountered for existing and expected algorithms and data representations when dealing with the challenge of delivering accelerated progress in AI—enabled deep machine learning prediction and control of magnetically-confined thermonuclear plasmas. Associated techniques have enabled new avenues of data-driven discovery in the quest to deliver fusion energy—identified by the 2015 CNN “Moonshots for the twenty-first Century” televised series as one of 5 prominent grand challenges for the world today.

*Challenges:* An especially time-urgent and challenging problem is the need to reliably predict and avoid large-scale major disruptions in “tokamak systems” such as the EUROFUSION Joint European Torus (JET) today and the burning plasma ITER device in the near future—a ground-breaking $25B international burning plasma experiment with the potential capability to exceed “breakeven” fusion power by a factor of 10 or more with “first plasma”İ targeted for 2026 in France. The associated requirement is for real-time plasma forecasting with control capabilities operative during the temporal evolution of the plasma state well before the arrival of damaging disruptive events. High-level supervisory control of many lower-level control loops via actuators (analogous to advanced robotics operations) will be essential for ITER and future burning plasmas to protect the facility and to avoid operational limits (for magnets, walls, plasma position, stability, etc.) while optimizing performance.

*Existing and Planned Work:* In short, an overarching goal here involves developing realistic *predictive plasma models of disruptions integrated with a modern plasma control system to deliver the capability to design experiments before they are performed*. The associated novel AI-enabled integrated modeling tool would clearly be of great value for the most efficient and safe planning of the expensive discharges in ITER and future burning plasmas. Verification, validation, and uncertainty quantification of associated components would include: (1) development of predictive neural net models of the plasma and actuators that can be extrapolated to burning plasma scales via advanced Bayesian reinforcement learning methods that incorporate prior information into efficient inference algorithms; (2) systematic well-diagnosed experimental validation studies of components in the integrated plasma forecasting models involving massive amounts of data from major tokamak experiments worldwide (e.g., DIII-D in the US, KSTAR & EAST in Asia, JET in Europe, followed by JT60 SA—the large superconducting device in Japan that will precede ITER). This would ideally lead to a mature AI-enabled comprehensive control system for ITER and future reactors that feature integration with full pilot-plant system models.

At present, a key challenge is to deliver significantly improved methods of prediction with better than 95% predictive accuracy to provide advanced warning for disruption avoidance/mitigation strategies to be effectively applied before critical damage can be done to ITER. Significant advances in the deployment of deep learning recurrent and CNNs are well illustrated in Princeton's Deep Learning Code—“FRNN”—that have enabled the rapid analysis of large complex datasets on supercomputing systems. Associated acceleration of progress in predicting tokamak disruptions with unprecedented accuracy and speed is described in Kates-Harbeck et al. (2019). Included in this paper (and extensive references cited therein) are descriptions of FES data representation for physics features (density, temperature, current, radiation, fluctuations, etc.) and the nature of key plasma experiments featuring detectors/diagnostics with frame (event-based) level of accuracy accounting for required “zero-D” (scalar) and higher-dimension signals and real-time resolution recorded at manageable data rates. Rough future estimates indicate that ITER will likely require dealing with the challenge of processing and interpreting exabytes of complex spatial and temporal data.

Since simulation is another vital aspect of ITER data analysis, dealing with the associated major computational expenses will demand the introduction of advanced compressional methods. More generally, real-time predictions based on actual first-principles simulations are important for providing insights into instability properties and particle-phase space dynamics. This motivates the development of an AI-based “surrogate model”—for example, of the well-established HPC “gyrokinetic” particle-in-cell simulation code GTC (Lin et al., 1998) that would be capable of accurately simulating plasma instabilities in real-time. Data preparation and training a surrogate model—e.g., “SGTC”—provides a clear example of the modern task of integration/connection between modern High Performance Computing (HPC) predictive simulations with AI-enabled Deep Learning/Machine Learning campaigns. These considerations also serve to further illustrate/motivate the need to integrate HPC and Big Data ML approaches to expedite the delivery of scientific discovery.

As a final note, the cited paper (Kates-Harbeck et al., 2019) represents the first adaptable predictive DL software trained on leadership class supercomputing systems to deliver accurate predictions for disruptions across different tokamak devices (DIII-D in the US and JET in the UK). It features the unique statistical capability to carry out efficient “transfer learning” via training on a large database from one experiment (i.e., DIII-D) and be able to accurately predict disruption onset on an unseen device (i.e., JET). In more recent advances, the FRNN inference engine has been deployed in a real-time plasma control system on the DIII-D tokamak facility in San Diego, CA. As illustrated in slides 18 through 20 of the attached invited presentation slide deck, this opens up exciting avenues for moving from passive disruption prediction to active real-time control with subsequent optimization for reactor scenarios.

### 2.12. ML for Wireless Networking and Edge Computing

*Context:* Wireless devices and services have become a crucial tool for collecting and relaying big data in many scientific studies. Moreover, mobility information has proven to be extremely useful in understanding human activities and their impact on the environment and public health. The exponential growth of data traffic is placing significant pressure on the wireless infrastructure. In particular, inter-cell interference causes large variability in reliability and latency. To meet user demands for data communication and value-added AI/ML services, wireless providers must 1) develop more intelligent learning algorithms for radio resource management that adapt to complicated and ever-changing traffic and interference conditions; and 2) realize many ML/AI computations and functionalities in edge devices to achieve lower latency and higher communication efficiency.

*Challenges:* Conventional implementations of ML models, especially deep learning algorithms, lag far behind the packet-level dynamics for utility. Moreover, existing ML/AI services are often performed in the cloud for efficiency at the expense of communication overhead and higher latency. A major challenge in the wireless networking and edge computing context is to build a computing platform that can execute complex ML models at relevant timescales (< 10 ms) within small cell access points.

*Existing and Planned Work:* Researchers have proposed a variety of learning algorithms to perform specific radio resource management tasks using artificial neural networks (Calabrese et al., 2018; Challita et al., 2018; Huang et al., 2020; Zhu et al., 2020). Some of the first proposals to train a NN to perform transmit power control adopts supervised learning (Sun et al., 2018; Liang et al., 2020). More recent proposals adopt deep reinforcement learning approaches that work better with channel and network uncertainties and require little training data *a priori* (Liang et al., 2019; Zhao et al., 2019b; Meng et al., 2020; Nasir and Guo, 2020). A number of works are focused on the convergence of edge computing and deep learning (Chen and Ran, 2019; Zhang et al., 2019a; Wang et al., 2020d). A specific set of work is on federated learning where participants jointly train their models in lieu of sending all their data to a central controller for training purposes (Amiri and Gündüz, 2020; Niknam et al., 2020; Ren et al., 2020; Chen et al., 2021). All of the preceding work basically ends at the simulation stage for the lack of practical ML/AI solutions that are fast and computationally efficient at the same time. More specifically, the research challenge is to develop a computing platform that can execute complex ML models at a very fast timescale (< 10 ms) and can also be equipped in small cell access points. One project with a potentially very high impact is to map intelligent radio resource management algorithms (such as that of Nasir and Guo, 2020) onto an FPGA device suitable for deployment in a large network of connected and interfering access points. Another interesting project is to build a federated learning system to conduct time-sensitive ML for Internet-of-Things (IoT) devices where transferring data to centralized computing facilities is latency-prohibitive. This opens up entirely new possibilities for low-cost closed-loop IoT devices in healthcare, smart buildings, agriculture, and transportation.

## 3. Key Areas of Overlap

Real-time, accelerated AI inference show promises in improving the discovery potential at current and planned scientific instruments across the domains as detailed in Section 2. Design of high performant specialty systems for real-time/accelerated AI applications requires particular attention to the figure-of-merit of the target domain's ML algorithm. It might be dominated by its latency per inference, computational cost (e.g., power consumption), reliability, security, and ability to operate in extreme environments (e.g., radiation). For instance, ML might need to: trigger acquisition systems for rare events with ~100 ns latency on the Large Hadron Collider (Duarte et al., 2018); analyze multi-channel ambulatory health monitors at kilohertz frequencies where wireless transfer of data is not possible due to power limitations (~50 iPhone batteries/day for data transfer) or security requirements; or to keep pace with materials spectroscopy data streams on the order of terabits per second (Hart et al., 2017). Furthermore, real-time analysis of advanced scientific instrumentation must have an uninterrupted allocation of computing resources and patient sensitive information processed by wireless health devices must be secured. Such features and characteristics create quantifiable guidelines for understanding distinctions and commonalities among domains and applications. Thereby, we can coordinate efforts toward creating fundamental design principles and tools, which may address needs across seemingly disparate domains. Appropriate data representation is an essential first step of the design process as it determines the choice of NN architecture to be implemented in real-time systems that need to meet the performance targets outlined above. Prominent data representations of different scientific instruments are summarized below. Other areas of overlap across domains such as NN and hardware co-design tools and workflows, NN complexity reduction with quantization and pruning are also recent technology advancements in real-time/accelerated AI and therefore are outlined in Section 4.

### 3.1. Data Representations

**Figure 3:**
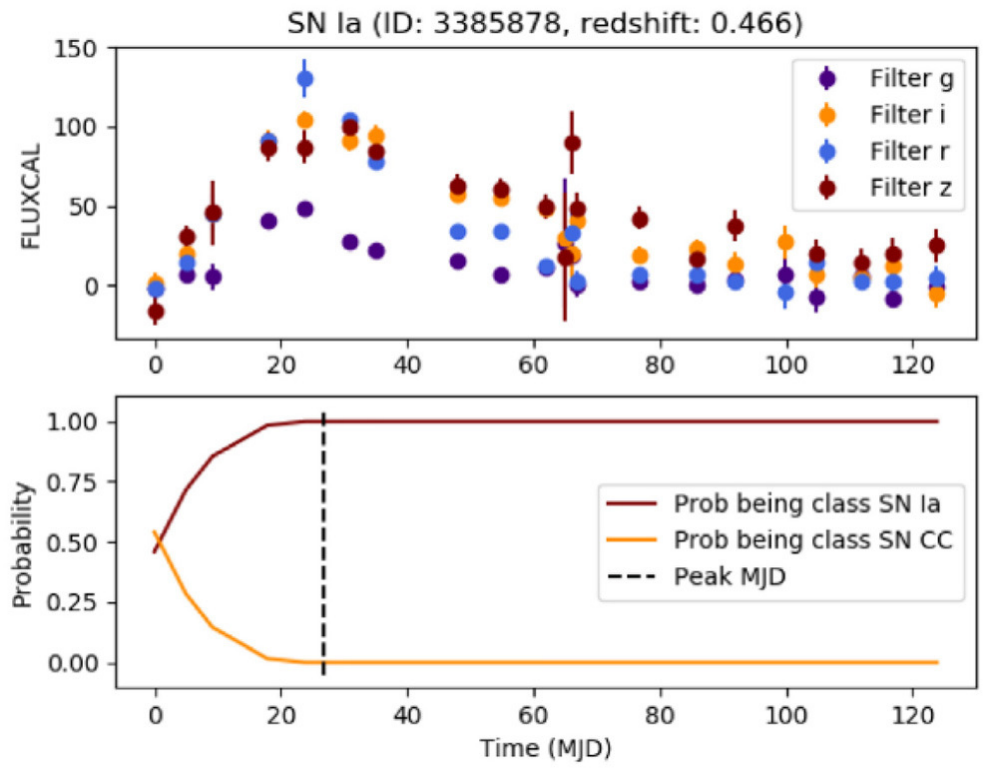
Simulated type Ia supernova light-curve and classification. Top: calibrated flux evolution in different DES band-passes as a function of normalized time (the first photometric measurement is set to time equals zero). Bottom: Baseline RNN classification probability evolution with respect of time, no host-galaxy redshift information was provided. At each photometric measurement, classification probability is obtained. The maximum light of the simulated supernova is shown in a gray dashed line and the simulated redshift of the supernovae is shown on the top *z* = 0.466. We highlight that redshift is not used for this classification but can improve results. Our baseline RNN classifies this light-curve as type Ia SN with great accuracy before maximum light, it only requires a handful of photometric epochs. (Möller and de Boissiére, 2019).

Data representation used in a particular domain influences both the computation system and data storage. One global classification for data representations across domains can be considered as being into raw vs. reconstructed data. The data representation often varies depending on the stage of the reconstruction and the upstream steps in the data processing pipeline. Existing applications include fully connected NNs that often take pre-processed expert feature variables as inputs or CNNs when the data is of image nature. On-going development of domain knowledge-inspired NN algorithms could further take advantage of the expert features in the accuracy and efficiency as detailed below. To fully exploit the power of advanced NNs and bring it closer to data creation for minimum information loss, a more suitable representation of the raw data, e.g., as point clouds, needs to be employed. Prominent representations for raw data from different experimental and measurement systems are:

- **Spatial Data**: Used for describing physical objects in geometric space. There are two main types, called vector and raster data. Vector data, in turn, can be comprised of points, lines, or polygons. Raster data refers to a grid of pixels, such as images, but pixels can also represent other measurements such as intensity, charge, field strength, etc.
- **Point Clouds**: Can be considered a type of spatial data. This data representation is created by collating a set of spatial data, i.e., points in a 3D space, that usually form an object in space collectively.
- **Temporal Data**: Used to represent the state of a system/experiment at a particular time. Data collected across time, in a specific order, is classified in this manner. Time-series data is a subset of this representation, where data is sampled at regular time intervals. An example of time-series data can be seen in Figure 3, for the specific case of supernova classification.
- **Spatio-Temporal Data**: Measurements and observations of a system can be collected across both the space and time dimensions. In that case, the data can be considered spatio-temporal.
- **Multispectral Data**: Used to represent outputs of multiple sensors that capture measurements from multiple bands of the electromagnetic spectrum. Multispectral representation is commonly used in the context of imaging, involving sensors that are sensitive to different wavelengths of light. This usually involves in the order of a few to 10s of spectra.
- **Hyperspectral Data**: Used to represent measurements from a high number of spectra, e.g., in the order of 100s. These images collected from different narrow-band spectra are combined into a so-called hyperspectral cube with three main dimensions. The first two reference the 2D spatial placement (e.g., earth's surface) while the third dimension represents the complete spectrum content at each “pixel” location.

In Table 1, we match these data representations to scientific application domains and give a brief description. We highlight the data representations which are particularly important for a specific domain. We will give more detailed examples below.

**Table 1:** Types of data representations and their relevance for the scientific domains discussed in this paper; ✓✓= Particularly important for domain, ✓= Relevant for domain.

**Domain**	**Spatial**	**Point cloud**	**Temporal**	**Spatio-**	**Multi/Hyper-**	**Examples**
				**Temporal**	**spectral**	
LHC	✓✓	✓✓	✓	✓	–	Detector reconstruction
Belle-II/Mu2e	✓✓	✓✓	–	–	–	Track reconstruction
Material Synthesis	✓	–	✓	✓✓	✓✓	High-speed plasma imaging
Accelerator Controls	✓	–	✓✓	–	–	Beam sensors
Accelerator neutrino	✓✓	✓✓	✓	✓	–	Detector reconstruction
Direct detection DM	✓✓	✓✓	✓	✓	–	Energy signatures
EIC	✓✓	✓✓	✓	✓	–	Detector reconstruction
Gravitational Waves	✓	–	✓✓	–	–	Laser inference patterns
Biomedical engineering	✓✓	–	–	✓✓	–	Cell and tissue images
Health Monitoring	✓	–	✓✓	✓	✓	Physiological sensor data
Cosmology	✓✓	✓✓	✓✓	✓	✓✓	Lensing/radiation maps
Plasma Physics	✓	–	✓✓	✓	–	Detector actuator signals
Wireless networking	–	–	✓✓	–	–	Electromagnetic spectrum

Cost of data communication (in terms of latency) and data storage (in terms of the cost of acquiring and managing the physical storage resources) present important challenges. Particularly, application domains, which require real-time analysis and/or real-time feedback demand highly optimized data analytics solutions. Applications that rely on hyper-spectral data are faced with an ever-increasing rate of data input across the electromagnetic spectrum. High-speed data reduction is required in these domains. Applications that generate large-scale point clouds similarly demand efficient compression on their spatial data. Application domains that handle multi-spectral data with limited spatial resolution require ultra-fast reconstruction in order to enable real-time control feedback. Another challenge is posed by applications that rely on accurate analysis of streaming time-series data, yet they are forced to perform under highly limited storage and communication resources, either due to privacy and security concerns or limitations of the associated edge devices.

Some current efforts in developing ML solutions to data processing front-ends focus on developing autoencoder based compression engines (Herwig et al., 2020; Loncar et al., 2020). ML-based dimensionality reduction for hyper-spectral data is another direction which has drawn attention (Agar et al., 2019). Deep learning-based approaches are investigated for image reconstruction; the field of material sciences being one of the most active fields in that regards (Schmidt et al., 2019).

#### 3.1.1. Expert Feature DNNs

One straightforward approach to building powerful domain-specific ML algorithms is to start with expert domain features and combine them in a neural network or other multivariate analysis technique. This embedded expertise has inherent advantages because the input features are interpretable, and correlations between features can yield insight into a particular task while optimizing performance. Furthermore, depending on the computational complexity of the domain features, the computation efficiency of such a machine learning approach can be greater than the direct use of raw features. However, the downside is that, by using expert features, we rely entirely on the informativeness of such new features.

Therefore, there is a lot of interest in automating the process of building informative new features from raw features. In image classification tasks, for example, a lot of progress has been made in extracting high-level data representations through deep neural networks DNNs (Goodfellow et al., 2016). In DNNs, layers of neurons above the original input signal are built to ensure that each new layer captures a more abstract representation of the data. Each layer constructs new features by forming nonlinear combinations of the features in the layer below. This hierarchical approach to feature construction has been effective in disentangling factors of variation in the data (Hinton and Salakhutdinov, 2006; Bengio et al., 2013; Goodfellow et al., 2016), and has been useful to construct informative and meaningful representations. In astronomical images, for example, a DNN starts with low-level pixel information, gradually capturing at upper layers edges, motifs, and eventually entire objects (e.g., galaxies) to provide a broad view of the Universe (Dominguez Sanchez et al., 2018; Huertas-Company et al., 2018). The same applies to other fields of science. For example, detecting particles in large accelerators requires transforming low-level signals into dynamic patterns that can be ascribed to specific particles (Belayneh et al., 2020). In medical imaging, there is a need to quickly identify abnormal tissue from low-level pixel information by gradually capturing global tissue patterns (Bychkov et al., 2018). The importance of transforming the initial input data into meaningful abstract representations cannot be overstated: it remains one of the most powerful properties of modern neural network architectures.

Several challenges exist in the construction of increasingly abstract representations using DNNs. One challenge is to incorporate domain knowledge (e.g., physical constraints) into the neural network model. This is important to address the need for excessive amounts of data when training a DNN and narrow the gap in representational bias between the model and target concept. Under scarce data but abundant domain expertise, adding domain knowledge can expedite the training process (Xie et al., 2021), as well as improving the model generalization performance. Another challenge is to develop tools for model interpretability by explaining the semantics of the representations embedded at each layer (Chakraborty et al., 2017). This is challenging due to the distributed representation of information in the network architecture.

Despite the lack of a formal mechanism to attain a seamless integration between a statistical model and domain knowledge, current approaches point to interesting directions, e.g., using knowledge to add training data or to change the loss function (Vo et al., 2017). Model interpretability in DNNs has seen an upsurge in research over the past years (Chakraborty et al., 2017). Commonly, studies look at individual units and their activation patterns to elucidate what is learned across layers of neurons.

#### 3.1.2. Frame-Based Images

**Figure 4:**
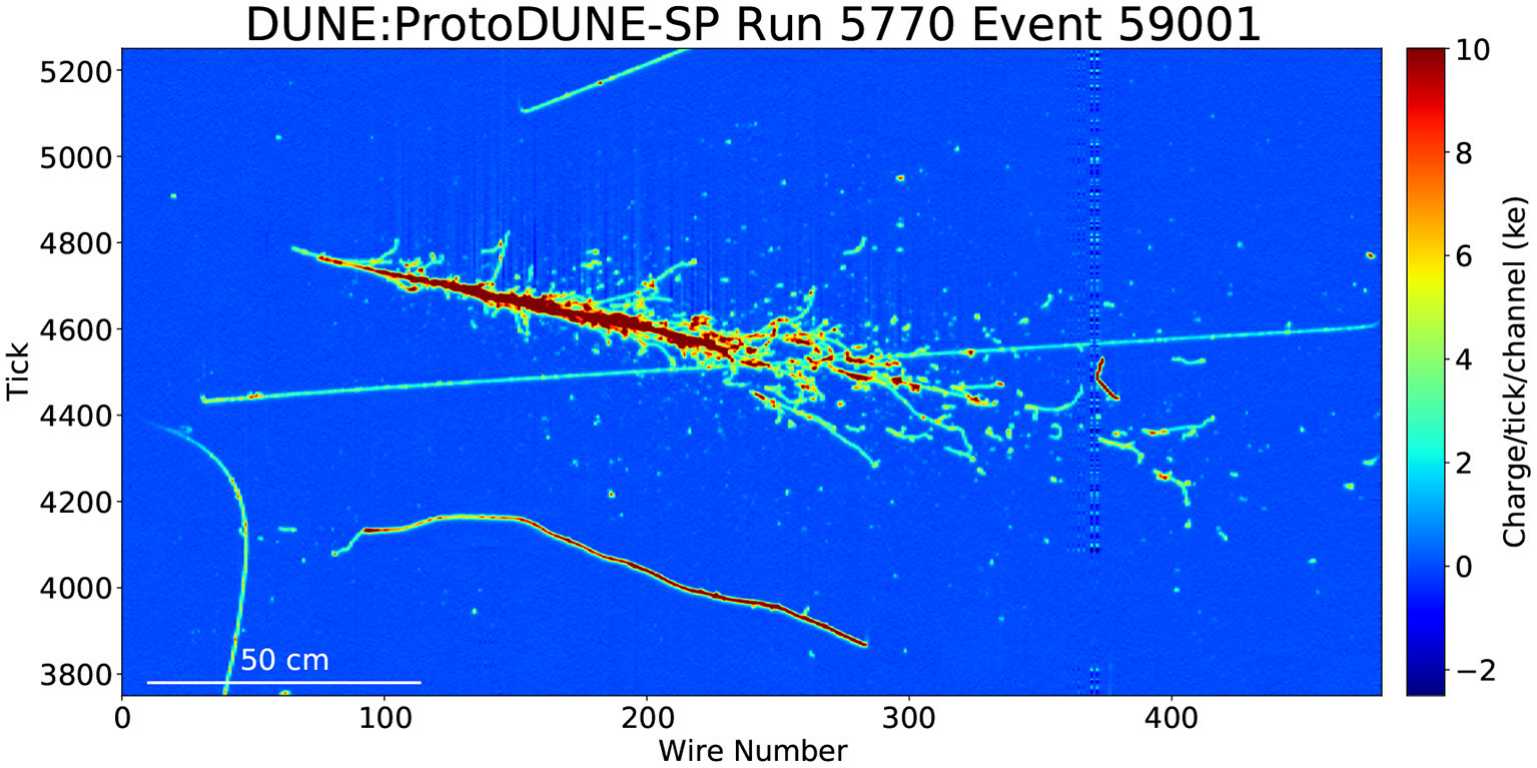
A 6GeV/c electron event in the ProtoDUNE detector. The x-axis shows the wire number. The y-axis shows the time tick in the unit of 0.5μ*s*. The color scale represents the charge deposition.

Frame-based images are a suitable representation of the experimental data in multiple domains such as neutrino detection with time projection chambers in particle physics. An example of this data representation can be seen in Figure 4 for an electron deposition in the ProtoDUNE neutrino detector. A spatial frame is shown by plotting the time coordinate “Tick” and wire position in space. Recent developments in neural network architectures exploit the sparsity of the images to reduce the computation complexity for real-time/accelerated ML applications. Other types of experimental data in HEP and many other domains can also be processed to be represented as frame-based images, although often not without information loss.

#### 3.1.3. Point Clouds

Point cloud data representation is often used in HEP, where multiple frames of event-based measurements collected by a large number of detectors are combined into a data set. Across many HEP applications point clouds commonly help to represent particle jets with data sizes exceeding Pb/s. More broadly, point clouds can be used to capture any 3D space event and interactions of moving parts in space. For CMS, remnants of proton-proton collisions create sensors signals in a customized and optimized detector geometry and points are illustrated in space. Various types of scan-based imaging data can be represented as point clouds. Other domains such as CT and PET scanning in biomedical engineering and virtual reality also utilize this representation for imaging. 3D scanners used for product design, solid object modeling, architecture, and infrastructure design leverage point clouds as well. Many of these imaging tasks generate point clouds of sizes in the order of several GB to TB. Domains sharing point cloud representation (e.g., HEP and biomedical imaging) also commonly involve spatial characteristics.

#### 3.1.4. Multi-/Hyperspectral Data

**Figure 5:**
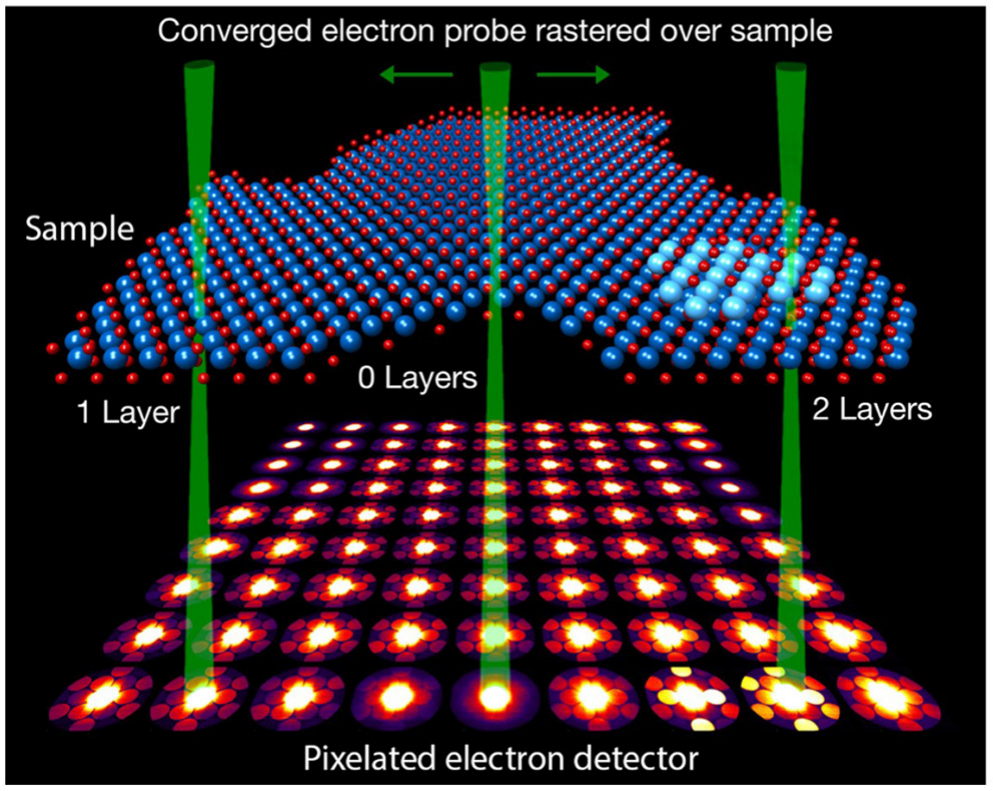
Experimental 4D-STEM measurement of a dichalcogenide 2D material. Atomic map is inferred from the data, each diffraction pattern represents an average of 7 × 7 experimental images, green STEM probes are labeled for regions of the sample with one layer, vacuum, and two layers (Ophus, 2019).

Multispectral data is common between wireless health monitoring and wireless communication systems. A set of physiological sensors, often representing different modalities, are combined into a multispectral data set for health monitoring and intervention systems. For wireless communication, signal interference and network traffic conditions are captured via multispectral data. Both domains capture this data across the time domain, so also exhibit temporal features. Furthermore, in both domains generated data size can be considered relatively smaller (ranging from 100s of Mb/s to 10s of Gb/s), compared to the rest of the domains discussed in this article. Hyperspectral data is used across many astronomy applications, medical imaging, and electron microscopy, which is used to drive many materials science design and discovery applications. An example of hyperspectral data in electron microscopy is shown in Figure 5. An electron probe is rastered over a sample under study and diffraction patterns are captured on a pixelated detector. The pixelated detector captures many images as the electron probe is scanned across the sample. Emerging multimessenger astronomy applications further emphasize the utility of hyperspectral data representations combining observations from a wide array of detectors and telescopes.

#### 3.1.5. Time-Series Data

Time-series data is common in experiments that observe dynamically evolving systems in processes such as synthesis for material discoveries or the temporal evolution of the plasma state in nuclear fusion experiments. It can be a measurement of high-speed temporally resolved imaging in material science or physics features (density, temperature, current, radiation, fluctuations, etc.) or spatial features of evolving plasma state, as a function of time. *In-situ* diagnostics of the time-series data can either provide alerts to terminate an experiment early that indicates undesired outcome in material science without performing the entire experiment and offline analysis that is time-consuming and computationally expensive, thus improves the experiment operation efficiency and accelerates discoveries of material of desired properties. In the accelerator controls at the Fermilab Booster accelerator, for example, magnet voltages that steer proton beams around a synchrotron are recorded at 15Hz time samples. This study builds a digital twin which is used to simulate the Booster data. Furthermore, to reliably predict and avoid large-scale major disruptions in nuclear fusion experiments, real-time analysis of the time-series data is crucial in guiding the action needed in experimental prediction and control.

### 3.2. System Constraints

In this section, we present an overview of desired system properties and constraints that are prevalent across a number of application domains. Unique challenges are arising from each scientific application based on sensing technology, the physical processes, and the timescales and data rates, and bandwidth. These system constraints result in specific choices of data processing platforms, often with multiple compute architectures across the data continuum, such as the choice of FPGA-based systems vs. embedded processors, GPUs, or custom ASICs. Table 2 summarizes several scientific application domains along with their event rates, system latency constraints and performance requirements, and deployment characteristics. We broadly define platforms for integration fast machine learning techniques into “soft,” software programmable coprocessors, and “custom,” custom embedded computing devices. Software-programmable systems are often preferred because they are less complex to implement while custom embedded solutions are required when software programmable systems cannot satisfy experimental throughput, bandwidth, or latency constraints. We will describe in further detail this distinction below. Examples of these system design choices are the trigger systems for HEP include LHC reconstruction of collision events, the Belle-II experiment, the Mu2e experiment which deploy custom embedded systems. Meanwhile, experiments like the Electron-Ion Collider have data rates that may not require custom hardware solutions and could deploy only software programmable solutions for event reconstruction and real-time processing experiments. One final distinction worth discussing concerns the nature of real-time processing and the *in-situ* vs. post-mortem nature of the inference and analysis tasks. Examples that we consider in classifying tasks that have different requirements are: data reduction which primarily focuses on limiting data collection rates of experiments for offline analysis; real-time processing and data analysis which is required to extract real-time domain features of the data for tasks like filtering/triggering; and closed-loop controls where data processing provides direct feedback to the operation and continuous control of an experiment. These distinctions and their consequences on the computing systems is illustrated in Table 3.

**Table 2:** Domains and practical constraints: systems are broadly classified as soft (software-programmable computing devices: CPUs, GPUs, and TPUs) and custom (custom embedded computing devices: FPGAs and ASICs).

**Domain**	**Event rate**	**Latency**	**Systems**	**Energy-constrained**
**Detection and event reconstruction**				**No**
LHC and intensity frontier HEP	10s Mhz	ns-ms	Soft/custom	
Nuclear physics	10s kHz	ms	Soft	
Dark matter and neutrino physics	10s MHz	μs	Soft/custom	
**Image processing**				
Material synthesis	10s kHz	ms	Soft/custom	
Scanning probe microscopy	kHz	ms	Soft/custom	
Electron microscopy	MHz	μs	Soft/custom	
Biomedical engineering	kHz	ms	Soft/custom	Yes (mobile settings)
Cosmology	Hz	s	Soft	
Astrophysics	kHz–MHz	ms-us	Soft	Yes (remote locations)
**Signal processing**				
Gravitational waves	kHz	ms	Soft	
Health monitoring	kHz	ms	Custom	Yes
Communications	kHz	ms	Soft	Yes (mobile settings)
**Control systems**				
Accelerator controls	kHz	ms–μs	Soft/custom	
Plasma physics	kHz	ms	Soft	

**Table 3:** Classification of domains and their system requirements with respect to real-time needs.

**Domain**	**Real-time data reduction**	**Real-time analysis**	**Closed-loop control**
**Detection/Event reconstruction**			
LHC	Yes	Yes	No
Nuclear physics	Yes	No	No
Dark matter-neutrino	Yes	No	No
**Image processing**			
Material synthesis	Yes	Yes	Yes
Scanning probe microscopy	Yes		
Electron microscopy	Yes		
Biomedical engineering	Yes		
Cosmology	Yes	No	No
Astrophysics	Yes	No	No
**Signal processing**			
Gravitational waves	Yes	No	No
Health monitoring	Yes	Yes	Yes
Communications	Yes	Yes	Yes
**Control systems**			
Accelerator controls	Yes	Yes	Yes
Plasma physics	Yes	Yes	Yes

#### 3.2.1. Software Programmable Coprocessors

Historically, the first attempts at addressing the computational needs of the problems reviewed in this article have been through software-programmable systems. CPU-based local clusters or cloud services as well as cloud computing resources utilizing GPU or TPU-based hardware accelerators are utilized in different applications. One particular concept explored by the HEP community is the GPU as a Service (GPUaaS) model (Krupa et al., 2020). This can further be expanded into the Machine Learning as a Service concept, similarly explored within HEP (Kuznetsov et al., 2020). These paradigms involve the implementation of machine learning modules to solve a set of physics problems, which are then transferred to GPU or TPU accelerators and accessed by the local CPU “client” of the native experimental system.

One of the major system constraints is the computational capacity, which can be defined in terms of a number of floating point operations as far as neural network implementations are concerned. Real-time machine learning methods require an ever-increasing rate of computational capacity as it directly impacts the *latency per task*. The *task* could be a trigger for LHC, reconstruction of an event in accelerator experiments or astrophysics, material synthesis, reconstruction of an image captured by an electron microscope, etc. Extreme parallelism would be desired to provide the highest capacity possible to minimize latency and maximize throughput. In a processor-based system, this can be addressed by increasing the size of the compute cluster. Naturally, facility costs impose a limit on the scale of these clusters. Another constraint is the available amount of storage coupled with the cost of data movement across the memory hierarchy. In the majority of the use cases, the latency involved with moving data from the front-end (detectors, microscopes, sensors, etc.) dominates the total latency. One of the prominent performance constraints is related to the utilization and subsequent latency of the network that links the front-end with the back-end. Current limitations on the speed of data movement renders the CPU/GPU cluster-based systems unable to meet the real-time requirements.

#### 3.2.2. Custom Embedded Computing Devices

As the latency and throughput constraints are coupled with challenging practical energy constraints, efforts have been directed toward specialized computing systems to address the hard real-time needs. An increasingly attractive paradigm is to design components that are finely optimized for specific steps in the data capture workflow. These components can be mapped onto FPGA devices or they can be designed and manufactured as an application-specific integrated circuit (ASIC). In the LHC and accelerator domains, there is a rich set of FPGA-based demonstrations of front-end data processing systems, which meet microsecond latencies. These systems are in charge of tasks such as triggering, event reconstruction, and anomaly detection. Direct and naive implementations of neural networks to perform inference for these tasks can fail to meet the latency requirements since they often incur significant resource utilization. The highest achievable FPGA clock frequency and inference latency is correlated with the resource utilization and percentage occupancy of the device. Co-design techniques developed for these applications particularly specialize in extreme quantization and pruning (with an awareness of accuracy) so that resource requirements can be controlled aggressively to ensure inference latency targets. These optimizations push the resource usage envelope as far as down as 10s of percent of the FPGA device in order to meet the system constraints and yet demonstrate implementations with high inference accuracy.

Some other applications (e.g., accelerator controls, biomedical and health applications) impose less stringent latency expectations, in the order of ms, where the urgency for resource minimization is alleviated. Hence, the focus of the system design can shift from extreme resource economy to enhanced sophistication in the algorithms that are being mapped to the device. Inference models can now include deep(er) learning models coupled with advanced video and signal processing engines, as well as local privacy-preserving processing tasks (applicable particularly to mobile health and networking and communication applications).

For mobile and IoT-based deployment of the edge devices, resource efficiency emerges as an important factor as it impacts energy consumption. However, in these applications, energy efficiency can also be achieved by alternative means. One option would be selective powering, i.e., creating a resource-rich full-featured baseline implementation, which still comfortably meets latency constraints if energy was not an issue, and introducing power gating or standby features to modulate energy consumption during periods of low/no activity.

There are system constraints, which point the designers to a custom ASIC solution in addition to or in place of FPGA devices. ASICs can address extreme form factor considerations, integration of computation with sensing (e.g., smart photon detectors) into compact front-end devices, tight integration with other mixed-signal or analog functionalities, radiation hardening requirements, and ultra-low energy budgets.

## 4. Technology State-of-the-Art

In this section, we aim to give an overview of technologies and techniques for building fast ML algorithms. This requires *codesign*: building algorithms with hardware in mind and providing efficient platforms for programming the hardware. Sections 4.1, 4.2 focus on neural network design and training for efficient implementation in hardware. In Sections 4.3, 4.5, we classify our discussion of ML hardware compute platforms into two categories: “Conventional CMOS Hardware” and “Emerging Beyond CMOS Hardware.” The former will address nearer-term hardware solutions, while the latter will focus on the speculative end of the spectrum. Meanwhile, because the area of programming new hardware is rapidly moving, we lay out an example of the options and challenges for one device family: FPGAs. This is presented in Section 4.4, and from the details for FPGAs we hope the reader also gets a sense of the fundamental approaches for designing software for emerging hardware.

### 4.1. Systematic Methods for the Efficient Deployment of ML Models

As discussed in Section 2, many ML problems in science require low latency, often with constrained resources. However, most of the current state-of-the-art NN models have prohibitively high latency with a large memory footprint and energy consumption. For this reason, practitioners have been forced to use sub-optimal models (e.g., shallow NNs) with non-ideal accuracy to avoid this latency problem. There is a large body of literature that has focused on solving this problem by making NN models more efficient (in terms of latency, memory footprint, and energy consumption). These efforts could be broadly categorized as follows: (i) Designing new efficient NN architectures; (ii) NN and hardware co-design; (iii) Quantization (low precision inference); (iv) Pruning and sparse inference; and (v) Knowledge distillation. Here we briefly discuss each of these approaches.

*Designing New Efficient NN Architectures:* One line of research has been focused on finding new NN models that are efficient by design. A notable early work is SqueezeNet (Iandola et al., 2016), a new NN model without any expensive Fully Connected layers, along with a new lightweight *Fire module*, that resulted in a 50 × smaller model as compared to AlexNet, but with the same accuracy. Later on, several new innovations were made in efficient NN architecture design. One focus has been to find efficient layers/operators. Notable works are group convolutions (Ioannou et al., 2017), depthwise convolutions (Howard et al., 2017), spatial separable convolutions (Mamalet and Garcia, 2012), shuffle layers (Ma et al., 2018), and shift convolutions (Wu et al., 2018a), to name a few.

Another focus has been to find similar substitutes to *Fire module* that are more efficient and result in better accuracy/generalization. Notable works include residual networks (He et al., 2016) (originally designed to solve issues with vanishing gradients, but these structures are generally more efficient than non-residual architectures), densely connected networks (Huang et al., 2017), squeeze-and-excite modules (Hu et al., 2018a), and inverted residual blocks (Sandler et al., 2018).

These classical techniques mostly found new architecture modules through a manual design search. This is not scalable, and as such recent approaches have proposed automated methods that use neural architecture search (NAS). NAS methods automatically find the right NN architecture for a given constraint of model size, depth/width, and/or latency. The high-level approach here is to train a probabilistic *SuperNet* that includes all possible combinations of NN architectures within the prescribed constraints, but with learnable probabilities. After this SuperNet is trained, one can sample an architecture from its learned probability distribution. Notable works include RL based methods (Zoph and Le, 2016), efficient NAS (Pham et al., 2018), MNasNet (Tan et al., 2019), DARTS (Liu et al., 2018), and Differentiable NAS (Wu et al., 2019).

*NN and Hardware Co-design:* Another promising line of work has been to tailor the NN architecture for a specific hardware platform, and/or co-design them together. This is quite promising for configurable hardware such as FPGAs. The importance of hardware-aware NN design is that the cost of performing different types of operations varies for different hardware. For example, hardware that has a dedicated cache hierarchy can execute bandwidth bound operations much more efficiently than hardware without a cache hierarchy. Notable works in this area include SqueezeNext (Gholami et al., 2018), where both the NN and the hardware accelerator were co-designed with a manual tuning approach. More recent works have proposed to automate hardware-aware design through NAS. Notable works include ProxylessNAS (Cai et al., 2018), OnceForAll (Cai et al., 2019b), FBNet (Wu et al., 2019), and MobileNetV3 (Howard et al., 2019).

*Quantization (Low Precision Inference):* A common solution is to compress NN models with quantization (Asanovic and Morgan, 1991; Hubara et al., 2016; Rastegari et al., 2016; Zhou et al., 2016, 2017; Cai et al., 2017, 2020b; Choi et al., 2018; Jacob et al., 2018; Zhang et al., 2018a; Dong et al., 2019; Wang et al., 2019c; Chin et al., 2020; Gholami et al., 2021), where low bit-precision is used for weights/activations. A notable work here is Deep Compression (Han et al., 2016), which used quantization to compress the model footprint of the SqueezeNet model discussed above, bringing its size to 500x smaller than AlexNet. In quantization, the model size is reduced without changing the original network architecture, and it could potentially permit the use of low-precision matrix multiplication or convolution. Therefore, both the memory footprint and the latency could be improved.

The quantization methods can be broadly classified into two categories of *Post-Training Quantization* (PTQ), and *Quantization-Aware Training* (QAT). In PTQ, a pre-trained model in single precision is quantized to low precision without any fine-tuning or re-training (Banner et al., 2018; Lee et al., 2018a; Choukroun et al., 2019; Meller et al., 2019; Nagel et al., 2019; Zhao et al., 2019c; Cai et al., 2020b; Fang et al., 2020a,b; Hawks et al., 2021). As such, these quantization methods are typically very fast, and, in some cases, do not even require any training data (Nagel et al., 2019; Cai et al., 2020b; Haroush et al., 2020). However, PTQ often leads to high accuracy degradation, especially for low precision quantization. To address this, some quantization methods adopt QAT to re-train the model after the quantization, so that the parameters can get adjusted. This approach often results in higher accuracy, but at the cost of longer time associated with re-training the model (Courbariaux et al., 2015; Lin et al., 2015; Hou et al., 2016; Hubara et al., 2016; Rastegari et al., 2016; Zhou et al., 2016, 2018a; Zhu et al., 2016; Cai et al., 2017; Gysel et al., 2018; Huang et al., 2021).

Another differentiator is the use of *simulated quantization* (aka fake quantization), vs. *integer-only* quantization (Lin et al., 2016; Jacob et al., 2018; Yao et al., 2020b; Kim et al., 2021). In the former, the weights/activations are stored in low precision, but they are cast to higher precision during inference. In the latter, there is no casting involved, and the multiplication and accumulation also happen in low precision. Using integer-only quantization has the advantage that one can speed up inference by using low-precision logic for multiplication and addition, besides reducing the memory footprint of the model.

**Figure 6:**
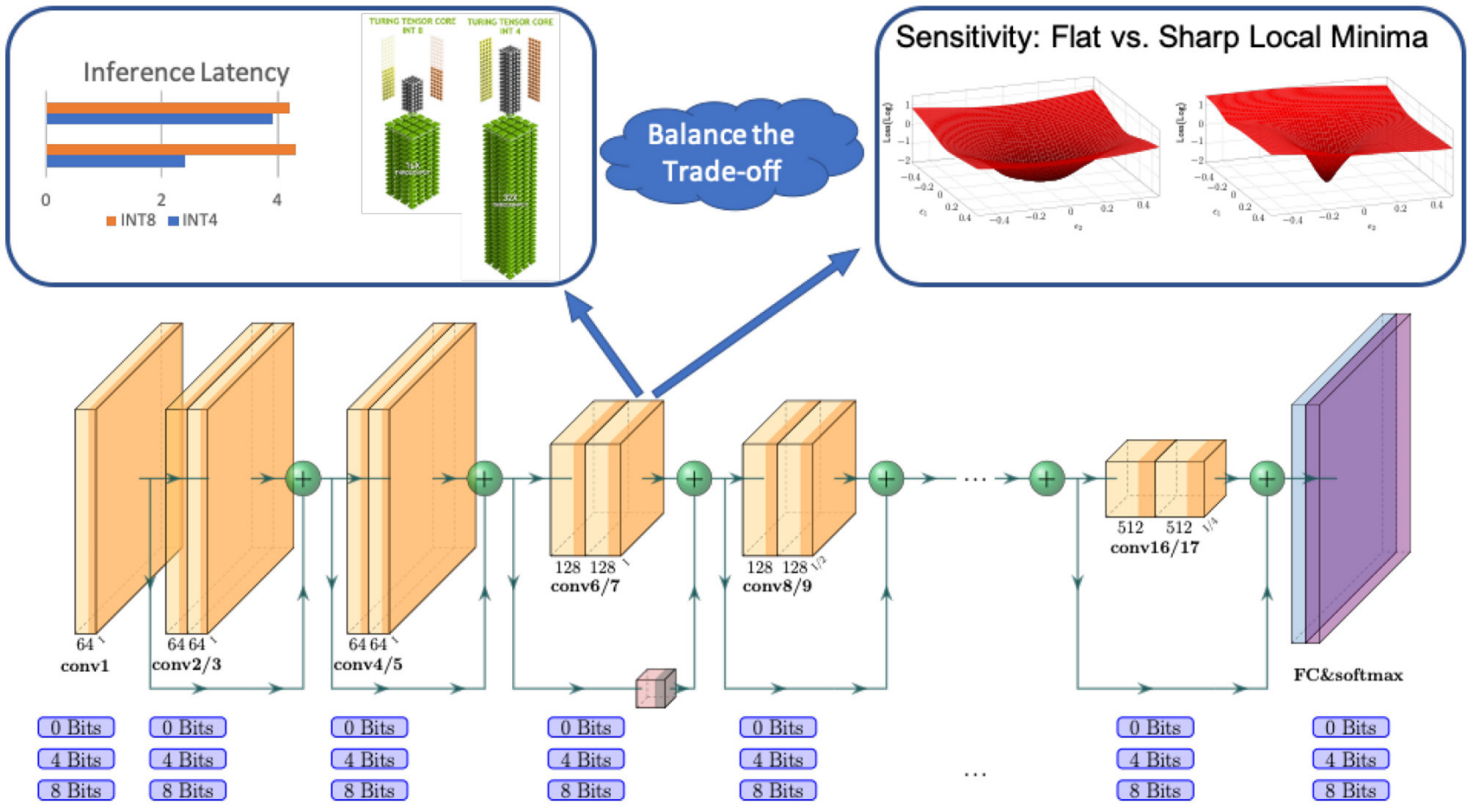
The illustration of hardware-aware quantization and pruning. A given NN model can be compressed by using low precision quantization instead of single precision. The extreme case is to use 0-bit quantization which is equivalent to removing/pruning the corresponding neurons. The goal of compression is to find the best bit-precision setting for quantization/pruning to reduce model footprint/latency on a target hardware with minimal generalization loss.

Another distinction is *hardware-aware quantization*. Similar to NN architecture design, quantization can also be tailored for specific hardware platforms. This becomes important for mixed-precision quantization (Wu et al., 2018b; Zhou et al., 2018b; Dong et al., 2019, 2020, 2021; Wang et al., 2019b; Shen et al., 2020; Yao et al., 2020b). The reason is that certain operations in the NN model may benefit more from low precision quantization than others, based on whether they are bandwidth bound or compute-bound. As such, as schematically illustrated in Figure 6, one must determine the best precision setting based on the tradeoff between the potential footprint/latency gain and the sensitivity to accuracy degradation.

*Pruning and Sparse Inference:* Another approach reducing the memory footprint and computational cost of NNs is to apply pruning, which could be thought of as quantization to 0-bits. In pruning, neurons with small *saliency* (sensitivity) are removed, which results in a sparse computational graph (LeCun et al., 1990). Here, neurons with small saliency are those whose removal should minimally affect the model output/loss function. Pruning methods can be broadly categorized into unstructured pruning (LeCun et al., 1990; Hassibi and Stork, 1993; Dong et al., 2017; Lee et al., 2018b; Xiao et al., 2019; Park et al., 2020), and structured pruning (Luo et al., 2017; He et al., 2018; Huang and Wang, 2018; Lin et al., 2018; Yu et al., 2018; Zhao et al., 2019a). Unstructured pruning removes neurons without any structure. With this approach, one can remove most of the NN parameters with little impact on the generalization performance of the model. However, this approach leads to sparse matrix operations which are hard to accelerate and are typically memory-bounded (Buluc and Gilbert, 2008; Gale et al., 2019; Blalock et al., 2020; Hoefler et al., 2021). This can be addressed with structured pruning, where a group of parameters (e.g., an output channel) is removed. However, the challenge here is that high degrees of structured pruning often lead to significant accuracy degradation.

In both approaches, the key question is to find which parameters to prune. A simple and popular approach is magnitude-based pruning (Hanson and Pratt, 1988; Mozer and Smolensky, 1988; Chauvin, 1989; Li et al., 2016b; He et al., 2017, 2019; Liu et al., 2017; Lin et al., 2020a). In this approach, the magnitude of parameters is used as the pruning metric. The assumption here is that small parameters are not important and can be removed.

An important problem with magnitude-based pruning methods is that parameters with small magnitudes can actually be quite sensitive. It is easy to see this through a second-order Taylor series expansion, where the perturbation is dependent on not just the weight magnitude but also the Hessian (LeCun et al., 1990). As such there are several works that use second-order based pruning (LeCun et al., 1990; Hassibi and Stork, 1993; Hassibi et al., 1993; Wang et al., 2019a; Yu et al., 2021).

Finally, we should mention that it is possible to combine pruning and quantization together to compress the NN model. In fact, pruning could be viewed as quantization to 0-bits. The recent work of Hawks et al. (2021) proposes a quantization-aware pruning method and applies to high energy physics problems; It reports better results than pruning or quantization alone.

*Knowledge Distillation:* Model distillation (Romero et al., 2014; Hinton et al., 2015; Li et al., 2017; Mishra and Marr, 2017; Yim et al., 2017; Polino et al., 2018; Ahn et al., 2019; Yin et al., 2020) trains a large model and then uses it as a teacher to train a compact model. Instead of using class labels during the training of the student model, the key idea of model distillation is to leverage the soft probabilities produced by the teacher, which can guide/help the student training.

Previous methods of knowledge distillation focus on exploring different knowledge sources. Hinton et al. (2015), Li et al. (2017), and Park et al. (2019) use logits (the soft probabilities) as the source of knowledge, while Romero et al. (2014), Yim et al. (2017), and Ahn et al. (2019) try to leverage the knowledge from intermediate layers. The choices of teacher models are also well studied, where You et al. (2017) and Tarvainen and Valpola (2017) use multiple teacher models to jointly supervise the student model, while Crowley et al. (2018) and Zhang et al. (2019b) apply self-distillation without an extra teacher model. Other previous efforts apply knowledge distillation with different settings on different applications. Lopes et al. (2017), Nayak et al. (2019), and Yin et al. (2020) study data-free knowledge distillation, and Wang et al. (2018a) and Wang et al. (2020e) combine knowledge distillation with GANs.

A major challenge of knowledge distillation methods is to achieve a high compression ratio. Compared to quantization and pruning which can usually maintain accuracy at 4 × compression, knowledge distillation methods tend to have non-negligible accuracy degradation at those compression levels. But these two approaches are orthogonal, and recent works have shown that their combination can result in high accuracy/compression (Mishra and Marr, 2017; Polino et al., 2018; Mao et al., 2020; Yao et al., 2020b). It should be mentioned that current distillation methods are mostly applied to classical ML problems, and few works have looked into their application in Science AI problems.

### 4.2. Systematic Neural Network Design and Training

There is currently no analytical approach to find the right NN architecture for a given task and training dataset. Originally, designing the NN architecture was mostly a manual task with intuitions that were often *ad-hoc*. However, in recent years there has been a lot of innovations in automating the NN architecture design process, which is referred to as Neural Architecture Search (Zoph and Le, 2016; Cai et al., 2018, 2019b; Liu et al., 2018; Pham et al., 2018; Tan et al., 2019; Wu et al., 2019).

NAS could be viewed as a hyperparameter tuning problem, where the hyperparameters are the design choices for a NN architecture. This could include width, depth, types of operations, etc. The main challenge is that the search space for the operation types scales exponentially with the number of layers. As such, one has to still include some high-level intuition about the NN architecture to limit the search space.

After limiting the search space, the general NAS process is as follows: A candidate architecture is sampled from the set of all possible architectures and is then trained for a number of epochs on the training dataset. The accuracy is then used as the metric to evaluate how good that candidate architecture is. Then based on this reward, the probability distribution of sampling architectures is updated. This process needs to be repeated for many different candidate architectures (sometimes exceeding hundreds of thousands). Inherently, this leads to another problem related to tuning the optimization hyper-parameters for each candidate architecture. For example, if a good architecture is sampled from the NAS but is trained with sub-optimal hyperparamters, then the error will be high and the NAS algorithm will reduce the likelihood of sampling that architecture which is not the desired property.

As a result, *scalability* has become an integral concern for any procedure in the presence of “big data.” One main class of procedures for which scalability has become indispensable is in numerical optimization algorithms, which are the core of training methods. There is a large body of literature on designing efficient numerical optimization/training methods (Gupta et al., 2018; Reddi et al., 2018; Shazeer and Stern, 2018; Zhang et al., 2019c; Ginsburg et al., 2020; Liu et al., 2020a; Ma, 2020; Park et al., 2020; Yao et al., 2020c; Zhuang et al., 2020) as well as efficient NAS algorithms to search for the right NN architecture (Zoph and Le, 2016; Liu et al., 2018; Pham et al., 2018; Tan et al., 2019; Wu et al., 2019).

For the optimization, the goal is to design new methods that require fewer iterations to converge and are more robust to hyper-parameter tuning. One notable advancement here is the ability to apply second-order methods without the need for forming the second-order operator (Gupta et al., 2018; Reddi et al., 2018; Yao et al., 2019, 2020c). It has been shown that the performance and robustness of these methods are higher than first-order optimization methods on classical ML problems (e.g., in computer vision or natural language processing). Interestingly, some recent results for Physics Informed Neural Networks (PINN) (Raissi et al., 2019) have found that first-order methods work significantly sub-par to (quasi) second-order methods. This could potentially provide opportunities to adapt or redesign some of the second-order algorithms for Science problems.

For the NAS algorithms, the goal is similar, which is to find methods that require evaluating fewer candidate architectures, with less manual restriction or tuning of the search space. Another goal is to design transferable NAS algorithms that can be trained on a small problem and then transferred to larger problems that are more expensive (Cai et al., 2018, 2019b).

In summary, the core of designing NN architecture is to have a fast method of sampling architectures (through NAS), and the fast training of the sampled architectures (through fast and robust optimization algorithms).

### 4.3. Hardware Architectures: Conventional CMOS

As the prevalence and demands for machine learning rapidly continue to grow, it is increasingly important that we design machine learning algorithms efficiently and simultaneously deploy them on complementary and powerful hardware platforms. The compute and memory demands of NN deployments are huge and growing beyond the limits to where standard silicon-based semiconductors can scale. The reasons behind the scalability challenges in the semiconductor industry are as follows: Firstly, as we approach the End of Moore's Law, transistor cost has been exponentially rising due to rising chip design costs with shrinking technology nodes (as published by Xilinx and Gartner in 2011 already Trimberger, 2018). Furthermore, with the end of Dennard scaling, we've encountered considerable thermal challenges as power density no longer remains constant between node generations. To mitigate the challenges of increasing thermal density, chips are now designed to conditionally deliver power to groups of transistors, effectively throttling or "turning off" parts of a chip. This technique has come to be known as creating dark silicon (Esmaeilzadeh et al., 2011).

To overcome these challenges and provide sufficient compute capabilities, many disruptive approaches have been proposed. For example, Cerebras Systems (Cerebras, 2019) has brought to market the first computer system which employs **wafer scale integration**. where chips are built from complete wafers rather than individual dies. Such a technique brought with it substantial engineering challenges in regards to power delivery, packaging, and cooling. Exploring the other dimension, foundries are investigating true **3D chip stacking** as was presented at HotChips'2019 by TSMC (Hotchips, 2019). Even analog computing, quantum computing, and in-memory computing are investigated as well. See a more detailed discussion on beyond CMOS neuromorphic computing in Section 4.5 below.

Less risky approaches focus on moving away from traditional von Neumann architectures, using specialization of compute architectures to provide the necessary performance scaling and energy efficiency. Due to the specialization, the devices become increasingly heterogeneous. A huge range of devices has emerged that all try to address this problem in different ways, whereby the key challenge is: How do we loop transform and unfold the algorithms best to maximize data reuse and compute efficiency, minimize memory bottlenecks, and limit power consumption while meeting real-time requirements?

The choice of hardware type and quantity often boils down to a set of constraints imposed by compute environment (datacenter, cloud, on-premise, edge, mobile), workload type (inference, training), data type (Language, Time Series, Vision, Graph, etc.), ML model, usage model (online inference, batch jobs), and user-centric Service-Level Agreements (encryption level, request latency, etc). For large datacenter deployments handling various types of workloads, it is often the case that several platforms must be combined to reduce Total Cost of Ownership (ToC) across all their hardware platforms. It has therefore become increasingly necessary for owners of heterogeneous platforms to think of their systems as large-scale multi-processor computers, a trend sometimes termed Warehouse Scale Computing (Luiz André Barroso, 2009). For Deep Learning hardware accelerators, these new computers generally take the form of CPU co-processors: a host CPU communicates with other entities in the datacenter, interfaces with disk memory, and formats input data which is then offloaded to the accelerator responsible for executing a user-defined compute graph, or Neural Network.

We begin with a taxonomy of these hardware architectures and discuss their relevant characteristics when it comes to the acceleration of machine learning workloads. This is essential to understand how they will differ in their execution behavior, what it takes to leverage their unique features and how they can potentially benefit from previously introduced optimization techniques.

*Taxonomy of Compute Architectures for Deep Learning:* A broad range of hardware architectures to deploy machine learning algorithms exists today. We can broadly classify them by the following criteria:

1. Basic type of compute operation
2. Inherent support for specific numerical representations
3. External memory capacity (which is mostly relevant for training workloads)^3^
4. External memory access bandwidth
5. Power consumption in the form of thermal design power (TDP)
6. Level of parallelism in the architecture and the degree of specialization

**Figure 7:**
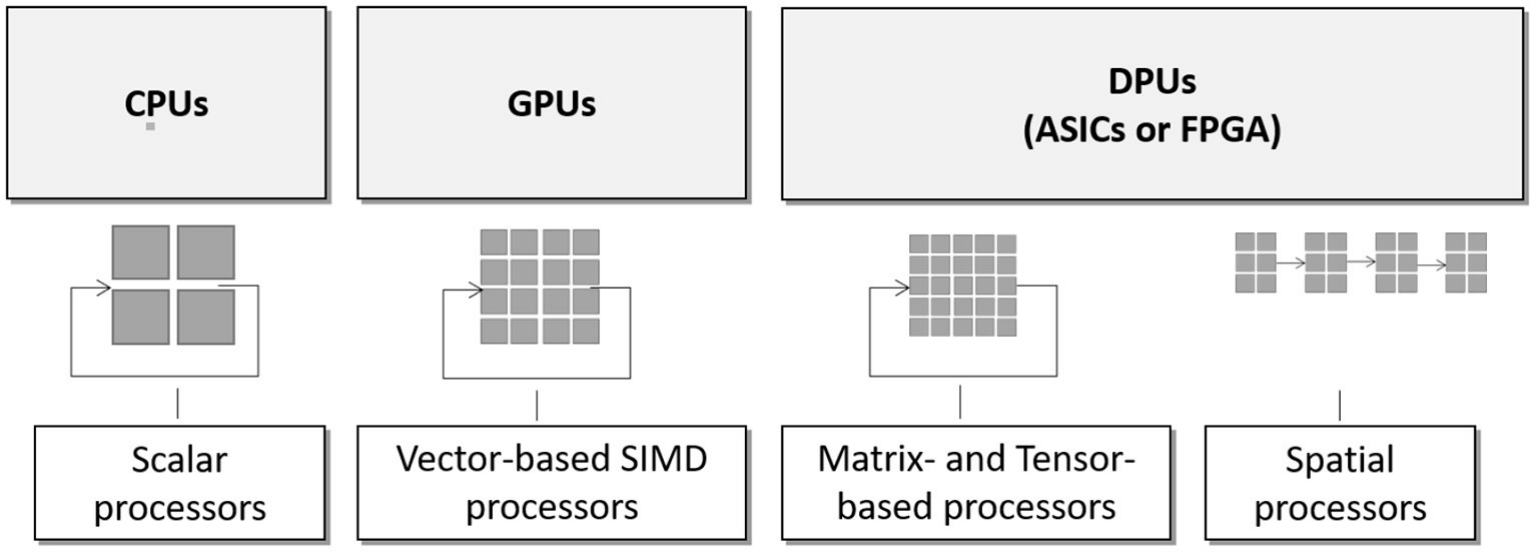
Taxonomy of compute architectures, differentiating CPUs, GPUs and DPUs.

As is shown in Figure 7, we classify the compute architectures into scalar processors (**CPUs**), vector-based processors (**GPUs**), and so-called deep learning processing units (**DPUs**), although realistically these categories blend to some degree. DPUs are specialized for this application domain whereby we distinguish the more generic matrix- or tensor-based processor and a spatial processing approach. DPUs can be implemented with either ASICs or FPGAs. All of these architectures will be discussed individually below.

*CPUs:* CPUs are widely used for ML applications and are viewed as largely serial or scalar compute engines (even though high-end variants for cloud deployment may have up to 10s of cores). They are optimized for single-thread performance, with implicitly managed memory hierarchies (with multiple levels of caches), and support floating point operations (FP64 and FP32) as well as 8bit and 16bit integer formats with dedicated vector units in most recent variants. Theoretical peak performance tops at 6.8TOPs for FP64 assuming boost clock speed (Cascade lake, 56 cores, 3.8GHz). External memory is currently primarily leveraging DDR4 memory banks with large capacities: Intel's Cascade Lake offers up to 4.5 TebiByte (2^40^ Bytes) which is beyond what any of the other device categories can offer. Access is at maximum speed through high-end hardened memory controllers, offering 282 Gbps bandwidth (for example Cascade Lake with 12 DDR4 channels). Compared to GPUs and other HBM-enabled devices, the memory bandwidth of CPUs is lower. However, for many use cases, this can be compensated through their sophisticated cache hierarchies, combined with mature compiler tools. Regarding power consumption, CPUs are at the upper end of the spectrum with high-end devices range up to 400 W (Cascadelake, 2019). In the embedded space, ARM processors provide generally popular solutions, in particular when performance requirements are very low and when functionality is required that is not supported by the specialized device variants. In particular, the Ethos (Skillman and Edso, 2020) family of processing cores is specialized for CNN workloads and as such is considered under the DPU category below. The advantages of CPUs are the generality of the hardware, as well as the ease of programming where design environments have matured over decades. As expected this comes at the cost of lower peak performance and less efficiency compared to the more specialized device families. In regards to quantization, CPUs can only leverage this optimization technique for INT8 and INT16 if supported.

*GPUs:* GPUs are SIMD-based (Single Instruction, Multiple Data) vector processors that support smaller floating point formats (FP16) natively, as well as fixed point 8-bit and 4-bit integer formats more recently, and have a mix of implicitly and explicitly managed memory. NVIDIA GPUs are some of the most popular hardware targets for machine learning, and newer families of chips have been introduced to specifically accelerate this workload, with AMD not far behind. The latest devices in NVIDIA's Volta and Turing architecture families, introduced in 2018 and 2019, respectively, offer up 130TOPs in FP16, which is beyond the capabilities of the latest CPU generations. As such they are amongst the highest performant devices in the market for the acceleration of DNNs as they can exploit the high degree of parallelism inherent in this application via increasingly specialized architectural features. For example, NVIDIA's Volta is the first generation to incorporate tensor cores as a new feature, as well as improved FP32 and FP64 support for training in a data center setting (Durant et al., 2017), and also introduced a deep learning accelerator (DLA) in their embedded devices to further reduce power consumption. This specialization brings additional challenges for their usage; there are up to 3 distinct execution units now, namely CUDA cores, tensor cores, and the DLA, which don't operate on the workload simultaneously (at least not easily or by default). We, therefore, don't sum up the peak performance of different execution units, but use only the maximum. AMD announced the Vega GPU (Exxactcorp, 2017) with new deep learning instruction set operations, with the goal of obtaining parity with NVIDIA's high-end Tesla V100 datacenter GPUs. Also, AMD's most recent EPYC family supports customized instructions for deep learning (Epyc, 2019). Both companies offer also low power GPUs for the embedded space, namely the AMD Vega mobile GPU (Hardawar, 2018) and NVIDIA Jetson TX2 (Franklin, 2017) and AGX family (AGX, 2019).

In regards to memory, GPUs leverage specialized and highly pipelined GDDR memory, which reduces capacity, but offers much higher bandwidth (up to 732GBps). With NVIDIA's Turing family the latest devices include HBM2 DDR memory stacks (Turing, 2019), which scales the memory access bandwidth to 1TBps and beyond. Again this is particularly important to address the needs of training workloads. For the same reason, some of the DPUs introduce HBM2 as well, as discussed below. In regards to power consumption, GPUs are high, up to 345 W.

One general challenge for GPUs is that they need to leverage input parallelism to achieve high utilization of their large compute arrays. Therefore, before execution inputs need to be grouped into batches, which has adverse effects on end latency. Further, GPUs are relatively high in power consumption. Regarding quantization, support is limited to the inherent datatypes, which are INT4 at smallest in the context of NVIDIA's Turing family, and INT8 for many of the others. Finally, the corresponding software environments for GPUs, while not on the same level as CPUs, have matured significantly and provide increased ease of use.

*FPGAs and ASICs:* FPGA and ASIC customize hardware architectures to the specifics of a given application. They can be adapted in all aspects to suit a use case's specific requirements. This includes their IO capability, their functionality, or even to suit specific performance or efficiency targets. FPGAs can be reprogrammed whereas ASICs are fully hardened. This flexibility allows for amortizing the design costs of the circuit across many applications but comes at the expense of hardware resource cost and performance.

FPGAs are a popular choice for the acceleration of CNNs. Traditionally, an FPGA compute fabric consist of a sea of lookup tables (LUTs) which are interconnected through a programmable interconnect. The latest generations host millions of LUTs. Furthermore, the fabric is interspersed with specialized hardened compute blocks (DSPs) which accelerate n-bit multiply accumulate operations (MACs), as well as SRAM blocks. The latter are referred to as block RAMs (BRAMs), which hold 36 kbits, and Ultra RAMs (URAMs) which store 288 kbits. More recent FPGA generations combine multiple FPGA dies, referred to as super logic regions (SLRs), and leverage a silicon interposer to provide connectivity between SLRs. This technology is referred to as stacked silicon interconnect (SSIT) and helps scale device capacity.

*DPUs:* As mentioned at the beginning, the term DPU (short for deep learning processing unit) refers to a new type of compute architecture, specialized for the acceleration of CNNs. DPUs are customized for these types of applications in a number of ways: types of operations supported, direct support of tensors or matrices, inherent data types and supported numerical representations, macro-architecture, explicitly managed and specialized memory hierarchies, and which levels of parallelism they exploit (input, output pixel, IFM, OFM, bit, and layer and branch parallelism) as was introduced in the first part of this chapter. We differentiate two types of DPUs, which can be implemented with both ASIC technology and FPGAs.

**Figure 8:**
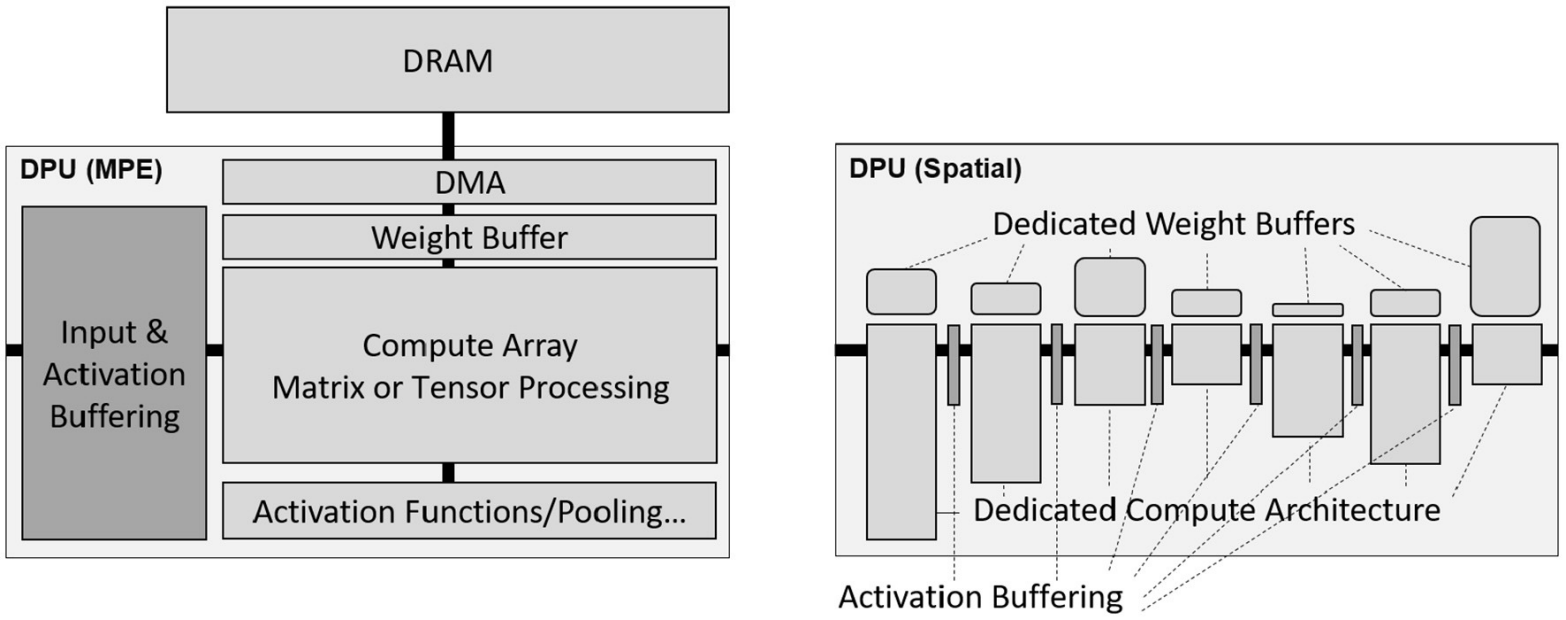
DPU architectures: Matrix of Processing Engines (MPE) on the left, and spatial architecture on the right.

*Matrix of Processing Elements (MPE):* The first type, as shown on the left side of Figure 8, consists of an MPE that operates on matrices or higher dimensional tensors. The processing engines can be simple MACs, vector processors, or more complex VLIW (Very Long Instruction Word) cores that can support concurrent execution of different instructions. A popular example in this category is Google's Tensor Processing Unit (TPU). Introduced in 2016 (Sato et al., 2017), it was originally designed to accelerate Google's TensorFlow framework. The first generation supported integer arithmetic with a massively parallel INT8 matrix-multiply engine. The second generation TPU was announced in May 2017 (Jouppi et al., 2017), and the third generation in May 2018 (Teich, 2018). These newer chips boast improved memory performance as well as support for floating point specifically aimed at training. There are a number of startups introducing custom hardware that fall into this category. Within the cloud, there are Graphcore, Groq, and Wave Computing. Within the embedded space, where the design constraints are even more stringent, we find even more solutions. Most are secretive about the details of their designs. Intel is investigating several custom accelerators and has for that purpose acquired a number of startups, namely Nervana, Habana, and Movidius. Fathom (Armasu, 2016) is Movidius' ultra low power Neural Compute Stick (NCS) which operates at about 1 W. Also, ARM offers specialized CNN processors in the form of their Ethos family, boosting performance up to 4TOPs with support for INT8 and INT16 datatypes.

As mentioned above, DPUs provide specialized datatypes to execute heavily quantized, reduced precision CNN implementations. At the extreme, binarized neural networks (which are very high throughput at extremely low power) are exploited in the following ASICs: BinarEye (Moons et al., 2018), BNN Custom Fabric (Ando et al., 2017), and IBM AI Accelerator (IBM, 2018). Also, Lattice has announced binarized neural network libraries targeting low power FPGA and achieving 1 TOPs/W (Lattice, 2018). Custom floating point representations are also considered. For example, Microsoft's Brainwave project (Chung et al., 2018) uses this approach with the aim of applying FPGAs to CNNs at datacenter scale. However, typically the hardened versions in ASICs only support INT8, as lower precisions could potentially limit their application scope. FPGA-based MPE implementations such as Xilinx's xDNN are less constrained and in principle can be customized as needed.

Similar to the GPU, but perhaps to a lesser degree, DPUs leverage input, IFM (input feature map) and OFM (output feature map) parallelism, which requires buffering of inputs and may have adverse effects on latency as well. A particular challenge arises in the context of software environments, which differ for all vendors and are less mature than what we have observed for CPUs and GPUs. Typically, they are limited to support execution of very specific layer types (sometimes even restricted in regards to parameter ranges) and neural networks, whereby the range of layer types and neural network models is continuously expanding.

In summary, through their specialization, these implementations minimize hardware cost, maximize performance and optimize efficiency by exploiting specific precision arithmetic with a specialized instruction set and customized memory system. However, in order to gain a performance advantage, the algorithms need to be adapted to leverage these features.

*Spatial DPUs:* The second type of DPU leverages spatial acceleration and exploits layer and branch parallelism. Popular examples are hls4ml and FINN (Umuroglu et al., 2017; Blott et al., 2018). To that extent, the hardware architecture is even further specialized to the specifics of a given deep learning topology. This is visualized on the right side of Figure 8. The hardware architecture actually mimics the given deep learning topology and the inputs are streamed through the architecture. Every layer is instantiated with a dedicated compute datapath. Each layer has a dedicated weight buffer, and activation buffers in-between layers are FIFOs of minimal size. They buffer just enough data to feed the next set of convolutions in the next layer. This is substantially more efficient compared to the first type of DPUs or GPUs and yields reduced latency.

DPUs and GPUs generally perform a layer-by-layer compute, where a sequence of images has to be buffered in order to extract maximum compute out of the platform (input, IFM and OFM parallelism). For this, the device buffers a batch of images before computing the first layer of all images. Then all intermediate results are buffered, and then the next layer is computed, and so on. Hence the latency is heavily dependent on the size of the input batch.

As a result, spatial DPUs have an advantage in regard to latency. This level of customization is only possible with programmable hardware architectures such as FPGAs, as they can adapt the hardware architecture for different use cases. This generally wouldn't make sense in the context of an ASIC accelerator, as that would yield an ASIC only capable of accelerating one specific topology, which would be far too restrictive in scope. The limitation in spatial architectures is the scalability in the numbers of layers. Each layer comes at a resource cost overhead and there is a maximum number of layers that can be created within a single device. As a result, some extremely deep CNNs might not be able to fit into a single device. Microsoft's Brainwave project leverages spatial computing and overcomes this limitation with a distributed approach (Chung et al., 2018).

Once a spatial DPU has been leveraged and the architecture is specialized for a very specific CNN, the architecture can be further customized in regards to minimum precision. By supporting only the bits as needed per layer of the CNN they can achieve even higher performance and efficiency, while in an MPE, the hardware will support the maximum precision that is required over the whole network. In regards to customized precisions and spatial architectures, FINN has pioneered the first binarized neural network accelerators (Fraser et al., 2017; Umuroglu et al., 2017) and provided many proof points for customized reduced precision implementations (Blott et al., 2018). This flexibility comes at a cost, in the form of programming complexity, and they are extremely difficult to characterize in general, as the performance characteristics depend on the specifics of the hardware architecture that has been implemented.

*Further Variants of DPUs:* Beyond the previously discussed spatial DPUs and MPEs, there are many more variants. Some exploit sparse computing engines for example, such as EIE and its successor ESE (Han et al., 2017), SCNN (Parashar and Rhu, 2017), Cnvlutin (Albericio et al., 2016), Cambricon-S and Cambricon-X (Zhang et al., 2016). These are the only architectures that can benefit from irregular sparsity. Finally, another dimension for customization of precision is to optimize over the execution- or run-time of a CNN. In other words, beyond using statically fixed reduced precision, where the hardware operates with a fixed precision for all variables, some approaches explore run-time configurable bit precision which allows for the exploitation of bit-parallelism in the arithmetic. On the hardware implementation side, this can be exploited with run-time programmable precision and is effective with **bit-serial** implementations. For example Umuroglu et al. (2018) demonstrate with BISMO that bit-serial can provide highly attractive performance with minimal overhead on FPGAs, while Judd et al. Judd et al. (2016) show the same is true for ASICs with their prototype ASIC called Stripes. While this concept can be applied to both MPE and spatial architectures, it makes the most sense for MPEs.

*Summary of Conventional CMOS Hardware Architectures:* We analyzed three categories of hardware architectures that are leveraged for CNN inference, namely common CPUs, SIMD-based vector processors such as GPUs, and DPUs which are specialized architectures for the acceleration of deep learning workloads. An overview of the architectures is visualized in Table 4. Please note, "Ease of Use" includes compute kernel programmability as well as general ease of use. The degree of specialization includes operators, precision support, and customization toward topologies. In summary, for DPUs, we distinguish between tensor processors which leverage a matrix of processing engines and spatial architectures which can be further specialized for specific topologies using FPGAs. CPUs are the most general solution but high in power. GPUs and DPUs offer the highest performance, though GPU are more expensive in energy cost. Spatial DPU architectures excel at low latency and provide the highest compute efficiency through maximized customization. CPUs, GPUs, and DPUs (MPE) use a sequential layer-by-layer compute model whereas spatial DPUs execute all layers of the network concurrently. Hardened topologies in form of ASICs, CPU and GPU offer a fixed set of native dataypes, whereas FPGAs can adopt any precision and numerical representation, which provides the utmost flexibility and leverages optimization with quantization to the maximum, whereas hardened approaches need to default to the next higher supported precision into which the reduced precision variable can be embedded. However, the programmability in the FPGA fabric also comes at a speed and energy cost. All architectures can benefit from coarse-grained pruning optimization techniques. Only sparse execution engines can benefit from irregular pruning, such as synaptic pruning. We also discussed the various deployment options. Many devices offer different power and operating modes as different compromises between throughput and power consumption to adapt to the potentially very different optimization targets of different application settings. Similarly, batch sizes, thread counts and stream sizes offer another compromise in regards to throughput vs. latency. Again this is to facilitate a spectrum of different use cases. Finally, the table shows that speculative approaches such as Cerebras can bring fundamental performance scalability. Overall, each approach comes with its own advantages and disadvantages and the best solution greatly depends on the specifics of a given use case.

**Table 4:** Characterization of types of hardware based on important metrics.

**Server-class**	**Throughput**	**Latency**	**Power**	**Ext. Mem. Bandwidth**	**HW specialization**	**Ease of Use**	**Training/Inference**
**Conventional**							
CPU	Medium	High	High	Medium	Low	High	Both
DPU-MPE	High	Medium-high	Medium	High	Medium	Low-medium	Inference
DPU-Spatial	High	Low	Medium	High	High	Low	Inference
GPU (NVIDIA A100)	High	High	High	High	Medium	High	Both
**Speculative**							
Cerebras CS-1	Very high	Medium	High	Very High	Medium	Medium	Both

### 4.4. Hardware/Software Codesign Example: FPGA-Based Systems

In the last decade, we have observed the rise of two significant paradigms that have come to scientific applications: heterogeneous-computing systems and machine learning. Heterogeneous computing can overcome the decline of Moore's Law and Dennard Scaling and achieve the desired computational cost and performance by executing portions of the applications on the best-matched hardware, e.g., CPU, GPU, ASIC, and FPGA. On the other hand, machine learning is an automatic process that creates programs that can solve classes of problems. As with traditional programming, machine learning can significantly benefit from heterogeneous computing; in addition, designers can tailor specialized but reprogrammable hardware to fit ever-changing machine learning requirements. This section examines tools and methodologies that can automatically deploy and orchestrate machine learning on FPGA systems in larger scientific applications. FPGAs are a particularly compelling example to explore because the efficiency of the hardware coupled with their programmability makes for an interesting case study in hardware/software codesign.

Traditional software programming is complicated, and parallel high-performance programming is even more challenging. Programming heterogeneous systems that integrate FPGAs bring the challenge to the next level: the programmer must deal with a multi-objective optimization problem that involves performance and costs, i.e., hardware resources. For machine learning applications, a common practice is to profile the application on CPU (or GPU) to identify the bottlenecks to be offloaded onto the reprogrammable logic to improve latency, throughput, or energy efficiency of the application as a whole. Then, part of the application can remain on the CPUs to control the execution and interact with the rest of the scientific setup.

*FPGA Programming:* FPGA are configurable integrated circuits that provide a good trade-off in terms of performance, power consumption, and flexibility with respect to other hardware paradigms. However, it is a challenging and lengthy task to program FPGAs. FPGA programming has traditionally been a job for hardware designers familiar with digital design and computer architecture. These requirements lead to a steep learning curve for software developers and other domain experts. In order to lower the entry barrier, there has been a growing focus on designing FPGA hardware at a higher level of abstraction. As a result, various approaches have brought FPGA development into the mainstream by allowing developers to design for FPGAs at a higher level using familiar languages such as C, C++, OpenCL, and in some cases, even C# (Singh and Greaves, 2008). Here an important question arises: what are the additional advantages of designing the hardware at a higher level of abstraction? High-level languages (HLLs) include various constructs and design patterns that are more functionally expressive. Furthermore, the amount of time spent in the verification of the design is also a crucial factor. Hardware-description languages such as Verilog or VHDL focus on the final implementation details and, because of that, are more verbose. Bigger code repositories are not easy to verify for functional correctness. On the other hand, HLLs are more compact and simulate faster. Thus, a designer can do more verification in the same span of time. Despite these advances, FPGA programming remains complex. This has compelled academia and industry to develop new compilers, frameworks, and libraries to facilitate hardware design.

*High-Level Synthesis and Languages:* High-level synthesis (HLS), also known as behavioral or algorithmic synthesis, is an automated design process that takes as input a functional description of a design and outputs an RTL implementation. It transforms an untimed (or partially timed) high-level specification into a fully timed implementation. The process of HLS starts by analyzing the data dependencies between the various operations in the functional description. This analysis leads to a Data Flow Graph (DFG) representation. After the DFG generation, during the allocation phase, HLS maps each operation onto a hardware resource with latency and area characteristics. Then, HLS adds the notion of time to the design during the scheduling phase. Scheduling takes the operations and resources of the DFG and decides in which clock cycle to execute them, given their latency information. This step infers sequential logic by adding registers between operations and creating finite state machines (Fingeroff, 2010).

Over the past three decades, many HLS tools have been proposed. The work in Nane et al. (2016) presents an evaluation of different academic and commercial HLS tools tested on the same set of benchmarks. These tools have different input languages, perform different internal optimizations, and produce different quality results, even for the same input languages. The results show that each HLS tool can significantly improve performance once the designer has mastered benchmark-specific optimizations and constraints. However, academic HLS tools have a higher learning curve because of a minor focus on usability. Commercial HLS tools have an advantage because of their better documentation, robustness, and design verification integration.

In terms of input languages for HLS, most of the HLLs are variants of the C language. However, there are a few limitations to generate hardware from a pure C specification. First, C lacks the notion of timing and concurrency. The designer must rely on the HLS tool to create clock-based timing. Similarly, the designer must specify the concurrency model or rely on HLS to extract the parallelism among operations or processes. Second, C lacks bit-accurate data types. It only provides “native” data types such as char, int, and long, whose size is a multiple of a byte. Third, it lacks the concepts of hardware interfaces and communication channels. SystemC was adopted as HLS language to address all of these limitations (Ren, 2014). However, SystemC still has not entirely made inroads in the FPGA community. Another common problem with all C-based languages, including SystemC, is memory access and modeling. These languages have a flat memory model, and memory access is done through pointers. Either HLS has to decide how to implement the memories in hardware, or the designer must leverage additional HLS directives or libraries to model the memory sub-system properly. Finally, in the family of the C-based specification languages for HLS, the SYCL language is emerging. SYCL (pronounced sickle) is an industry-driven standard that adds parallelism to C++ to design heterogeneous systems. SYCL programs perform best when paired with SYCL-aware C++ compilers such as the open-source data-parallel C++ (DPC++) compiler (Reinders et al., 2020).

Apart from the variations of C, Bluespec is an open-source language for the description and synthesis of hardware based on SystemVerilog. It provides levels of abstraction with a clean semantic that highlights aspects of the architecture. It can be considered a high-level functional HDL, where modules are implemented as rules using SystemVerilog syntax. Those rules are called guarded atomic actions and express behaviors as concurrently cooperating finite state machines (FSMs). Another recent language among FPGA designers is Chisel. It is based on Scala and supports hardware definition using highly parameterized generators, object-oriented and functional programming. Similar to an HLS flow, it compiles into an RTL Verilog implementation.

Although all these languages have helped create efficient hardware and significantly shorten the development time, specific coding techniques are still necessary. Also, the growth and diversification of the application domains have shown the limitations of these programming languages. This has further pushed the level of abstraction to domain-specific languages (DSLs). In recent years, we are observing the growth of a considerable corpus of DSLs and frameworks for FPGA designs (Papadimitrioua et al., 2012; Kapre and Bayliss, 2016). In a DSL-based approach, the users and the tools can use domain knowledge to apply static and dynamic optimizations. However, a domain-specific HLS tool requires an appropriate compiler and a development environment that caters to the target domain. Table 5 shows some of the DSLs and frameworks developed over the years for FPGA computing organized by domains of application. Although all the approaches in the table are diverse in terms of applications, the interesting question is, what are the common denominators? To the best of our knowledge, most of the approaches are broadly based on two approaches: either the DSL specification gets directly compiled into the RTL implementation, or the approach leverages source-to-source compilers. In the latter case, the DSL compiler produces an equivalent source code in a different programming language, for example, C++, for a more standard HLS flow. As a final concluding remark for this paragraph, the efforts for designing better HLS compilers and languages are a significant part of present FPGA research. Furthermore, the work in Table 5 by no means is an exhaustive list. The area of DSLs for FPGA easily outnumbers the work presented in the table.

**Table 5:** A brief taxonomy of domain-specific languages and frameworks for FPGA applications.

**Domain and interfaces**	**DSLs and frameworks**
Signal-processing	HDLCoder (Kapre and Bayliss, 2016), LabView (Kapre and Bayliss, 2016), Spiral (Nordin et al., 2005), VSIPL (Janka et al., 2001)
Networking	SNORT (Mitra et al., 2007), Click (Kohler et al., 2000), P4 (Bosshart et al., 2014), Floem (Phothilimthana et al., 2018)
Databases	Glacier (Mueller et al., 2010)
Machine learning	OptiML (Sujeeth et al., 2011)
Numerics	Verilog AMS (Kapre and DeHon, 2009)
Streaming	Maxeler (Pell and Mencer, 2011), SCORE (Kapre and DeHon, 2011), Lime (Bacon et al., 2013), Aetherling (Durst et al., 2020)
Dataflow	OpenDF (Bhattacharyya et al., 2009), OpenSpatial (Kapre and Bayliss, 2016)
Graphs	GraphStep (Delorimier et al., 2011), GraphGen (Nurvitadhi et al., 2014)
Data parallel	MapReduce (Kapre and Bayliss, 2016), Accelerator (Bond et al., 2010), FCUDA (Papakonstantinou et al., 2009), SuSy (Lai et al., 2020a)
Circuit generators	Flopoco (de Dinechin et al., 2009), JHDL (Bellows and Hutchings, 1998), PAMDC (Bertin and Touati, 1994)
Image processing	HIPACC (Reiche et al., 2017), FROST (Del Sozzo et al., 2017), Darkroom (Hegarty et al., 2014), RIPL Stewart et al. (2018), PolyMage (Chugh et al., 2016)
Static	JBits (Guccione et al., 2000), TVM (Moreau et al., 2018)
Task based	TAPAS (Chi et al., 2021)
Dynamic	PyRTL (Clow et al., 2017), APARAPI (Segal et al., 2014), TornadoVM (Fumero et al., 2019), (Caldeira et al., 2018),
	LINQits (Chung et al., 2013), DHDL (Koeplinger et al., 2016), Spatial (Koeplinger et al., 2018)
Type Systems	DAHLIA (Nigam et al., 2021)
Verification	Kami (Choi et al., 2017b)
Virtualization	Cascade (Schkufza et al., 2019)

*Software and Hardware Integration:* Running an application as software on a microprocessor is more accessible than designing and running specialized hardware, but it may result in poor performance and higher power costs. On the other hand, partitioning an application into software and hardware components is challenging. This process, also known as hardware/software codesign, divides an application between software running on the microprocessor and one or more custom hardware or co-processors components to achieve desired performance goals. Understandably there exists a plethora of research work in this area. The authors in Todman et al. (2005) have provided background information on notable aspects of older FPGA technologies and simultaneously explained the fundamental architectures and design methods for codesign. Furthermore, the work in Choi et al. (2019) is another comprehensive study that aims to evaluate and analyze the microarchitectural characteristics of state-of-the-art CPU-FPGA platforms in depth. That paper covers most of the shared-memory platforms with detailed benchmarks.

The two leading FPGA vendors, Xilinx and Intel, have their own solutions. The Xilinx Runtime Library (XRT) (Xilinx, 2021) is implemented as a combination of userspace and kernel driver components. It supports both PCIe-based boards and MPSoC based embedded platforms. Similarly, Xilinx SDSoc (Xilinx, 2020b) and SDAccel (Xilinx, 2020a) became publicly available later in late 2015; the former works only on select boards of the Zynq family of FPGAs, the latter only on selected PCIe-based boards for OpenCL computing. Since 2020 Xilinx has introduced Vitis (Xilinx, 2020c) as a unified platform. Vitis Unified Software Platform is a comprehensive development environment to build and seamlessly deploy accelerated applications on Xilinx platforms, including on-premises Alveo cards, FPGA-instances in the cloud, and embedded platforms. In addition, the recent efforts of Xilinx under the flagship Versal (Xilinx, 2020d) is also a step toward codesign applications. Intel has the Open Programmable Acceleration Engine (OPAE) (Enno et al., 2020) which is the API library for programmers writing host applications that will leverage the FPGA acceleration. Likewise, Intel oneAPI (Intel, 2020) is an open, unified programming model built on standards to simplify the development and deployment of data-centric workloads across CPUs, GPUs, FPGAs, and other accelerators.

Apart from vendor solutions, academia and the open-source community have also attempted to simplify the integration of applications, operating systems, and hardware acceleration. For a comprehensive analysis, the reader is referred to the works in Eckert et al. (2016) and King et al. (2015), which give a historical review and summary on ideas and key concepts to include reconfigurable computing aspects in operating systems. They also present an overview of published and available operating systems of the last 30 years targeting reconfigurable computing. Similarly, the design exploration and engineering of FPGA drivers that are portable across multiple physical interfaces (PCIe, Ethernet, optical links) have remained a significant part of HW/SW codesign research. The challenges come from the variety of FPGA boards, the plethora of interfaces, and the diverse user requirements. Fundamentally, the FPGA drivers should allow the designer to load or reconfigure an application bitstream and support data transfers between the FPGA and host.

A significant engineering challenge is to consider how to partition driver functionality between the hardware and software components. One growing research focus is to exploit the spatial parallelism of FPGA technology through implementing multiple queues on FPGA drivers. A thorough analysis of system-level drivers for FPGA is out of the scope of our white paper. Readers interested in FPGA system-level drivers are referred to the work in Vipin et al. (2013) and Jacobsen et al. (2015). The authors of those papers have provided benchmarks of various mainstream academic and vendor solutions regarding system-level drivers in the FPGA domain.

Despite various existing OS and driver solutions, an open problem that remains is standardization. An industry-wide standardization would allow for faster development and better portability, and (re)usability of FPGA applications. There is already ongoing work in this area. Standards like the CCIX consortium (CCIX, 2020) and the Heterogeneous System Architecture (HSA) foundation (HSA, 2020) have already made good progress.

*The Case for ML Frameworks for FPGA Design:* Machine learning is one of the fastest growing application domains and over the years there has been an increasing demand for FPGA-based implementations, as the FPGA can achieve latency and throughput and efficiency requirements through extreme customization of the hardware design leveraging reduced precision arithmetic, streaming dataflow implementations (as were introduced as spatial architectures), and fine-granular sparsity. In order to enable a broad spectrum of users with these customizations and to reduce the significant engineering effort, compilers and tools are needed that cater to the needs of ML researchers and domain experts working with FPGAs. Two main ML frameworks are making the effort to fill this vacuum: hls4ml and FINN. Considering the aforementioned tools, compilers, programming languages, and codesign solutions, both hls4ml and FINN have the potential to reach a broader scientific community. To get a better understanding of how such a tool flow works, we consider the FINN compiler in more detail in the following paragraphs.

**Figure 9:**
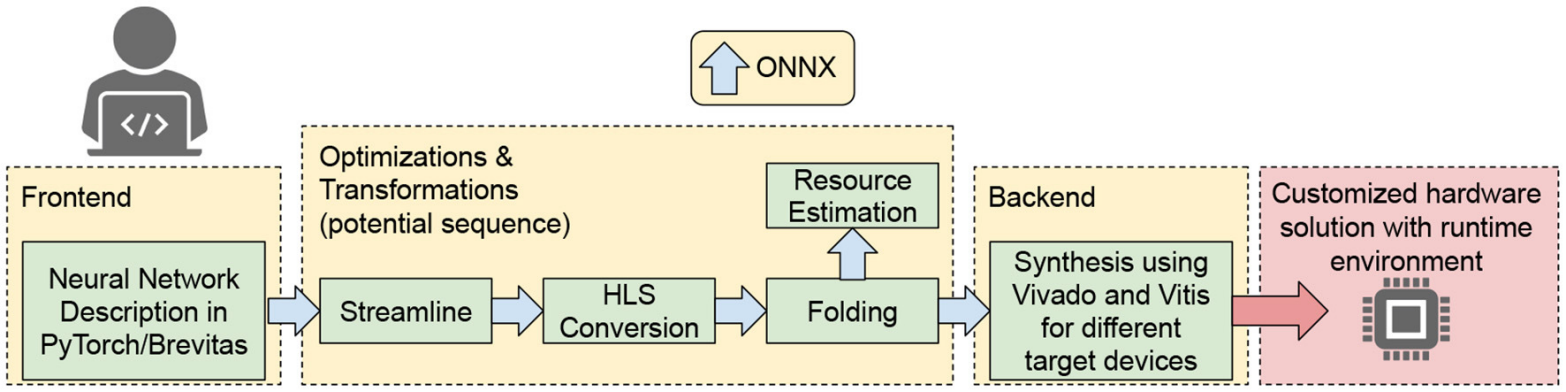
FINN compiler flow.

The **FINN compiler** (Umuroglu et al., 2017) is an open-source framework to generate spatial DPU or streaming dataflow accelerators on FPGAs. The FINN compiler has a highly modular structure as shown in Figure 9, which allows the user to interactively generate a specialized architecture for a specific DNN. The framework provides a frontend, transformation and analysis passes, and multiple backends to explore the design space in terms of resource and throughput constraints. Brevitas (Alessandro et al., 2020), a PyTorch library for quantization-aware training, is the **frontend** used in this work. It enables training DNNs with weights and activations quantized down to a few bits, then exports the trained network into the intermediate representation (IR) used by the FINN compiler. The **transformation and analysis passes** help to generate an efficient representation of the DNN. Finally, the **backend** contains a code generator that creates synthesizable accelerator descriptions, which can be implemented as either a standalone Vivado IPI component or integrated into various shells, including Xilinx Alveo boards and PYNQ embedded platforms.

For further processing, the DNN model must be converted into the IR of the FINN compiler first. The frontend stage takes care of this by converting the PyTorch description into the IR, called FINN-ONNX. This IR is based on ONNX (Bai et al., 2019), an open-source interchange format that uses a protobuf description to represent DNNs. It comes with several standard operators and allows the user to easily create their own operators to customize the model. The nodes represent layers and edges carry outputs from one layer to become inputs to another. The feature to customize the ONNX representation is used in the framework to add application-specific nodes and attributes. Each node is tagged with the quantization of its inputs, parameters (weights and activations), and outputs to enable quantization-aware optimizations and the mapping to backend primitives optimized for quantized computation. During the compiler flow the nodes will be transformed into a backend-specific variants via a series of transformation passes.

The main principle of the **FINN compiler** is **graph transformation** and **analysis passes**, which change or analyze the IR of the model. A pass is a function that takes the IR graph as input and either (a) transforms the DNN by looking for a certain pattern, changing the graph in a specific manner and outputs the modified graph, or (b) **analyzes** the DNN to produce metadata about its properties. To bring the model into a representation from which code can be produced and finally the hardware accelerator can be generated, various transformations must be applied. The main transformations involved are summarized below.

Although the PyTorch description of the network is mostly quantized, it may still contain some floating-point operations from e.g., preprocessing, channelwise scaling or batchnorm layers. In order to generate a hardware accelerator from the model, these floating-point operations must be absorbed into multi-level thresholds, so that a functionally identical network of integer operations is created. The transformation to achieve this is called **streamlining**, as described by Umuroglu and Jahre (Umuroglu and Jahre, 2017). During streamlining, floating-point operations are moved next to each other, collapsed into a single operation, and absorbed into succeeding multi-thresholding nodes.

Next, high-level operations in the graph are **lowered** to simpler implementations that exist in the FINN HLS-based hardware library. For instance, convolutions will be lowered to a sliding window node followed by a matrix-vector node, while pooling operations will be implemented by a sliding window followed by an aggregation operator. The resulting graph now consists of layers that can be converted to hardware building block equivalents. Each node corresponds to a Vivado HLS C++ function call, from which an IP block per layer can be generated using Vivado. The resources utilized by each hardware building block can be controlled through specific attributes passed from FINN to Vivado. For example, multiplications can be performed with LUTs or DSP blocks, and parameters can be stored in distributed, Block, or Ultra RAM.

Finally, the **folding** process assigns compute resources to each layer to obtain the desired throughput with a balanced pipeline by fine-tuning their degree of parallelism. To enable per-layer specialization without reconfiguration and minimize latency, FINN creates dedicated per-layer hardware interconnected with FIFO channels, thus the outermost loop across *L* layers is always fully pipelined. Once the folding is specified, resource estimates can be produced for each node. There are several ways to estimate the resources. Even before IP blocks are generated from the HLS layers, an estimate of the resources per layer can be made by using analytical models based on the concepts from the FINN-R paper (Blott et al., 2018). Estimations can also be extracted from Vivado HLS after IP generation, though these results are still estimations that may differ from the resource usage of the final implementation due to synthesis optimizations.

The **Backend** is responsible for consuming the IR graph and backend-specific information to create a deployment package, also implemented using the transformation concept. To get the inference accelerator, between the layers FIFOs are inserted, which can be sized automatically by the FINN compiler. Afterwards, the single IP blocks are stitched together and synthesized. The stitched IP can be manually integrated into a system, or inserted into an appropriate shell for the target platform. If the target platform is an Alveo card, the design is exported as a Vivado Design Checkpoint (DCP), followed by generation of Xilinx Vitis (Kathail, 2020) object files and linking.

*Summary of Hardware/Software Codesign and FPGA-Based Systems:* In summary, CPUs are the most general solution for CNN inference but high in power. GPUs and DPUs offer highest performance, whereby GPU are more expensive in regards to energy cost. FPGAs offer several tradeoffs that may well fit rapidly moving application domains. FPGAs can adopt any precision and numerical representation, which provides utmost flexibility and leverages optimization with quantization to the maximum, whereas hardened approaches need to default to the next higher supported precision where the reduced precision variable can be embedded. Furthermore, through the spatial dataflow approach, much lower latency can be achieved. However, the complexity of programming FPGAs limits their deployment. Tools such as hls4ml and FINN are frameworks specifically created for the ML domain where they automate the process of hardware generation for the end-user thus hiding the associated design complexity of FPGAs and enabling them for the previously discussed end applications.

### 4.5. Beyond-CMOS Neuromorphic Hardware

With rapidly growing machine learning applications comes the acute need for their efficient hardware implementations. Most of the efforts are focused on digital CMOS technology, such as implementations based on general-purpose TPUs/GPUs, FPGAs, and more specialized ML hardware accelerators. The steady improvements in such hardware platforms' performance and energy efficiency over the past decade are attributed to the use of very advanced, sub-10-nm CMOS processes and holistic optimization of circuits, architectures, and algorithms. It includes, for example, taking advantage of aggressive voltage supply scaling (Moons et al., 2017), very deep pipelines and extensive data reuse in architectures (Chen et al., 2017), and lowering the precision of weights and activations of the algorithms (Simons and Lee, 2019). As a result, very compact state-of-the-art neural networks, such as MobileNet based on 3.4M parameters and 300M multiply-and-add operations per inference (Sandler et al., 2018), can now be fitted entirely on a single chip. However, on all these fronts, advances are saturating and cannot rely on the faltering Moore's law.

On the other hand, further progress would be essential because ML algorithms are getting increasingly more complex. For example, transformer networks (Vaswani et al., 2017), the state-of-the-art approach for many ML tasks today (Vaswani et al., 2017; Vinyals et al., 2019; Dosovitskiy et al., 2020), could have hundreds of billions of parameters and perform hundreds of trillions of operations per inference. Moreover, the transformer's functional performance typically improves with the model size (Brown et al., 2020; Rajbhandari et al., 2020). Training such models requires enormous, data-center-scale (e.g., kiloTPU-year) resources while performing inference on resource-constrained edge devices would be extremely challenging.

The opportunities for building more efficient hardware may come from biological neural networks. Indeed, it is believed that the human brain, with its >1000 × more synapses than the weights in the largest transformer network, is extremely energy efficient (Hasler and Marr, 2013), which serves as a general motivation for developing neuromorphic hardware (Mead, 1990). There is a long history of CMOS neuromorphic circuits (Indiveri et al., 2011). However, unleashing the full potential of neuromorphic computing might require novel, beyond-CMOS device and circuit technologies (Berggren et al., 2020) that allow for more efficient implementations of various functionalities of biological neural systems.

In this section, the most prominent emerging technology proposals, including those based on emerging dense analog memory device circuits, are grouped according to the targeted low-level neuromorphic functionality - see, e.g., reviews in Burr et al. (2017), Bavandpour et al. (2018), Yang et al. (2013), and Yu (2018) and original work utilizing volatile (Ohno et al., 2011; Pickett et al., 2013; Chu et al., 2014; Sheridan et al., 2017; Wang et al., 2017, 2018c; Adda et al., 2018; Lashkare et al., 2018; Zhang et al., 2018b; Cai et al., 2019a; Yeon et al., 2020) and nonvolatile (Mahmoodi et al., 2009, 2019; Alibart et al., 2012; Govoreanu et al., 2013; Prezioso et al., 2015, 2016, 2018; Li et al., 2016a; Adam et al., 2017; Pedretti et al., 2017; Bayat et al., 2018; Hu et al., 2018b; Wang et al., 2018c; Kim et al., 2019; Cai et al., 2020a; Lin et al., 2020b; Liu et al., 2020b; Yao et al., 2020a) memristors, phase change memories (PCM) (Kuzum et al., 2011; Burr et al., 2015; Tuma et al., 2016; Ambrogio et al., 2018; Ríos et al., 2019; Joshi et al., 2020; Karunaratne et al., 2020), and nonvolatile NOR (Bayat et al., 2015; Guo et al., 2017a,b; Mahmoodi et al., 2019), and NAND (Bavandpour et al., 2019, 2020; Lee et al., 2019), and organic volatile (Fuller et al., 2019) floating gate memories, as well as multiferroic and spintronic (Sengupta et al., 2016; Ni et al., 2018; Ostwal et al., 2018; Romera et al., 2018; Grollier et al., 2020), photonic (Bruiner et al., 2013; Vandoorne et al., 2014; Tait et al., 2016; Buckley et al., 2017; Shen et al., 2017; Feldmann et al., 2019; Hamerly et al., 2019; Hamley et al., 2019; Lin et al., 2019; Ríos et al., 2019; Goi et al., 2020; Shasti et al., 2021), and superconductor (Buckley et al., 2017; Segall et al., 2017; Rowlands et al., 2021) circuits. More discussion is devoted to analog vector-by-matrix multiplication circuits in the following subsection because of their immediate value for today's state-of-the-art algorithms. More biologically-realistic proposals described in the subsequent sections are less emphasized because they target algorithms with inferior performance. The least mature though very intriguing quantum neuromorphic computing (Markovich and Grolier, 2020; Yamamoto et al., 2020) is not discussed in this brief review.

**Figure 10:**
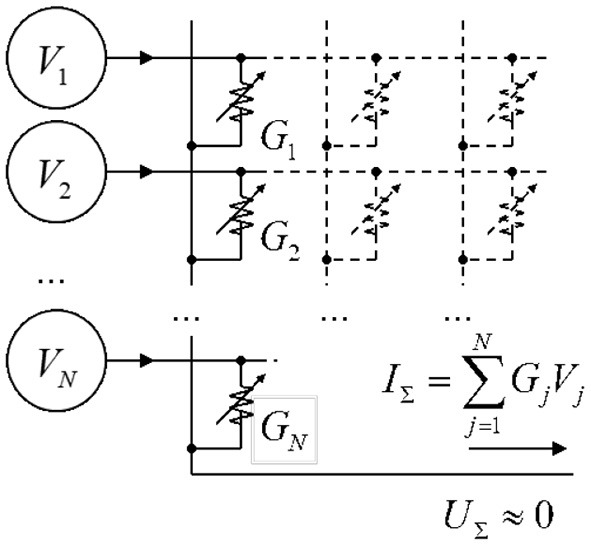
Analog vector-by-matrix multiplication (VMM) in a crossbar circuit with adjustable crosspoint devices. For clarity, the output signal is shown for just one column of the array, while sense amplifier circuitry is not shown. Note that other VMM designs, e.g., utilizing duration of applied voltage pulses, rather than their amplitudes, for encoding inputs/outputs, are now being actively explored see, e.g., their brief review in Bavandpour et al. (2018).

*Analog Vector-By-Matrix Multiplication:* The emergence of dense analog-grade nonvolatile memories in the past two decades renewed interest in analog-circuit implementations of vector-by-matrix multiplication (VMMs) (Widrow and Angel, 1962; Mead, 1990; Holmes et al., 1993; Chawla et al., 2004; Alibart et al., 2012; Bayat et al., 2015; Guo et al., 2017b), which is the most common and frequently performed operation of any neural network in training or inference (Hertz et al., 1991; Gerstner and Kistler, 2002). In the simplest case, such a circuit is comprised of a matrix of memory cells that serve as configurable resistors for encoding the matrix (synaptic) weights and peripheral sense amplifiers playing the role of neurons (Figure 10). The input vector is encoded as voltages applied to rows of the memory matrix so that the currents flowing into virtually grounded columns correspond to VMM results. Because addition and multiplication are performed on the physical level, via Kirchhoff's and Ohm's laws, respectively, such an approach can be extremely fast and energy-efficient, provided that memory devices are dense and their conductances are adjustable (i.e., multi-state). The energy efficiency in part comes from performing “in-memory" computing that reduces the amount of data (corresponding to the synaptic weights) that are moved across or in-and-out of the chip during computation. Such communication overhead could dominate the energy consumption in the most advanced digital CMOS implementations.

The general challenge toward practical adoption of such circuits, especially when using the most prospective emerging memory technologies, is variations in *I*-*V* characteristics, e.g., in the switching voltages applied to change the memory state. In light of this challenge, the most straightforward application is ex-situ trained inference accelerators for the earlier firing-rate neural networks (Bavandpour et al., 2018), i.e., the so-called second generation of artificial neural networks (ANNs) with graded-response neurons. In such applications, memory devices are updated infrequently, only when new inference functionality should be programmed. Thus, crosspoint devices' conductances can be tuned with slower, more tolerant to device variations write schemes. For example, after the weights have been found in the software, memory cells are programmed, one by one, using feedback write-verify algorithms that can adapt to the unique *I*-*V* characteristics of each device (Alibart et al., 2012). For the same reason, the switching endurance, i.e., the number of times the memory devices can be reliably programmed, and the write speed/energy are less critical. Additionally, VMM operations in the inference of many neural networks could be performed with moderate, less than 8-bit precision, without incurring accuracy loss (Yang and Sze, 2019), which further relaxes requirements for analog properties and permits more *I*-*V* non-idealities and noise.

The most advanced neuromorphic inference circuits have been demonstrated with more mature floating-gate transistor memory circuits. Up until recently, such circuits were implemented primarily with “synaptic transistors" (Diorio et al., 1996), which may be fabricated using the standard CMOS technology, and several sophisticated, efficient systems were demonstrated (Chawla et al., 2004; Hasler and Marr, 2013; George et al., 2016). However, these devices have relatively large areas (>10^3^ *F*^2^, where *F* is the minimum feature size), leading to higher interconnect capacitance and hence larger time delays. More recent work focused on implementing mixed-signal networks with much denser (~40 F^2^) commercial NOR-flash memory arrays redesigned for analog computing applications (Bayat et al., 2015; Guo et al., 2017b). For example, a prototype of a 100k+-cell two-layer perceptron network fabricated in a 180-nm process with modified NOR-flash memory technology was reported in Guo et al. (2017a). It performed reliably, with negligible long-term drift and temperature sensitivity, and reproducible classification of the MNIST benchmark set images with ~95% fidelity and sub-1-μs time delay and sub-20-nJ energy consumption per pattern. The energy-delay product was six orders of magnitude better than the best (at that time) 28-nm digital implementation performing the same task with a similar fidelity (Guo et al., 2017a).

Recent theoretical studies showed that neuromorphic inference circuits could be also implemented with much denser 3D-NAND flash memories (Bavandpour et al., 2019, 2020; Lee et al., 2019), projected to scale eventually to 10 terabits per square inch density. In the long term, the most promising are perhaps circuits based on metal-oxide resistive switching random access (ReRAM for short, which are also called metal-oxide memristors) (Yang et al., 2013; Yu, 2018), especially their passively integrated (0T1R) technology variety (Kim et al., 2019). Indeed, due to the ionic switching mechanism, ReRAM devices with dimensions below 10 nm still retain excellent analog properties and year-scale retention (Govoreanu et al., 2013). Furthermore, a low-temperature fabrication budget allows monolithic vertical integration of multiple ReRAM crossbar circuits, further increasing effective density (Adam et al., 2017). There has been rapid progress in scaling up the complexity of ReRAM-based neuromorphic circuit demonstrations over the past several years (Prezioso et al., 2015; Bayat et al., 2018; Hu et al., 2018b; Kim et al., 2019; Lin et al., 2020b; Liu et al., 2020b; Yao et al., 2020a). However, the ReRAM technology is still in much need of improvement. In addition to high device variations, another remaining issue is high write currents and operating conductances, which must be decreased by at least one order of magnitude to reduce the significant overhead of peripheral circuits (Kim et al., 2019).

The device requirements for training hardware accelerators are different and much more stringent. For instance, long retention is not required because weights are frequently updated. That allows using volatile memories in analog VMM circuits, such as interfacial memristors based on electron trapping/detrapping switching (Chu et al., 2014; Sheridan et al., 2017; Cai et al., 2019a) and solid-state-electrolyte memories (Fuller et al., 2019; Berggren et al., 2020; Yeon et al., 2020), or even capacitor-based memories controlling current via crosspoint transistors (Ambrogio et al., 2018). However, the toughest challenge is much higher computing and weight precision required for training operation and the need for efficient schemes for weight updates, which in turn necessitate drastically tighter device variations. The additional related requirement is that the change in device conductance upon applying the write pulse should not depend on its current state (the so-called linearity of update property). Otherwise, accurate conductance adjustment would require sending a unique write pulse based on the current device state, which would be hardly compatible with fast (in parallel) weight update.

Phase change memories have also been investigated as candidates for variable resistors in analog VMM circuits (Burr et al., 2015; Joshi et al., 2020), though their main drawback is significant drift in the conductive state over time. High write endurance, high density (with vertical 3D-NAND-like integrated structure), and long retention are demonstrated in 1T Ferroelectric RAM devices. There is much excitement about such devices' applications in training and inference accelerators (Ni et al., 2018), though their analog properties are probably inferior to ReRAM. The significant drawbacks of magnetic devices, such as magnetic tunnel junction memories, are smaller on/off current ratios, insufficient for practical VMM circuits, and poor analog properties for scaled-down devices (Grollier et al., 2020).

The potentials of using light for implementing fast and large-fanout interconnect and linear computations, such as multiply-and-add operation, have motivated photonic neuromorphic computing research (Hamley et al., 2019; Berggren et al., 2020; Goi et al., 2020; Shasti et al., 2021). Different implementation flavors, e.g., with fixed (Lin et al., 2019) and programmable (Tait et al., 2016; Shen et al., 2017; Hamerly et al., 2019; Ríos et al., 2019) functionalities, have been recently suggested in the context of modern neural networks. Specifically, Lin et al. (2019) reports a system of multiple 3D-printed optical layers, each being a mesh of regions (neurons) with specifically chosen transmission-reflection properties, which can perform pattern classification inference similar to the convolutional neural networks. By sending a coherent light with amplitude-encoded input, a useful computation is performed at the speed of light. Specifically, the light diffracts and interferes when passing through the optical system and is ultimately steered to the specific region at the output layer corresponding to the pattern class. Ríos et al. (2019), Hamerly et al. (2019), Shen et al. (2017), and Tait et al. (2016) report optical neuromorphic systems with configurable weights. The inputs are encoded in the light's energy, and the weights are encoded by optical attenuation in PCM devices in Ríos et al. (2019) so that a product is computed by passing the light via PCM device. Tait et al. (2016) proposes encoding inputs with light amplitude and uses specific frequency for different VMM inputs. The light from inputs is combined and passed to the frequency selective weight banks based on a microring resonator (MRR) that features metal heaters to perform multiplication. In particular, the MRR coupling (i.e., weight) is controlled via heating by adjusting currents supplied to each MRR. In these reconfigurable implementations, the product accumulation (i.e., the summation operations in the VMM) is performed by integrating the light-induced charges on the photodetector. A very aggressive time-division multiplexing scheme for calculating VMM in which both weights and inputs are encoded in the coherent light's amplitude is proposed in Hamerly et al. (2019). At one step of such scheme, the input light is fanned out into *n* channels and combined with the light-encoded *n* weights using a beam splitter and then sent to *n* homodyne photodetectors to compute *n* products in parallel. All-optical feed-forward inference based on Mach-Zehnder interferometer meshes utilizes single-valued decomposition for the weight matrix (Shen et al., 2017). Unitary matrix transformations are implemented with optical beam splitters and phase shifters, while the diagonal matrix is implemented with optical attenuators.

In principle, sub-aJ energy and sub-ps latency for a single multiply-and-add operation might be possible with optical computing (Hamley et al., 2019). However, the main challenge remains much large dimensions of the optical components and the very high I/O overhead of converting to and from optical domains (Hamley et al., 2019; Berggren et al., 2020; Shasti et al., 2021). The designs that rely on conversion to the electrical domain would be especially affected by poor integration density of optical devices due to larger electrical communication overheads, which were shown to overwhelm system-level performance of (much denser) ReRAM based circuits (Bavandpour et al., 2018). Optical systems would ultimately benefit from very wide (≫10,000) dot-products and/or utilizing deep time-division multiplexing to amortize the I/O overhead. However, the possible issues of nonlinearities in charge integration and utility of such wide dot-product computations remain unclear (Hamley et al., 2019).

*Stochastic Vector-by-Matrix Multiplication:* Computations performed by the brain are inherently stochastic, in that, e.g., substantially different neural responses are observed to the repeatable presentation of identical stimuli (Rolls and Deco, 2010). Such noisy operation is mimicked by probabilistic neural networks, such as Boltzmann machines (Hinton and Sejnowski, 1983) and deep belief neural networks (Hinton, 2009). In the simplest case, such a network is comprised of binary neurons that compute stochastic dot products, i.e., probabilistically generate output according to their pre-activation (dot-product) values.

The stochastic functionality can be realized at either the synapse or the neuron side. In the latter, more straightforward scenario, the neuron first computes a dot-product of its inputs and corresponding weights deterministically. The result is then passed to some “probabilistic" activation function, e.g., used as an argument in the sigmoid probability function, to determine the probability of generating high output. Because of the typically large (> 100) ratio of synapses to neurons, the efficient deterministic dot-product implementations, e.g., with the already discussed analog VMM circuits, is of primary importance for realizing high-performance probabilistic neural network hardware. Still, earlier work showed that even the simplest, deterministic neurons may incur substantial overhead, e.g., occupy up to 30% of the area and consume up to 40% of energy for some neural network models (Bavandpour et al., 2018). Hence neuromorphic hardware would also benefit from the efficient realization of stochastic neurons.

Emerging devices can be broadly employed in two ways to achieve stochastic functionality, namely by using either dynamic or static *I*-*V* characteristics of memory devices. Specifically, the former approach is to utilize intrinsically stochastic switching between memory states in emerging memory devices. For example, in MTJ memories, thermal fluctuation causes stochastic transition between the low resistance parallel and high resistance antiparallel states so that the probability of the final memory state upon switching could be controlled by the spin-torque current (Grollier et al., 2020). The melt-quench-induced reconfiguration of the atomic structure is intrinsically stochastic in phase-change memories (PCMs) (Tuma et al., 2016). These phenomena were suggested for implementing MTJ (Ostwal et al., 2018) and PCM (Tuma et al., 2016) stochastic neurons. The second approach is to utilize intrinsic and extrinsic current fluctuations in memory devices, e.g., random telegraph (Cai et al., 2020a) and thermal noise (Mahmoodi et al., 2009) in ReRAM devices, or shot-noise in nanoscale floating gate transistors (Mahmoodi et al., 2009, 2019). In such an approach, the noisy current flowing into the neuron is compared against a reference value, e.g., using a simple latch, to implement a probabilistic activation function (Mahmoodi et al., 2019).

The primary concern for the former approach is the limited endurance of many memories and the drift in the stochastic switching properties upon repeated switching. An additional drawback is a necessity for the co-integration of multiple memory device technologies for scalable stochastic dot-product circuits, e.g., integrating ReRAM-based artificial synapses and MTJ-based neurons. On the other hand, analog circuits based on ReRAM devices only (Figure 10), though operating at a much lower signal-to-noise ratio (SNR), can be utilized to implement stochastic VMM of the second approach. Furthermore, adjusting read voltages in such a circuit allows for controlling SNR. Hence, the control of effective temperature, i.e., the slope of sigmoid probability function, enables efficient implementation of stochastic annealing in Boltzmann machines during runtime. The second approach's possible downside is slower operation because of lower read currents (which can be potentially addressed by utilizing external noise instead Mahmoodi et al., 2019). Finally, the impact of noise quality on functional performance is another common concern. This issue has not been systematically studied yet, though Gaussian-like thermal or shot noise should be more advantageous for truly random operation.

*Spiking Neuron and Synaptic Plasticity:* Despite much recent progress in algorithms (Neftci et al., 2019; Tavanaei et al., 2019), the most biologically plausible, spiking neural networks (SNNs) (Gerstner and Kistler, 2002) are still inferior in the functional performance to simpler ANNs. If simpler ANNs would remain superior, the work of efficient SNN hardware could still be justified by the need to efficiently interface to the brain and/or model it, which in turn could lead to the development of higher-cognition artificial intelligence algorithms. An additional intriguing feature of SNNs is local weight update rules, requiring only information from pre- and post-synaptic neurons that could enable large-scale neuromorphic hardware with real-time training capabilities (Thakur et al., 2018).

In the simplest SNN models, the information is encoded in spike-time correlations (Gerstner and Kistler, 2002), while the network function is defined by the synaptic weights, which are adjusted based on the relative timing of spikes that are passed via synapses. In addition to VMM, the essential operations in SNNs are leaky-integrate-and-fire (LIF) functions performed by neurons and various types of synaptic plasticity, such as short-term plasticity (STP) and long-term potentiation (LTP), and spike-timing-dependent-plasticity (STDP) (Gerstner and Kistler, 2002). LIF neurons mimic the dynamic processes in the neuronal membrane, while synaptic plasticities mimic learning and memory mechanisms in biological networks. For example, STP is a temporary change in the synaptic strength implementing a short-term memory. Without immediate reinforcement of synaptic weight adjustment, the memory would be lost, i.e., the synaptic weight would relax to the original equilibrium state. On the other hand, the frequently repeated spiking stimulus causes long-term memory, e.g., permanent potentiation via the LTP mechanism. STDP is a time-dependent specialization of Hebbian learning. Its specific goal is to strengthen the synaptic efficiency when pre- and post- synaptic spikes happen in the expected causal temporal order and weaken it otherwise.

A compact implementation of LIF neurons with biological, ms-scale integration times using conventional circuit technology is challenging because of the large capacitors that are required. Leaky integration circuits utilizing volatile memristors (e.g., based on filamentary Zhang et al., 2018b, interfacial Lashkare et al., 2018, and Mott insulator Adda et al., 2018 switching mechanisms) have been suggested to address this problem. In such implementations, the integrated current is encoded with a conductive state of the volatile memory device. Neuron spiking functionality was demonstrated with threshold-switching (volatile) memory devices that feature S-type negative differential resistance (NDR) *I*-*V* characteristics (Pickett et al., 2013). This approach's general idea is similar to the oscillator circuits based on S-type (NDR) device connected to a resistor-capacitor circuit (Kesim, 2019). LIF neurons based on spin-torque magnetic memories were simulated in Sengupta et al. (2016). In such a neuron, spin-torque oscillations are employed to generate spikes, while incremental magnetization and its relaxation mimic integration and leakage, respectively.

STP to LTP transition has been emulated with solid-state-electrolyte devices see, e.g., original work in Ohno et al. (2011) and more recent work on “diffusive" memristors (Wang et al., 2017). Specifically, the short and infrequent write pulses result in the formation of thin filaments, which are unstable and quickly dissolve, representing a short memory. However, a thicker and more stable filament can be formed by applying repeated and/or longer write pulses, thus mimicking transition to the LTP. Different STDP window implementations, e.g., using PCM (Kuzum et al., 2011) or metal-oxide ReRAM (Prezioso et al., 2016) devices, have been suggested by carefully selecting the shape of pre and post-synaptic write voltage pulses—see a comprehensive review of the emulated synaptic plasticity with memristive devices in Serrano-Gotarredona et al. (2013) and Saighi et al. (2015).

Several small-scale spiking neuromorphic systems based on emerging device technologies were demonstrated, including coincidence detection via STDP mechanism based on metal-oxide memristors (Pedretti et al., 2017; Prezioso et al., 2018) and temporal data classification with diffusive memristors (Wang et al., 2018c). However, the overall progress in such advanced hardware has been much slower compared to simpler ANNs inference accelerators. The main reason is more demanding functionality from emerging devices in such applications and hence the more severe impact of device variations on the SNN operation and performance. For example, SNNs rely on fixed-magnitude spikes to update the conductance of multiple devices in parallel. Because of that, change in the conductances could vary drastically even with minor variations in *I*-*V*'s switching voltages, which in turn leads to very significant variations in STDP characteristics (Prezioso et al., 2018). On the other hand, as already mentioned above, the implementation of simpler *ex-situ* trained ANNs is much less challenging because the write amplitude voltages in such networks can be adjusted uniquely for each device based on the feedback information during conductance tuning (Alibart et al., 2012).

Superconductor circuits, e.g., based on rapid single flux quantum (RSFQ) variety (Likharev and Semenov, 1991), are naturally suited for spiking circuits due to information encoding in SFQ voltage pulses. For example, Josephson Junction spiking neurons operating at up to 50GHz range have been demonstrated in Segall et al. (2017). The historical challenges of such an approach include inferior fabrication technology (which may finally change given the enormous investments in superconductor quantum computing), the low-temperature operation that limits its applications, and the lack of efficient analog memory circuits (Likharev, 2012). The photonic spiking neural networks (e.g., Feldmann et al., 2019) and hybrid superconductor/optoelectronic neuromorphic circuits (Buckley et al., 2017) share the same challenges of the already discussed photonic neuromorphic inference approaches.

*Reservoir Computing:* Due to intrinsic memory properties, recurrent neural networks, such as Google Neural Machine Translation model, are especially suitable for processing sequential or temporal data. Reservoir computing (RC) networks are a special type of efficiently learning recurrent networks (Lukǒevičius and Jaeger, 2009), that were motivated by cortical information processing (Maass et al., 2004). Among its variants are liquid state machines (Maass et al., 2002), which is a spiking RC network, and echo state networks (Jaeger, 2001), an RC based on a very sparse recurrent network. The main component in RC networks is a reservoir, which is a nonlinear recurrent network that maps inputs into a higher-dimensional spatio-temporal representation and has the property of a fading memory of the previous inputs and network states. Another component is a readout layer, which maps the intermediate state to the outputs. All connections in the reservoir are fixed and only weights in the readout layer are trainable. Because of that and sparse intermediate representation, faster and online algorithms can be employed for training such networks, which is a primary strength of this approach.

Though both readout and the reservoir can also be realized with the discussed analog VMM circuits, intriguing opportunities for implementing the reservoir are presented by nonlinear physical phenomena in superconductor, magnetic, and photonic devices (Tanaka et al., 2019). For example, spoken vowel recognition was demonstrated with RC in which the reservoir was implemented with four coupled MTJ-based spin-torque oscillators (STO) (Romera et al., 2018). In such a demo, the temporal input corresponding to spoken vowels is first converted to the frequency domain, which is in turn mapped to the corresponding DC bias currents that are applied to the MTJ devices. The induced voltage on the STO devices is used as an output of the reservoir. The reservoir utilizes the nonlinear dependence of the frequency of STOs on the DC current and the history-dependent transient motions of the MTJ's free layer spins spin.

Various photonic reservoirs have been suggested (Shasti et al., 2021), e.g., utilizing transient properties of optical systems with time-delayed feedback (Bruiner et al., 2013), or relying on superimposing lights that passively circulates via waveguides, splitters and combiners, and nonlinear conversion to the electronic domain (Vandoorne et al., 2014), to achieve high-dimensional response. The dynamics in the superconductor circuits are recently studied for efficient and extremely fast reservoir implementation (Rowlands et al., 2021). Specifically, the proposed reservoir is based on a Josephson transmission line (JTL) formed by a chain of biased JJs. An input pulse from one end of the JTL causes a rapid cascade of junction phase slips that propagate SFQ pulse to the other end. Because JJs modulate each others' currents, a complex dynamical state is achieved.

There are several general concerns with RC computing approaches. On the algorithmic level, RC is inferior in performance to state-of-the-art approaches and it is unclear whether without further algorithm improvements such a handicap can be outweighed by the advantages of online training. The main concern for various hardware implementations is again related to the device variations, e.g., whether the hardware would be able to produce repeatable results when applying the same input. An additional concern for magnetic devices is the limited coupling between devices which could impact the effectiveness of the reservoir.

*Hyperdimensional Computing/Associative Memory:* Hyperdimensional computing (Kanerva, 2009) circuits have been recently demonstrated with ReRAM (Li et al., 2016a) and PCM (Karunaratne et al., 2020) devices. The low-level operation in hyperdimensional computing is closely related to that of associative or content addressable memory (Hertz et al., 1991). Specifically, at the core of such an approach is an associative memory array circuit that outputs the closest, in a Hamming distance sense, memory row entry to a binary input vector serving as a search key. Assuming symmetric binary representation, with −1 and +1 encoding, Hamming distance is linearly related to a dot product, i.e., equal to output vector length minus dot product between the input vector and the stored memory row values. Therefore, the critical functionality in hyperdimensional computing is again a VMM operation. After the VMM operation has been completed, its results are passed to the winner-take-all circuit (Hertz et al., 1991) (which is a harder version of a softmax function; Bridle, 1989) that determines the element with the smallest Hamming distance while discarding all other outputs. The additional simplification is that both input and weights in VMM are binary.

In principle, binary VMM can be more efficiently implemented in hardware than its fully analog version. Similar to binary neural networks (Simons and Lee, 2019), the apparent tradeoff is a worse functional performance of hyperdimensional computing. Another essential feature of hyperdimensional computing is the suitability for fast “one-shot" or incremental learning (Kanerva, 2009) though at the cost of having a much more redundant memory array. Note that fast “one-shot” learning is not unique to hyperdimensional computing. For example, Hebbian learning and its many variants used in training associative neural networks have recursive form and are naturally incremental in that the weights can be modified only based on current weight values and the new pattern stored in the network (Hertz et al., 1991).

*Concluding Remarks:* Many emerging devices and circuit technologies are currently being explored for neuromorphic hardware implementations. Neuromorphic inference accelerators utilizing analog in-memory computing based on floating gate memories are perhaps the closest to widespread adoption, given the maturity of such technology, the practicality of its applications, and competitive performance as compared to conventional (digital CMOS) circuit implementations. Comparing the performance prospects of other neuromorphic approaches is not straightforward because many proposals target algorithms with inferior functional performance, especially those closely mimicking the brain's operation. Baring a substantial breakthrough in ML algorithms or the emergence of new applications that could benefit from high-performance low-accuracy neuromorphic hardware, the inferior functional performance may limit the practicality of other approaches. The main challenge, much more so for advanced neuromorphic computing concepts, remains significant variations in the operation of emerging devices.

## 5. Outlook

This report has laid out exciting applications of fast ML to enable scientific discovery across a number of domains. This is a rapidly developing area with many exciting new studies and results appearing often. However, this is a relatively young area rich with potential and a number of open challenges across a number of fields. Beyond what has been laid out in the report, we hope that the discussion of scientific use-cases and their overlaps will provide readers with the inspiration to entertain and pursue additional applications.

In Section 4, we provided an overview of techniques for developing powerful ML algorithms that need to be operated in high throughput and low latency environments. This includes both system design and training as well as efficient deployment and implementation of those ML models. Implementation in hardware is discussed under two main categories—current conventional CMOS and more speculative beyond CMOS technologies. In the conventional CMOS case, in light of the end of Moore's Law, the recent emphasis has been focused on advanced hardware architectures designed for ML. We gave an overview of popular and emerging hardware architectures and their strengths and shortcomings. A key area of importance for the multitude of hardware is their codesign of a given ML algorithm for specific hardware including the architecture and programmability of that algorithm. An example of a particularly relevant and important hardware platform is for FPGAs and that is the use-case discussed in Section 4.4. Finally, we concluded with an overview of beyond CMOS technologies which offer exciting and ultra-efficient technologies on which we can implement ML models. While these technologies are speculative, they offer potential orders of magnitude improvement over conventional technologies.

Both ML training and deployment techniques and computer architectures are extremely rapidly moving fields with new works appearing at a pace difficult to keep up with, even for this report. While new methods are being introduced continuously in both spaces, it is particularly important to understand the codesign of new algorithms for different hardware and the ease of use of the tool flows for deploying those algorithms. Innovations here will allow rapid and broad adoption of powerful new ML hardware. In the case of beyond CMOS technologies, these practical considerations are important as well as considering the maturity of the technology, integration into computing architectures, and how to program such devices.

We look forward to revisiting these topics in the near future to see how quickly advances may come in applications, ML techniques, and hardware platforms—and most importantly their confluence to enable paradigm-shifting breakthroughs in science.

## Author Contributions

All authors listed have made a substantial, direct, and intellectual contribution to the work and approved it for publication.

## Conflict of Interest

MB was employed by the company Xilinx Inc. The remaining authors declare that the research was conducted in the absence of any commercial or financial relationships that could be construed as a potential conflict of interest. The handling editor EC is currently organizing a Research Topic with the authors JD, ML, and JN.

## Publisher's Note

All claims expressed in this article are solely those of the authors and do not necessarily represent those of their affiliated organizations, or those of the publisher, the editors and the reviewers. Any product that may be evaluated in this article, or claim that may be made by its manufacturer, is not guaranteed or endorsed by the publisher.

## Footnotes

^1^ fastmachinelearning.org

^2^The DAMA/NaI and subsequent DAMA/LIBRA experiment, claim the direct observation of DM particles in the galactic halo (Bernabei et al., 2013), but the results are in tension with negative results from similar experiments (Schumann, 2019).

^3^In these comparisons, we treat HBM and HBM2 as external memory as it is used in the same way as DDR4 or GDDR memory.
