# Corrigendum: Applications and techniques for fast machine learning in science

**DOI:** 10.3389/fdata.2023.1301942

**Published:** 2023-10-16

**Authors:** Allison McCarn Deiana, Nhan Tran, Joshua Agar, Michaela Blott, Giuseppe Di Guglielmo, Javier Duarte, Philip Harris, Scott Hauck, Mia Liu, Mark S. Neubauer, Jennifer Ngadiuba, Seda Ogrenci-Memik, Maurizio Pierini, Thea Aarrestad, Steffen Bähr, Jürgen Becker, Anne-Sophie Berthold, Richard J. Bonventre, Tomás E. Müller Bravo, Markus Diefenthaler, Zhen Dong, Nick Fritzsche, Amir Gholami, Ekaterina Govorkova, Dongning Guo, Kyle J. Hazelwood, Christian Herwig, Babar Khan, Sehoon Kim, Thomas Klijnsma, Yaling Liu, Kin Ho Lo, Tri Nguyen, Gianantonio Pezzullo, Seyedramin Rasoulinezhad, Ryan A. Rivera, Kate Scholberg, Justin Selig, Sougata Sen, Dmitri Strukov, William Tang, Savannah Thais, Kai Lukas Unger, Ricardo Vilalta, Belina von Krosigk, Shen Wang, Thomas K. Warburton

**Affiliations:** ^1^Department of Physics, Southern Methodist University, Dallas, TX, United States; ^2^Fermi National Accelerator Laboratory, Batavia, IL, United States; ^3^Department of Electrical and Computer Engineering, Northwestern University, Evanston, IL, United States; ^4^Department of Materials Science and Engineering, Lehigh University, Bethlehem, PA, United States; ^5^Xilinx Research, Dublin, Ireland; ^6^Department of Computer Science, Columbia University, New York, NY, United States; ^7^Department of Physics, University of California, San Diego, San Diego, CA, United States; ^8^Massachusetts Institute of Technology, Cambridge, MA, United States; ^9^Department of Electrical and Computer Engineering, University of Washington, Seattle, WA, United States; ^10^Department of Physics and Astronomy, Purdue University, West Lafayette, IN, United States; ^11^Department of Physics, University of Illinois Urbana-Champaign, Champaign, IL, United States; ^12^European Organization for Nuclear Research (CERN), Meyrin, Switzerland; ^13^Karlsruhe Institute of Technology, Karlsruhe, Germany; ^14^Institute of Nuclear and Particle Physics, Technische Universität Dresden, Dresden, Germany; ^15^Lawrence Berkeley National Laboratory, Berkeley, CA, United States; ^16^Department of Physics and Astronomy, University of Southampton, Southampton, United Kingdom; ^17^Thomas Jefferson National Accelerator Facility, Newport News, VA, United States; ^18^Department of Electrical Engineering and Computer Sciences, University of California, Berkeley, Berkeley, CA, United States; ^19^Department of Computer Science, Technical University Darmstadt, Darmstadt, Germany; ^20^Department of Bioengineering, Lehigh University, Bethlehem, PA, United States; ^21^Department of Physics, University of Florida, Gainesville, FL, United States; ^22^Department of Physics, Yale University, New Haven, CT, United States; ^23^Department of Engineering and IT, University of Sydney, Camperdown, NSW, Australia; ^24^Department of Physics, Duke University, Durham, NC, United States; ^25^Cerebras Systems, Sunnyvale, CA, United States; ^26^Birla Institute of Technology and Science, Pilani, India; ^27^Department of Electrical and Computer Engineering, University of California, Santa Barbara, Santa Barbara, CA, United States; ^28^Department of Physics, Princeton University, Princeton, NJ, United States; ^29^Department of Computer Science, University of Houston, Houston, TX, United States; ^30^Department of Physics, Universität Hamburg, Hamburg, Germany; ^31^Department of Physics and Astronomy, Iowa State University, Ames, IA, United States

**Keywords:** machine learning for science, big data, particle physics, codesign, coprocessors, heterogeneous computing, fast machine learning

In the published article, there was an error regarding the affiliation for author Anne-Sophie Berthold. Instead of having affiliation 25 (Cerebras Systems, Sunnyvale, CA, United States) they should have 14 (Institute of Nuclear and Particle Physics, Technische Universität Dresden, Dresden, Germany).

The authors apologize for this error and state that this does not change the scientific conclusions of the article in any way. The original article has been updated.

